# Enantiomeric Lignans and Neolignans from *Phyllanthus glaucus*: Enantioseparation and Their Absolute Configurations

**DOI:** 10.1038/srep24809

**Published:** 2016-04-29

**Authors:** Zhaodi Wu, Yongji Lai, Lei Zhou, Ye Wu, Hucheng Zhu, Zhengxi Hu, Jing Yang, Jinwen Zhang, Jianping Wang, Zengwei Luo, Yongbo Xue, Yonghui Zhang

**Affiliations:** 1 Hubei Key Laboratory of Natural Medicinal Chemistry and Resource Evaluation, School of Pharmacy, Tongji Medical College, Huazhong University of Science and Technology, Wuhan 430030, Hubei Province, People’s Republic of China; 2 Department of Pharmacy, the Central Hospital of Wuhan, Wuhan 430014, Hubei Province, People’s Republic of China; 3 State Key Laboratory of Phytochemistry and Plant Resources in West China, Kunming Institute of Botany, Chinese Academy of Sciences, Kunming 650204, People’s Republic of China; 4 Tongji Hospital Affiliated to Tongji Medical College, Huazhong University of Science and Technology, Wuhan 430030, People’s Republic of China

## Abstract

Eight pairs of enantiomeric neolignans, norlignans, and sesquineolignans (**1a/1b**–**8a**/**8b**), together with five known neolignans (**9a/9b** and **10**–**12**), have been isolated from 70% acetone extract of the whole plants of *Phyllanthus glaucus* Wall. (Euphorbiaceae). The racemic or partial racemic mixtures were successfully separated by chiral HPLC using different types of chiral columns with various mobile phases. Their structures were elucidated on the basis of extensive spectroscopic data. The absolute configurations of **2a**/**2b** were determined by computational analysis of their electronic circular dichroism (ECD) spectrum, and the absolute configurations of other isolates were ascertained by comparing their experimental ECD spectra and optical rotation values with those of structure-relevant compounds reported in literatures. Compounds **4a**/**4b** featured unique sesquineolignan skeletons with a novel 7-4′-epoxy-8′-8′′/7′-2′′ scaffold, consisting of an aryltetrahydronaphthalene and a dihydrobenzofuran moiety. The planar structures of compounds **2**, **3**, **7**, and **8** were documented previously; however, their absolute configurations were established for the first time in this study. The antioxidant activities of **1a**/**1b**–**8a**/**8b** were evaluated using DPPH free radical scavenging assay, and the results demonstrated that compounds **1b** and **3b** showed potent DPPH radical scavenging activities with IC_50_ values of 5.987 ± 1.212 and 9.641 ± 0.865 *μ*g/mL, respectively.

Lignans and neolignans are biosynthetically generated from phenylpropanoids, depending on the ways they are linked^1^, which present a conspicuous chemical diversity and have multifarious clinical pharmacology activities^2^. In nature, lignans with chiral carbon atoms are usually composed of one enantiomer or of several stereoisomers with different amount, so they present optical activities in general^3^,^4^. In the past decades, the enantioseparation and absolute configurations of natural enantiomeric lignans and neolignans have attracted the attention of researchers around the world, with the aid of application of chromatographic enantioseparation, single-crystal X-ray diffraction, electronic circular dichroism (ECD) techniques^5^,^6^,^7^,^8^.

Plants of the genus *Phyllanthus* (Euphorbiaceae) are widely distributed in most tropical and subtropical areas, which have been used in folk medicine for a long time to treat kidney and urinary bladder disturbances, intestinal infections, diabetes, and hepatitis B^9^. Previous phytochemical investigations on plants of this genus led to the isolation of terpenoids^10^,^11^,^12^,^13^, lignans^14^, flavonoids^15^, alkaloids^16^, and tannins^17^. In the course of searching for bioactive natural products from the traditional Chinese medicine plants^18^,^19^,^20^, our attention was drawn to *P. glaucus*, a kind of deciduous shrub widely distributed in south China. The roots of the plant are commonly used to treat rheumatic arthritis and infantile malnutrition by the local people^21^. To the best of our knowledge, phytochemical study of *P. glaucus* had resulted in the isolation of 33 compounds including two new lignan glucosides by Yu and co-workers^21^. In our present study, eight pairs of enantiomers (**1a**/**1b**–**8a**/**8b**, Fig. 1), including a pair of enantiomeric neolignans (**1a/1b**), two pairs of norlignans (**2a/2b** and **3a**/**3b**), and five pairs of sesquilignans (**4a**/**4b**–**8a**/**8b**), along with five known compounds (**9a**/**9b**, **10**, **11**, and **12**) were isolated from the acetone extract of the whole plants of *P. glaucus*. The enantioseparations of these compounds were achieved using chiral HPLC approaches, and their structures including absolute configurations were elucidated by extensive spectroscopic analyses and ECD calculations. It is notable that compounds **4a**/**4b** possess unique sesquineolignan architecture with 7-4′-epoxy-8′-8′′/7′-2′′ scaffolds, consisting of an aryltetrahydronaphthalene and a dihydrobenzofuran moiety. In addition, the antioxidant activities of compounds **1a**/**1b**–**8a**/**8b** were evaluated by DPPH assay.

## Results and Discussion

### Structural Elucidation of Compounds 1a/1b–8a/8b

Compound **1** was isolated as a colorless gum, and its molecular formula was determined to be C_20_H_24_O_7_, as elucidated by HRESIMS (*m*/*z* 399.1407, calcd for [M + Na]^+^ 399.1420) and ^13^C NMR data (Table 1), requiring nine degrees of unsaturation. The IR spectrum of **1** showed the presence of hydroxyl (3418 cm^−1^) and aromatic functionalities (1614, 1518, and 1461 cm^−1^). The ^1^H NMR spectrum revealed the co-existence of a symmetrical 1,3,4,5-tetrasubstituted aromatic ring at *δ*_H_ 6.71 (2H, s), an asymmetrical 1,3,4,5-tetrasubstituted aromatic ring at *δ*_H_ 6.60 (1H, s) and 6.57 (1H, s), and two methoxyl groups at *δ*_H_ 3.81 (6H, s, each). The ^13^C NMR spectra (Table 1) of **1** displayed 20 carbons including two methoxyl groups, four sp^3^ methylenes, two sp^3^ methines, and two aromatic rings. The above mentioned data, together with the ^1^H–^1^H COSY correlations of H-8 [*δ*_H_ 3.45 (1H, ddd, *J* = 7.7, 6.0, 5.3 Hz)] with H-9 [*δ*_H_ 3.85 (1H, dd, *J* = 10.9, 5.3 Hz, H-9a), and 3.76 (1H, dd, *J* = 10.9, 7.7 Hz, H-9b)] and H-7 [*δ*_H_ 5.51 (1H, d, *J* = 6.0 Hz)], and of H-8′ [*δ*_H_ 1.79 (2H, m)] with H-9′ [*δ*_H_ 3.56 (2H, t, *J* = 6.5 Hz)] and H-7′ [*δ*_H_ 2.56 (2H, t, *J* = 7.6 Hz)], suggested that compound **1** was a lignan resembled cedrusin^22^. This prediction was further confirmed by the HMBC correlations from H-7 to C-9, C-4′, and C-5′, from H-8 to C-1, C-4′, and C-5′, and from H-7′ to C-1′, C-2′, C-6′, and C-9′. In addition, the two methoxyl groups were located at C-3 and C-5 by the HMBC interactions from protons at *δ*_H_ 3.81 (6H, s) to carbons at *δ*_C_ 149.3 (C-3 and C-5). Thus, the planar structure of **1** was established and named phyllanglaucin A.

**Figure 1:**
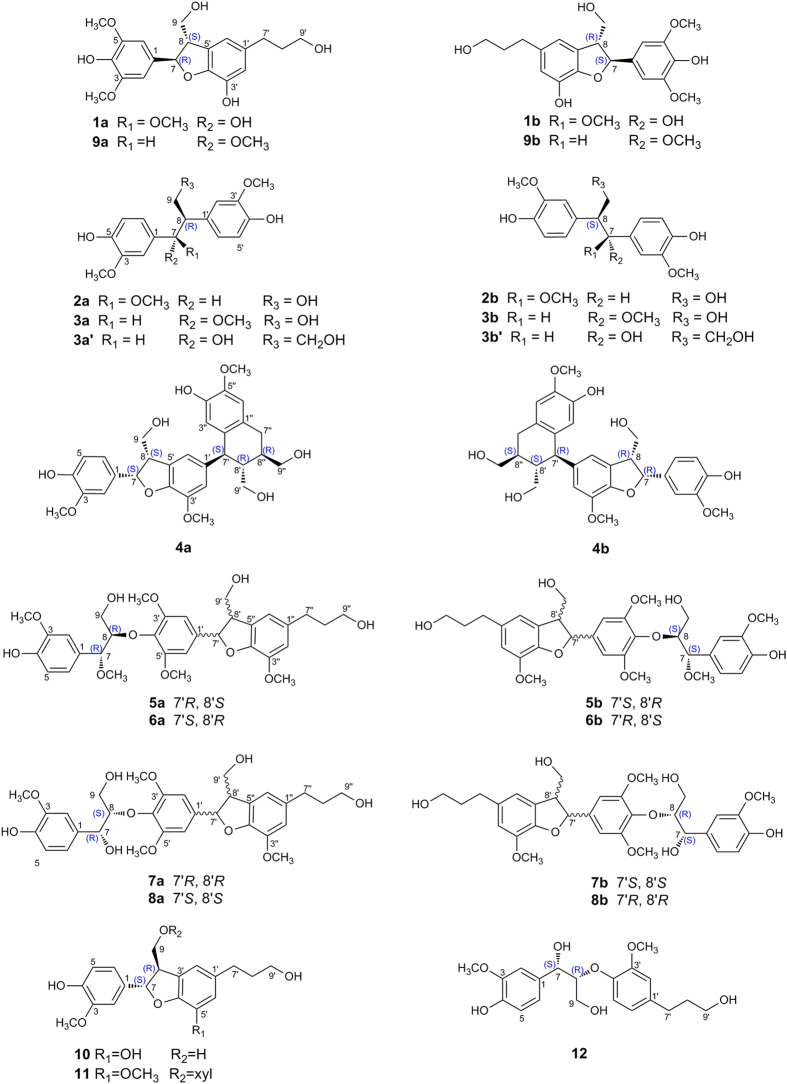
Structures of the co-isolated compounds.

**Figure 2:**
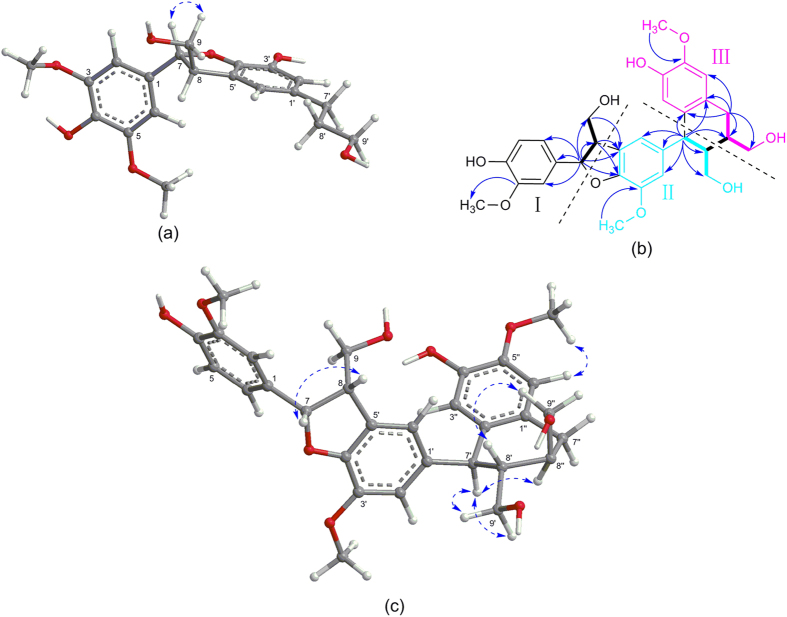
(**a**) Key NOESY correlations of compound **1**; (**b**) ^1^H**–**^1^H COSY and key HMBC correlations of compound **4**; (**c**) Key NOESY correlations of compound **4**.

**Figure 3:**
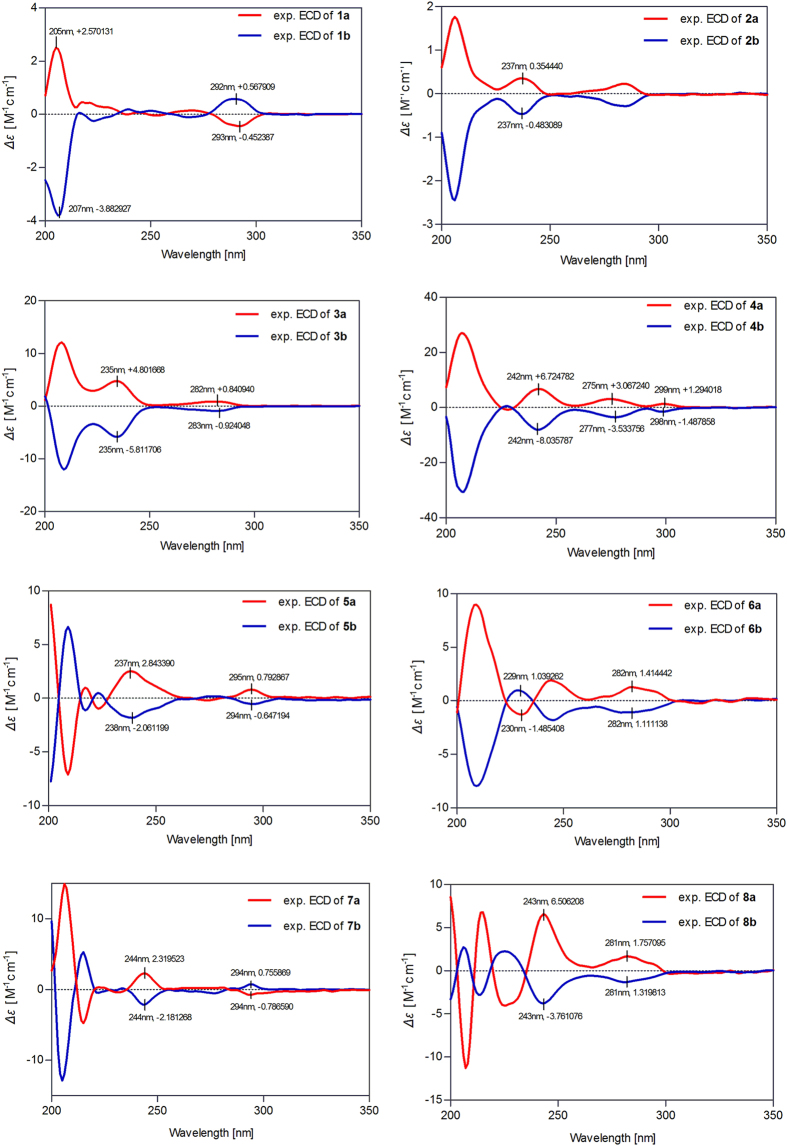
Experimental ECD spectra of 1a/1b–8a/8b (5b and 6a/6b recorded in MeCN; the others in MeOH).

The coupling constant between H-7 and H-8 (*J*_7,8_ = 6.0 Hz) of **1** suggested that the preferred conformation of the two protons was *trans*^23^, which was further supported by the NOESY correlations of H-7 with H-9b (Fig. 2a). Interestingly, we found that **1** was a racemic mixture for the presence of two peaks on the chiral HPLC. In order to define their absolute configurations, ECD, a powerful and effective method in determining the absolute configuration of natural products, was applied. Performing the enantioseparation of **1** by HPLC using a chiral-pak IA column provided the enantiomers **1a** and **1b** with a ratio about 1:3. Given the reversed helicity rule of the ^1^L_b_ band ECD for the 7-hydroxy-2,3-dihydrobenzo[*b*]furan chromophore^24^, the negative ^1^L_b_ Cotton effect (CE) of **1a** around 293 nm (Δ*ε* − 0.45) (Fig. 3) suggested a 7*R* configuration, and accordingly, the positive CE of **1b** at 292 nm (Δ*ε* + 0.57) (Fig. 3) indicated a 7*S* configuration. Thus, compounds **1a** and **1b** were determined as (+)-(7*R*,8*S*)-phyllanglaucin A and (−)-(7*S*,8*R*)-phyllanglaucin A (Fig. 1), respectively.

Compound **2** was purified as an amorphous white powder, which showed IR absorptions for hydroxyl (3366 cm^−1^) and aromatic rings (1606 and 1518 cm^−1^). The molecular formula, C_18_H_22_O_6_, was determined by HRESIMS (*m*/*z* 357.1321, calcd for [M + Na]^+^ 357.1314) and the ^13^C NMR data. The ^1^H NMR spectrum of **2** displayed typical protons including six aromatic protons, two methines, one oxygenated methylene, and three methoxyls, and the ^13^C NMR and DEPT spectra resolved 18 carbon signals. The 1D NMR data (Table 1) suggested that **2** had the same planar structure as *threo*-2,3-*bis*-(4-hydroxy-3-methoxyphenyl)-3-methoxypropanol, a norlignan isolated from *Aralia bipinnata*^25^, which was further supported by detailed analyses of the 2D NMR spectra. In consequence, the structure of **2** was assigned as 4,4′-dihydroxy-3,7,3′-trimethoxy-7′,8′,9′-trinor-8,1′-neolignan-9-ol. The relative configuration of **2** was subsequently determined to be 7,8-*threo* due to the large coupling constant between H-7 and H-8 (*J*_7,8_ = 9.3 Hz). However, due to the lack of optical rotation and CE, compound **2** was proposed to be a racemic mixture. With the help chiral HPLC, the enantiomers of **2a** (5.3 mg) and **2b** (5.3 mg) were obtained with a ratio of 1:1, which showed the mirror image-like ECD curves (Fig. 3) and owned the opposite specific rotations (**2a**: 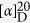 + 30.0; **2b**: 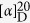 −30.8).

**Figure 4:**
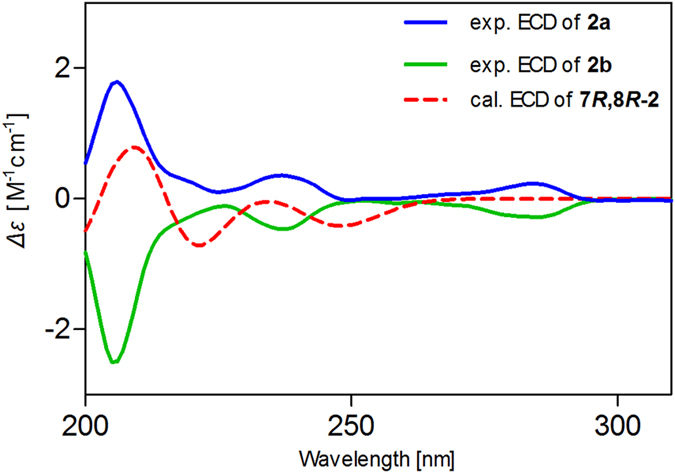
Assignment of the absolute configurations of 2 by comparison of the calculated ECD spectrum for (7*R,*8*R*)-2 with the experimental CD spectra for 2a/2b using TDDFT methods.

Although the planar structures of **2a/2b** were reported in several literatures^25^,^26^,^27^,^28^,^29^, their absolute configurations remain to be determined. Our efforts to obtain fine crystals to determine the absolute configuration directly by X-ray crystallography failed. Therefore, quantum chemical ECD calculations were applied^5^,^6^, which were carried out for compound 7*R*,8*R*-**2** at the level of LC-wPBE/6-311++G(2d,p)//B3LYP/6-311++G(2d,p) in MeOH with IEFPCM solvation model. The theoretical ECD curve of 7*R*,8*R*-**2** matched well with the experimental ECD curve of compound **2a** (Fig. 4). The result was further validated by our additional ECD calculations employing two other different functionals, i.e., CAM-B3LYP and WB97XD, in which consistent results of ECD were obtained (given in supplementary information). Thus, the absolute configuration of **2a** was unambiguously assigned as 7*R*,8*R*. Finally, compound **2a** was determined as (7*R*,8*R*)-4,4′-dihydroxy-3,7,3′-trimethoxy-8,1′-7′,8′,9′-trinor-neolignan-9-ol, and **2b** was determined as (7*S*,8*S*)-4,4′-dihydroxy-3,7,3′-trimethoxy-8,1′-7′,8′,9′-trinor-neolignan-9-ol.

Compound **3**, a white amorphous powder, had the same molecular formula (C_18_H_22_O_6_) as that of **2**, deducing from ^13^C NMR and DEPT and the positive HRESIMS experiment (*m/z* 357.1323 [M + Na]^+^, calcd 357.1314). A comparison of NMR data of **3** with that of **2** indicated that the significant difference was the coupling constant between H-7 and H-8 (*J*_7,8_ = 9.3 Hz of **2** and *J*_7,8_ = 6.6 Hz of **3**) (Table 1). These findings suggested that **3** was a stereoisomer of **2**, and it was determined to be *erythro* form in comparison with structurally related compounds reported in literatures^27^,^30^. Interestingly, compound **3** was also found to be a racemic mixture, and was further resolved to yield **3a** (4.5 mg) and **3b** (4.1 mg) over HPLC using a chiral column. **3a** and **3b** exhibited mirror image-like ECD curves (Fig. 3) and opposite specific rotations (**3a**: 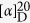 + 79.5; **3b**: 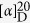 −76.9).

The absolute configurations of **3a** and **3b** were determined by comparing their optical rotations (**3a**: 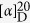 + 79.5; **3b**: 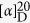 −76.9) with those of two similar compounds (1*S*,2*R*)-1,2-*bis*-(4-hydroxy-3-methoxyphenyl)-1,3-propanediol (**3a′**) and (1*R*,2*S*)-1,2-*bis*-(4-hydroxy-3-methoxyphenyl)-1,3-propanediol (**3b′**) (Fig. 1), whose absolute configurations were established by the glycosylation shift rule^31^ (**3a′**: 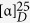  + 41.0 (c 0.7, MeOH); **3b′**: 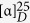 − 40.5 (c 1.2, MeOH). This conclusion was also supported by comparing the optical rotations with other analogues, such as carayensins B and C reported from *Carya cathayensis*^32^. Finally, **3a** was determined as (7*S*,8*R*)-4,4′-dihydroxy-3,7,3′-trimethoxy-8,1′-7′,8′,9′-trinor-neolignan-9-ol, and **3b** was determined as (7*R*,8*S*)-4,4′-dihydroxy-3,7,3′-trimethoxy-8,1′-7′,8′,9′-trinor-neolignan-9-ol.

Compound **4** was obtained as a colorless gum. The IR spectrum displayed absorption bands for hydroxyls (3339 cm^−1^), and aromatic rings (1654, 1603, 1515, 1496, and 1464 cm^−1^), and the molecular formula C_30_H_34_O_9_ was deduced by the HRESIMS ion peak at *m*/*z* 561.2089 [M + Na^+^] (calcd 561.2101), corresponding to fourteen degrees of unsaturation. The ^1^H NMR spectrum revealed the presence of an ABX system [*δ*_H_ 6.59 (1H, d, *J* = 8.2 Hz, H-5), 6.57 (1H, d, *J* = 1.8 Hz, H-2), and 6.42 (1H, dd, *J* = 8.2, 1.8 Hz, H-6)], four aromatic protons [*δ*_H_ 6.68 (1H, s, H-6′′), 6.62 (1H, s, H-3′′), 6.62 (1H, br s, overlapped, H-6′), and 6.38 (1H, br s, H-2′)], three methoxyl groups [*δ*_H_ 3.85 (3H, s), 3.72 (3H, s), and 3.46 (3H, s)], one oxymethine [*δ*_H_ 5.46 (1H, d, *J* = 2.1 Hz, H-7)], three oxygenated methylenes [*δ*_H_ 3.83 (1H, m, H-9a), 3.46 (1H, overlapped, H-9b), 3.67 (1H, m, H-9′a)], 3.51 (1H, dd, *J* = 11.2, 3.3 Hz, H-9′b), 3.70 (1H, m, H-9′′a), and 3.67 (1H, m, H-9′′b)] (Table 2). The ^13^C NMR and DEPT spectra displayed 30 carbon resonances attributable to three benzene rings, four methylenes (three oxygenated), five methines (one oxygenated), and three methoxyl groups. Three benzene rings accounted for twelve degrees of unsaturation, and the remaining two degrees of unsaturation revealed the presence of two additional rings.

Expansion of the formula to C_27_H_25_O_6_**·**(OMe)_3_ suggested that compound **4** was a sesquilignan. Moreover, three typical C6–C3 substructures (I: C1–C9, II: C1′–C9′, and III: C1′′–C9′′) were elucidated directly on the basis of COSY, HSQC, and HMBC spectra (Fig. 2). The connectivity of substructures I and II were established by the linkages of C7-*O*-C4′ and C8-C5′ to form a dihydrobenzofuran neolignan segment by the HMBC correlations from H-7 to C-4′, and C-5′, from H-8 to C-1, C-4′, and C-5′, and from H-9 to C-5′. The connectivity of substructures II and III were furnished by the direct linkages of C7′-C2′′ and C8′-C8′′ to construct an aryltetrahydronaphthalene neolignan moiety based on HMBC correlations from H-7′ to C-1′′, C-2′′, and C-8′′, and COSY correlations between H-8′ and H-8′′. In addition, HMBC correlations for CH_3_O-3/C-3, CH_3_O-3′/C-3′, and CH_3_O-5′′/C-5′′ revealed that these methoxyl groups were located at C-3, C-3′ and C-5′′, respectively. Thus, the planar structure of compound **4** was established as 3,3′,5′′-trimethoxy-4,4′′-dihydroxy-7,4′-epoxy-8,5′-7′,2′′-8′,8′′-sesquineolignan-9,9′,9′′-triol with an unprecedented sesquineolignan architecture, and was named phyllanglaucin B.

The relative configuration of **4** was determined by analyses of ^1^H–^1^H coupling constants and NOESY experiment. The small coupling constant of H-7/H-8 (*J*_7,8_ = 2.1 Hz) and the large coupling constant of H-7′/H-8′ (*J*_7′,8′_ = 8.8 Hz) suggested a *cis* configuration of H-7 and H-8 and a *trans* configuration of H-7′ and H-8′, respectively. The strong NOESY interactions from H-7′ to H-9′a and H-9′b confirmed the *trans* configuration of H-7′ and H-8′. Additional, NOESY correlation from H-7′ to H-8′′ revealed that H-7′ and H-8′′ was *cis*-oriented. Thus, the relative configuration of **4** was established as 7R*,8R* of the dihydrobenzofuran segment and 7′R*,8′S*,8′′S* of the aryltetrahydronaphthalene moiety.

Subsequent chiral HPLC resolution of **4** afforded the anticipated enantiomers **4a** (1.5 mg) and **4b** (7.5 mg), which showed mirror image-like ECD curves (Fig. 3) and specific rotations (**4a**: 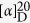 + 62.6; **4b**: 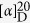 − 64.8). The absolute configurations of **4a** and **4b** were deduced by inspection of their ECD spectra. Generally, aryltetrahydronaphthalene lignans give two CEs in the ECD spectra at 280–290 nm and 230–245 nm, and the symbols of the two CEs are opposite^33^. On the basis of the published data^33^, CEs around 240 (+), 270 (+), and 290 (−) nm indicated the 7′*S*, 8′*R*, 8′′*R* configuration, in the case of **4a**, it exhibited CEs around 242 (Δ*ε* + 6.72), 275 (Δ*ε* + 3.07), and 299 (Δ*ε* + 1.29) nm, the positive CE at 299 nm in the ECD spectrum of **4a** was caused by *S* configuration at C-7, which produced a positive CE between 260–300 nm on the basis of the reversed helicity rule of the ^1^L_b_ band ECD for the 7-methoxy-2,3-dihydrobenzo[*b*]furan chromophore^24^. Namely, the positive rather than negative CE near 299 nm of **4a** was ascribed to the superposition of the Cotton effects produced by 7*S* and 7′*S*. Therefore, the absolute configuration of **4a** was established as 7*S*, 8*S*, 7′*S*, 8′*R*, 8′′*R*, and that of **4b** was 7*R*, 8*R*, 7′*R*, 8′*S*, 8′′*S*, which were given the trival names (+)-phyllanglaucin B and (−)-phyllanglaucin B, respectively.

Compounds **5** and **6** were obtained as a mixture of amorphous powder and displayed only one peak on the reversed-phase HPLC. However, the carbon signals at *δ*_C_ 145.2, 135.7, 131.0, 127.7, 103.4, 86.1 and 54.0 were all splited in the ^13^C NMR spectrum. In particular, there should have been a doublet signal at *δ*_H_ 4.60 (H-7) in the ^1^H NMR spectrum of the proposed structure (Fig. 1), but it actually exhibited a de-doublet signals with the coupling constants *J* = 2.8, 6.0 Hz. To understand this distinctive discord, we elaborately conducted a series of chiral HPLC separations. To our delight, the results revealed that the isolated substance was a mixture of stereoisomers, which yeilded two pairs of enantiomers **5a/5b** and **6a/6b**, corresponding to the four peaks observed from the chiral HPLC.

The elemental composition of **5 (5a/5b)** was determined as C_32_H_40_O_11_ by HRESIMS [*m*/*z* 623.2456 [M + Na^+^] (calcd 623.2468)]. The IR spectrum exhibited absorption bands of hydroxyl (3367 cm^−1^) and aromatic functionalities (1677, 1598, and 1460 cm^−1^). The ^1^H NMR spectrum showed the presence of a 1,3,4-trisubstituented benzene ring, a symmetrical 1,3,4,5-tetrasubstituted aromatic ring, an asymmetrical 1,3,4,5-tetrasubstituted aromatic ring, three oxymethines, two methylenes, and five methoxyl groups (Table 2). The ^13^C NMR of **5** showed 32 carbon resonances attributable to three benzene rings, five methylenes (three oxygenated), four methines (three oxygenated), and five methoxyl groups (Table 3). The NMR data implied that **5** (**5a**/**5b**) was a sesquineolignan, as a methoxylated derivative of dihydrobuddlenol B^34^. This conclusion was further confirmed by 2D NMR spectra, especially the key HMBC correlations from H-7 to C-1, C-2, and C-6, from H-8 to C-4′, from H-7′ to C-1′, C-2′, C-6′, C-4′′, and C-5′′, and from H-7′′ to C-1′′, C-2′′, and C-6′′. Thus, compound **5** (**5a**/**5b**) was assigned as 3,7,3′,5′,3′′-pentamethoxy-4-hydroxy-8,4′-oxy-7′,4′′-epoxy-8′,5′′-sesquineolignan-9,9′,9′′-triol and was named phyllanglaucin C.

The relative configuration of **5** (**5a**/**5b**) was deduced from analyses of the coupling constants and NOESY experiment, as well as by comparison of its chemical shifts with that of dihydrobuddlenol B^34^. The *threo* form geometry of H-7/H-8 was elucidated by the large coupling constant between H-7 and H-8 (*J*_7,8_ = 6.1 Hz in CDCl_3_, and *J*_7,8_ = 6.3 Hz in acetone-*d*_6_) (Table 2)^35^. The NOESY correlation of H-7′/H-9′, along with the large coupling constant of H-7′/H-8′ (*J*_7′,8′_ = 7.4 Hz in CDCl_3_, and *J*_7′,8′_ = 6.6 Hz in acetone-*d*_6_) indicated a *trans* configuration of H-7′/H-8′ (Table 2). The absolute configurations of **5a** and **5b** were defined by their ECD spectra (Fig. 3), a negative CE at 238 nm (Δ*ε* − 2.06) of **5a** suggested the 8*R* configuration^35^, and the negative ^1^L_b_ band CE at 294 nm (Δ*ε* − 0.65) indicated the 7′*R* configuration^24^. Therefore, the absolute configuration of **5a** was assigned as 7*R*, 8*R*, 7′*R*, 8′*S*. Since compounds **5a** and **5b** showed opposite ECD curves (Fig. 3) and optical rotations (**5a**: 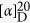 + 7.5; **5b**: 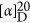 − 7.4), the absolute configuration of **5b** was determined to be 7*S*, 8*S*, 7′*S*, 8′*R*. Finally, compound **5a** was named (+)-phyllanglaucin C and **5b** was named (−)-phyllanglaucin C, respectively.

Compound **6** (**6a/6b**) had the same molecular formula as **5**. The ^1^H and ^13^C NMR data of **6** were very close to those of **5** (Tables 2 and 3), assisted by the 2D NMR spectra of **6**, compounds **5** and **6** elucidated to have the same planar structure and relative configurations. The 8*R* and 7′*S* configurations of **6a** was established on the basis of a negative CE at 230 nm (Δ*ε* − 1.48) and a positive ^1^L_b_ CE at 282 nm (Δ*ε* + 1.41) (Fig. 3)^35^. Therefore, **6a** (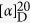 + 14.4) was elucidated as (7*R*,8*R*,7′*S*,8′*R*)-3,7,3′,5′,3′′-pentamethoxy-4-hydroxy-8,4′-oxy-7′,4′′-epoxy-8′,5′′-sesquineolignan-9,9′,9′′-triol and named (+)-phyllanglaucin D. Accordingly, **6b** (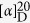 − 13.0) was elucidated to be (7*S*,8*S*,7′*R*,8′*S*)-3,7,3′,5′,3′′-pentamethoxy-4-hydroxy-8,4′-oxy-7′,4′′-epoxy-8′,5′′-sesquineolignan-9,9′,9′′-triol and named (−)-phyllanglaucin D.

Given the same state as **5** and **6**, chiral HPLC resolution of the mixture of **7** and **8** were performed, and afforded two pairs of sesquilignan enantiomers **7a**/**7b** and **8a**/**8b**.

Compound **7** (**7a**/**7b**) was isolated as a white powder, the IR spectrum of **7** showed absorption bands at 3366, 1599 and 1460 cm^−1^ assignable to hydroxyl and aromatic functionalities. The molecular formula was concluded to be C_31_H_38_O_11_ by HRESIMS [*m*/*z* 609.2294 [M + Na^+^] (calcd 609.2312)]. The NMR data of **7** was superimposable on those of acernikol (Tables 2 and 3), whose absolute configuration was ambiguous^36^. The 7,8-*erythro* configuration of **7** was determined by the small coupling constant of H-7 and H-8 (*J*_7,8_ = 5.1 Hz in CD_3_OD, and *J*_7,8_ = 4.8 Hz in acetone-*d*_6_) (Table 2)^37^. The strong NOESY correlation of H-7′/H-8′, and the absence of correlation between H-7′ and H-9′ indicated a *cis* configuration for H-7′ and H-8′.

The ECD spectrum of **7a** showed a positive CE at 244 nm (Δ*ε* + 2.32) indicating an 8*S* configuration^38^, and a negative ^1^L_b_ CE at 294 nm (Δ*ε* − 0.79) suggesting a 7′*R* configuration (Fig. 3)^24^, **7b** owned the opposite ECD curves (Fig. 3) and optical rotations with **7a** (**7a**: 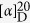 + 22.4; **7b**: 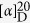 − 20.0). Thus, **7a** was elucidated to be (7*R*,8*S*,7′*R*,8′*R*)-acernikol, and **7b** was elucidated to be (7*S*,8*R*,7′*S*,8′*S*)-acernikol.

Compound **8** (**8a**/**8b**) had the same planar structure and relative configurations as **7** deducing from their closely related 1D and 2D NMR (Tables 2 and 3). **8a** exhibited a positive CE at 243 nm (Δ*ε* + 6.51) and a positive CE at 281 nm (Δ*ε* + 1.76), suggestive an 8*S* configuration^38^ and a 7′*S* configuration^24^. Therefore, **8a** was elucidated as (7*R*,8*S*,7′*S*,8′*S*)-acernikol, and **8b** was determined to be (7*S*,8*R*,7′*R*,8′*R*)-acernikol.

The structures of known compounds **9a**/**9b** and **10**−**12** were characterized as (7*R*,8*S*)-dihydrodehydroconiferyl alcohol (**9a**)^39^, (7*S*,8*R*)-dihydrodehydrodiconiferyl alcohol (**9b**)^40^, (7*S*,8*R*)-cedrusin (**10**)^21^, (7*S*,8*R*)-dihydrodehydrodiconifenyl alcohol 9-*O*-*β*-D-xylopyranoside (**11**)^41^, and (7*S*,8*R*)-4,7,9,9′-tetrahydroxy-3,3′-dimethoxy-8-*O*-4′-neolignan (**12**)^42^, respectively, by comparing their ^1^H and ^13^C NMR data and optical rotations with the reported literature values.

Reactive oxygen species (ROS) plays an important role in the normal physiological processes^43^. Oxidative stress, referring to the imbalance of pro-oxidants and antioxidant defenses, involves a wide variety of pathological processes, including cardiovascular disease^44^, rheumatic disease^45^,^46^, neurodegeneration^47^, tumor^48^, and so on. In addition, it was reported that the same type of lignans showed strong antioxidant activities^49^. In this case, compounds **1a**/**1b**−**8a**/**8b** were evaluated for their antioxidant activities by the DPPH free radical scavenging assay^50^. As showed in Table 4, compound **1b** showed strong activity against DPPH radical with the IC_50_ value of 5.987 ± 1.212 *μ*g/mL, which was comparable with the IC_50_ value of the positive control (IC_50_ = 4.485 ± 0.157 *μ*g/mL) (Table 4). Compounds **2a**/**2b**, **3a**/**3b**, and **4b** also showed moderate antioxidant activity. A comparison of the structures of **7** and **8** with those of **5** and **6** showed that the antioxidant potency of the co-isolated was declined when a hydroxyl group at C-7 was replaced by a methoxy group. This fact implied that the presence of hydroxyl groups were crucial for the antioxidant activity. Comparison of the biological activities of the stereoisomers indicated that the variety of the stereochemistry had little effect on the antioxidant activity.

## Methods

### General experimental procedures

Optical rotations were measured on a Perkin-Elmer PE-341 polarimeter (Perkin-Elmer, Waltham, MA, USA). ECD spectra were measured on a Jasco J-810 spectrometer (Jasco, Easton, MD, USA). UV spectra were taken in a Varian Cary 50 UV/VIS spectrophotometer (Varian, Salt Lake City, UT, USA). IR spectra were recorded with a Bruker Vertex 70 FT-IR spectrophotometer (Bruker, Karlsruhe, Germany). HRESIMS data were obtained on a Thermo Fisher LTQ XL LC/MS (Thermo Fisher, Palo Alto, CA, USA). NMR spectra were recorded on Bruker AM-400 spectrometers and Bruker AM-600 spectrometers (Bruker, Karlsruhe, Germany). Chemical shifts were given in ppm with reference to the residual CD_3_OD (*δ*_H_ 3.31/*δ*_C_ 49.0), CDCl_3_ (*δ*_H_ 7.26/*δ*_C_ 77.16), and CD_3_COCD_3_ (*δ*_H_ 2.05) signals. Silica gel (100–200 mesh and 200–300 mesh was used for column chromatography, Qingdao Marine Chemical Inc., Qingdao, China), ODS (50 *μ*m, YMC, Japan), and Sephadex LH-20 (Pharmacia Biotech AB, Uppsala, Sweden) were used for column chromatography. Semipreparative HPLC was performed on a Dionex Ultimate 3000 HPLC (Dionex, Sunnyvale, CA, USA) with UV detector and an Ultimate XB-C_18_ (10 × 250 mm, 5 *μ*m) column. The chiral HPLC isolation was accomplished on Daicel Chiralpak IA, IC, and ASH columns (4.6 × 250 mm, 5 *μ*m; Daicel Chemical Ltd, Tokyo, Japan). TLC was carried out with glass precoated with silica gel GF_254_ (Qingdao Marine Chemical Inc. China). Solvents were distilled prior to use, and spectroscopic grade solvents were used.

### Plant materials

The whole plants of *P. glaucus* were collected at Lin’an, Zhejiang Province, People’s Republic of China, in August 2012, and identified by Prof. Yunhe He of Zhejiang A&F University. A voucher specimen (No. 20120805A) was deposited in the herbarium of Hubei Key Laboratory of Natural Medicinal Chemistry and Resource Evaluation, Tongji Medical College, Huazhong University of Technology and Science.

### Extraction and isolation

Part I: Air dried whole plant (25.0 kg) of *Phyllanthus glaucus* was chopped and soaked in 70% aqueous acetone at room temperature (3 × 7 d). The acetone extract was evaporated under reduced pressure, the residue was suspended in H_2_O and extracted successively with petroleum ether (60–90 °C), EtOAc, and *n*-BuOH. The EtOAc fraction (250.0 g) was subjected to silica gel CC using a stepwise gradient elution of CH_2_Cl_2_–MeOH (1:0–0:1) to afford four subfractions (A–D). Fraction B (35.0 g) was subjected to MCI gel CC (eluted with MeOH–H_2_O) to remove chlorophyll, and the residue (19.0 g) was partitioned by a RP-C_18_ CC (gradient elution of MeOH–H_2_O, 10:90–100:0) to eight subfractions (B1–B8). Fr. B4 (eluted with MeOH–H_2_O, 40:60) was chromatographed on a Sephadex LH-20 column (MeOH) to afford six subfractions B4-1–B4-6, B4-4 (421 mg) was subjected to silica gel CC (CH_2_Cl_2_–MeOH, from 30:1 to 5:1) to give B4-4-1 to B4-4-3. B4-4-2 was purified by semipreparative HPLC thrice (MeOH–H_2_O, 40:60, MeCN–H_2_O, 32:68, MeCN–H_2_O, 26:74, respectively) to afford **1** (6.7 mg). Fr. B5 (eluted with MeOH–H_2_O, 50:50) was separated by Sephadex LH-20 column (MeOH) to give six subfractions B5-1–B5-6. B5-3 was further separated by silica gel CC (CH_2_Cl_2_–MeOH, from 30:1 to 5:1) to give five subfractions (B5-3-1 to B5-3-5), B5-3-1 (100.8 mg) was subjected to silica gel CC (CH_2_Cl_2_–MeOH, from 40:1 to 5:1) to give B5-3-1-1 to B5-3-1-4, B5-3-1-1 (35 mg) was purified by YMC ODS and normal phase semipreparative column to afford **2** (12.0 mg) and **3** (12.0 mg). Fr. B6 (eluted with MeOH–H_2_O, 60:40) was chromatographed on a Sephadex LH-20 column (MeOH) to give three subfractions B6-1–B6-3. B6-2 was separated by silica gel CC (CH_2_Cl_2_–MeOH, from 40:1 to 5:1) to give six portion (B6-2-1–B6-2-6), B6-2-4 (485 mg) was further purified by silica gel CC (CH_2_Cl_2_–MeOH, 30:1) to afford **7**/**8** (26 mg). Fr. B7 (a mixture eluted with MeOH–H_2_O 70:30 and 80:20) was chromatographed on a Sephadex LH-20 column (MeOH) to give four subfractions (B7-1–B7-4). B7-2 was chromatographed on silica gel column eluting with CH_2_Cl_2_–MeOH (from 40:1 to 3:1) to afford six portion (B7-2-1–B7-2-6). B7-2-3 (217 mg) was purified by semipreparative HPLC twice (MeOH–H_2_O, 56:44, and MeCN–H_2_O, 40:60) to furnish **5**/**6** (25 mg). Fraction C was subjected to MCI gel CC (eluted with MeOH–H_2_O) to remove chlorophyll, and the residue (19.0 g) was partitioned by RP-C_18_ CC (gradient elution of MeOH–H_2_O, 15:85–80:20) to 9 subfractions (C1–C9). Fr. C5 was chromatographed on a Sephadex LH-20 column (MeOH) to give four subfractions (C5-1–C5-4). C5-2 was separated on silica gel CC (CH_2_Cl_2_–MeOH, from 25:1 to 3:1) to give C5-2-1 to C5-2-4. C5-2-4 (530 mg) was further eluted by silica gel CC (CH_2_Cl_2_-MeOH, from 20:1 to 2:1) to give seven subfractions (C5-2-4-1–C5-2-4-7). C5-2-4-2 (88 mg) was purified by semipreparative HPLC (MeCN–H_2_O, 30:70) to yield **4** (9.5 mg).

Part II: Chiral resolution of **1** (6.7 mg) was performed on Daicel chiral-pak IA column (eluted with *n*-hexane–EtOH–HCOOH, v/v/v, 70:30:0.1; flow rate 1.0 mL/min, column temperature 27 °C) to give **1a** (1.7 mg, t_R_ 8.3 min) and **1b** (4.8 mg, t_R_ 11.4 min). **2** (12 mg) was separated by Daicel chiral-pak ASH column (eluted with *n*-hexane–EtOH–HCOOH, v/v/v, 80:20:0.05; flow rate 1.0 mL/min, column temperature 25 °C) to give **2a** (5.3 mg, t_R_ 12.2 min) and **2b** (5.3 mg, t_R_ 22.3 min). **3** (12 mg) was also purified by Daicel chiral-pak ASH column (eluted with *n*-hexane–EtOH–HCOOH, v/v/v, 110:10:0.05; flow rate 1.0 mL/min, column temperature 25 °C) to give **3a** (4.5 mg, t_R_ 17.8 min) and **3b** (4.1 mg, t_R_ 19.4 min). **4** (9.5 mg) was eluted by Daicel chiral-pak ASH column (*n*-hexane–EtOH–HCOOH, v/v/v, 100:20:0.05; flow rate 1.0 mL/min, column temperature 26 °C) to give **4a** (1.5 mg, t_R_ 8.5 min) and **4b** (7.5 mg, t_R_ 15.1 min). **5** and **6** were gained together from one peak on the semipreparative HPLC, and had been divided into four peaks on Daicel chiral-pak ASH column (eluted with *n*-hexane–EtOH–HCOOH, v/v/v, 80:20:0.05, flow rate 1.0 mL/min, column temperature 26 °C). As a result, we got **5a** (4.0 mg, t_R_ 35.9 min), **5b** (6.6 mg, t_R_ 13.3 min), **6a** (8.5 mg, t_R_ 18.4 min), **6b** (4.6 mg, t_R_ 31.5 min). **7** and **8** were obtained together from one peak on the semipreparative HPLC as a mixture named A, A was partitioned to A1 (5.0 mg, t_R_ 17 min) and A2 (6.5 mg, t_R_ 20 min) by Daicel chiral-pak IC column (eluted with *n*-hexane–EtOH–HCOOH, v/v/v, 60:40:0.1; flow rate 1.0 mL/min, column temperature 27 °C), A1 then purified by Daicel chiral-pak ASH column (eluted with *n*-hexane–EtOH–HCOOH, v/v/v, 60:40:0.05; flow rate 1.0 mL/min, column temperature 26 °C) and gave **7a** (2.5 mg, t_R_ 7.7 min) and **8a** (2.0 mg, t_R_ 12.8 min), A2 was further purified on Daicel chiral-pak IA column (eluted with *n*-hexane–EtOH–HCOOH, v/v/v, 70:30:0.05; flow rate 1.0 mL/min, column temperature 27 °C) to furnish **7b** (2.7 mg, t_R_ 12.6 min) and **8b** (2.4 mg, t_R_ 15.6 min).

#### Phyllanglaucin A (**1**)

colorless gum, 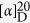 − 6.9 (*c* 0.28, MeOH); UV (MeOH) *λ*_max_ (log *ε*) 211.0 (4.65), 230.5 (4.24), 282.5 (3.62) nm; IR *v*_max_ 3418, 2938, 1614, 1518, 1461, 1380, 1338, 1218, 1115, 1037, 958 cm^−1^; ^1^H and ^13^C NMR data see Table 1; HRESIMS: *m/z* 399.1407 [M + Na]^+^ (calcd for C_20_H_24_O_7_Na, 399.1414).

#### (+)-Phyllanglaucin A (**1a**)


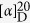
 + 12.8 (*c* 0.065, MeOH); ECD (MeOH) *λ*_max_ (Δ*ε*) 205 (+2.57), 293 (−0.45) nm.

#### (−)-Phyllanglaucin A (**1b**)


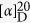
 − 14.1 (*c* 0.19, MeOH); ECD (MeOH) *λ*_max_ (Δ*ε*) 207 (−3.88), 292 (+0.57) nm.

#### 4,4′-dihydroxy-3,7,3′-trimethoxy-8,1′-7′,8′,9′-trinor-neolignan-9-ol (**2**)

colorless gum, 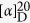 0 (*c* 0.40, MeOH); UV (MeOH) *λ*_max_ (log *ε*) 206.0 (4.47), 228.5 (4.02), 280.5 (3.69) nm; IR *v*_max_ 3366, 2937, 2840, 1606, 1518, 1458, 1432, 1372, 1274, 1220, 1154, 1125, 1096, 1073, 1031 956, 856, 819 cm^−1^; ^1^H and ^13^C NMR data see Table 1; HRESIMS: *m/z* 357.1321 [M + Na]^+^ (calcd for C_21_H_26_O_9_Na, 357.1309).

#### (7R,8R)-4,4′-dihydroxy-3,7,3′-trimethoxy-8,1′-7′,8′,9′-trinor-neolignan-9-ol (**2a**)


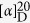
 + 30.0 (*c* 0.18, MeOH); ECD (MeOH) *λ*_max_ (Δ*ε*) 206 (+1.77), 237 (+0.35), 285 (+0.23) nm.

#### (7S,8S)-4,4′-dihydroxy-3,7,3′-trimethoxy-8,1′-7′,8′,9′-trinor-neolignan-9-ol (**2b**)


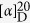
 − 30.8 (*c* 0.22, MeOH); ECD (MeOH) *λ*_max_ (Δ*ε*) 206 (−2.57), 237 (−0.48), 287 (−0.28) nm.

#### 4,4′-dihydroxy-3,7,3′-trimethoxy-8,1′-7′,8′,9′-trinor-neolignan-9-ol (**3**)

colorless gum, 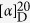 0 (*c* 0.40, MeOH); UV (MeOH) *λ*_max_ (log *ε*) 207.5 (4.47), 230.0 (4.09), 280.5 (3.72) nm; IR *v*_max_ 3353, 2934, 2848, 1605, 1518, 1458, 1432, 1371, 1274, 1218, 1154, 1126, 1099, 1073, 1031 953, 860, 820 cm^−1^; ^1^H and ^13^C NMR data see Table 1; HRESIMS: *m/z* 357.1323 [M + Na]^+^ (calcd for C_21_H_26_O_9_Na, 357.1309).

#### (7S,8R)-4,4′-dihydroxy-3,7,3′-trimethoxy-8,1′-7′,8′,9′-trinor-neolignan-9-ol (**3a**)


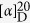
 + 79.5 (*c* 0.13, MeOH); ECD (MeOH) *λ*_max_ (Δ*ε*) 208 (+12.52), 235 (+4.80), 282 (+0.84) nm.

#### (7R,8S)-4,4′-dihydroxy-3,7,3′-trimethoxy-8,1′-7′,8′,9′-trinor-neolignan-9-ol (**3b**)


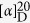
 − 76.9 (*c* 0.11, MeOH); ECD (MeOH) *λ*_max_ (Δ*ε*) 209 (−12.97), 235 (−5.81), 283 (−0.92) nm.

#### Phyllanglaucin B (**4**)

colorless gum, 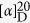 − 42.1 (*c* 0.34, MeOH); UV (MeOH) *λ*_max_ (log *ε*) 206.5 (4.76), 231.5 (4.30), 283.0 (3.92) nm; IR *v*_max_ 3339, 2932, 1654, 1603, 1515, 1496, 1464, 1374, 1271, 1207, 1123, 1066, 1033, 951, 853 cm^−1^; ^1^H and ^13^C NMR data see Table 2 and 3. HRESIMS: *m/z* 561.2089 [M + Na]^+^ (calcd for C_21_H_26_O_9_Na, 561.2101).

#### (+)-Phyllanglaucin B (**4a**)


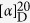
 + 62.6 (*c* 0.06, MeOH); ECD (MeOH) *λ*_max_ (Δ*ε*) 207 (+27.09), 242 (+6.72), 275 (+3.07), 299(+1.29) nm.

#### (−)-Phyllanglaucin B (**4b**)


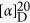
 − 64.8 (*c* 0.25, MeOH); ECD (MeOH) *λ*_max_ (Δ*ε*) 208 (−30.83), 242 (−8.04), 277 (−3.53), 298 (−1.49) nm.

#### Phyllanglaucin C (**5**)

colorless gum, UV (MeOH) *λ*_max_ (log *ε*) 210.0 (4.70), 230.0 (4.27), 280.5 (3.69) nm; IR *v*_max_ 3367, 2939, 2877, 2838, 1677, 1598, 1502, 1460, 1425, 1329, 1277, 1235, 1215, 1125, 1091, 1032, 955, 830 cm^−1^; ^1^H and ^13^C NMR data see Table 2 and 3; HRESIMS: *m/z* 623.2456 [M + Na]^+^ (calcd for C_21_H_26_O_9_Na, 623.2468).

#### (+)-Phyllanglaucin C (**5a**)


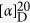
 + 7.5 (*c* 0.13, MeOH); ECD (MeOH) *λ*_max_ (Δ*ε*) 209 (+8.91), 238 (−2.06), 294 (−0.65) nm.

#### (*−*)-Phyllanglaucin C (**5b**)


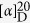
 − 7.4 (*c* 0.24, MeOH); ECD (MeCN) *λ*_max_ (Δ*ε*) 207 (−8.40), 237 (+2.84), 295 (+0.79) nm.

#### Phyllanglaucin D (**6**)

colorless gum, UV (MeOH) *λ*_max_ (log *ε*) 211.0 (4.60), 230.0 (4.22), 280.5 (3.64) nm; IR *v*_max_ 3348, 2937, 2874, 2838, 1598, 1502, 1460, 1424, 1329, 1277, 1235, 1215, 1125, 1090, 1033, 955, 830 cm^−1^; ^1^H and ^13^C NMR data see Table 2 and 3; HRESIMS: *m/z* 623.2461 [M + Na]^+^ (calcd for C_21_H_26_O_9_Na, 623.2468).

#### (+)-Phyllanglaucin D (**6a**)


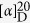
 + 14.4 (*c* 0.24, MeOH); ECD (MeCN) *λ*_max_ (Δ*ε*) 207 (+11.80), 230 (−1.48), 282 (+1.41) nm.

#### (−)-Phyllanglaucin D (**6b**)


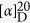
 − 13.0 (*c* 0.18, MeOH); ECD (MeCN) *λ*_max_ (Δ*ε*) 209 (−8.66), 229 (+1.04), 285 (−1.11) nm.

#### acernikol (**7**)

colorless gum, UV (MeOH) *λ*_max_ (log *ε*) 211.0 (4.71), 230.0 (4.28), 280.5 (3.72) nm; IR *v*_max_ 3366, 2937, 2873, 1599, 1501, 1460, 1424, 1382, 1328, 1277, 1236, 1214, 1124, 1031, 953, 831 cm^−1^; ^1^H and ^13^C NMR data see Table 2 and 3; HRESIMS: *m/z* 609.2294 [M + Na]^+^ (calcd for C_21_H_26_O_9_Na, 609.2312).

#### (+)-(7R,8S,7′R,8′R)-acernikol (**7a**)


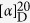
 + 22.4 (*c* 0.11, MeOH); ECD (MeOH) *λ*_max_ (Δ*ε*) 206 (+15.48), 244 (+2.32), 294 (−0.79) nm.

#### (−)-(7S,8R,7′S,8′S)-acernikol (**7b**)


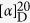
 − 20.0 (*c* 0.12, MeOH); ECD (MeOH) *λ*_max_ (Δ*ε*) 207 (−11.94), 244 (−2.18), 294 (+0.76) nm.

#### acernikol (**8**)

colorless gum, UV (MeOH) *λ*_max_ (log *ε*) 211.5 (4.72), 230.0 (4.33), 281.0 (3.74) nm; IR *v*_max_ 3356, 2923, 2852, 1599, 1501, 1462, 1425, 1382, 1328, 1277, 1236, 1214, 1124, 1030, 954, 830 cm^−1^; ^1^H and ^13^C NMR data see Table 1; HRESIMS: *m/z* 609.2297 [M + Na]^+^ (calcd for C_21_H_26_O_9_Na, 609.2312).

#### (−)-(7R,8S,7′S,8′S)-acernikol (**8a**)


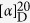
 − 10.2 (*c* 0.13, MeOH); ECD (MeOH) *λ*_max_ (Δ*ε*) 207 (−11.60), 243 (+6.51), 281 (+1.76) nm.

#### (+)-(7S,8R,7′R,8′R)-acernikol (**8b**)


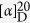
 + 10.7 (*c* 0.11, MeOH); ECD (MeOH) *λ*_max_ (Δ*ε*) 206 (+2.98), 243 (−3.76), 281 (−1.32) nm.

### ECD Calculations

Conformational search using molecular mechanics calculations was performed in Discovery Studio 3.5 Client with MMFF force field with 20 kcal/mol upper energy limit^51^, and nine low energy conformations were chosen as predominant conformers from all the conformations produced by the change of dihedral of the middle four carbons C1-C7-C8-C1′ of 7*R*,8*R*-**2**. The nine predominant conformers were optimized at the B3LYP/6-311++G(2d,p) level in MeOH using the IEFPCM solvation model. The optimized geometries and thermodynamic parameters of all conformations were provided in supplementary information. The calculations were performed using Gaussian 09^52^, and figured using GaussView 5.0^53^.

The theoretical calculation of ECD was performed using time dependent Density Functional Theory (TDDFT) at LC-wPBE/6-311++G(2d,p)//B3LYP/6-311++G(2d,p) level in MeOH with IEFPCM solvation model. The ECD spectra of compound **2a** were obtained by weighting the Boltzmann distribution ratio of each geometric conformation^54^. The ECD spectra were simulated by overlapping Gaussian functions for each transition according to:


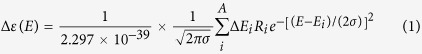


The *σ* represented the width of the band at 1/*e* height, and Δ*E*_*i*_ and *R*_*i*_ were the excitation energies and rotational strengths for transition *i*, respectively.

Additional ECD calculations were performed with two other functionals, i.e., CAM-B3LYP and WB97XD, to further confirm our calculated results.

### DPPH Radical Scavenging Activity

The DPPH radical assay was carried out as previously described with minor modifications^50^. Each sample (final concentrations of 3.125, 6.25, 12.5, 25, and 50 *μ*g/mL) was mixed with DPPH (final concentration of 100 *μ*M) EtOH (with 5% DMSO) solution in 96-well microplate. The mixtures were deposited in the dark at room temperature for 1 h, and then the absorbance was measured at 515 nm using a microplate reader. An EtOH (with 5% DMSO) was used as negative control, and the positive control was Trolox (10 mM, sigma). All tests were performed triplicate, and the results were averaged. The final results were reported as IC_50_, a concentration of a sample that scavenged 50% DPPH free radicals in the reaction solution.

## Additional Information

**How to cite this article**: Wu, Z. *et al.* Enantiomeric Lignans and Neolignans from *Phyllanthus glaucus:* Enantioseparation and Their Absolute Configurations. *Sci. Rep.* **6**, 24809; doi: 10.1038/srep24809 (2016).

## Supplementary Material

Supplementary Information

## Figures and Tables

**Table 1:** ^1^H NMR [400 MHz, *δ* in ppm, Mult. (*J* in Hz)] and ^13^C NMR (100 MHz, *δ* in ppm) Data for 1–3.

*no.*	1^a^		2^b^		3^b^	
	*δ*_H_ (*J*, Hz)	*δ*_C_	*δ*_H_ (*J*, Hz)	*δ*_C_	*δ*_H_ (*J*, Hz)	*δ*_C_
1		129.7		131.9		131.4
2	6.71 s	104.0	6.53 s	109.5	6.58 d (1.8)	109.6
3		149.3		146.4		146.6
4		136.3		144.5		144.7
5		149.3	6.72 d (8.5)	113.9	6.85 d (8.1)	113.9
6	6.71 s	104.0	6.52 overlap	120.7	6.69 dd (8.1, 1.8)	120.9
7	5.51 d (6.0)	88.8	4.31 d (9.3)	89.7	4.39 d (6.6)	85.4
8	3.45 ddd (7.7, 6.0, 5.3)	55.9	3.01 m	55.0	3.07 m	54.6
9a	3.85 dd (10.9, 5.3)	65.2	4.16 dd (11.1, 8.0)	66.9	3.76 overlap	
	64.4					
9b	3.76 dd (10.9, 7.7)	65.2	3.85 dd (11.1, 4.3)	66.9	3.68 m	64.4
1′		136.8		131.3		131.1
2′	6.57 s	117.0	6.31 d (1.9)	111.7	6.57 d (1.8)	112.0
3′		141.9		146.3		146.4
4′		146.5		145.2		145.4
5′		134.4	6.73 d (8.2)	114.3	6.85 d (8.1)	114.3
6′	6.60 s	116.6	6.50 dd (8.2, 1.9)	120.8	6.67 dd (8.1, 1.8)	121.6
7′	2.56 t (7.6)	32.7				
8′	1.79 m	35.8				
9′	3.56 t (6.5)	62.3				
CH_3_O-3	3.81 s	56.7	3.75 s	56.1	3.77 s	56.0
CH_3_O-5	3.81 s	56.7				
CH_3_O-7			3.24 s	56.8	3.16 s	57.1
CH_3_O-3′			3.69 s	56.0	3.81 s	56.0

^a^In CD_3_OD; ^b^In CDCl_3_.

**Table 2:** ^1^H NMR Data for 4–8 and acernikol.

*no.*	4	5		6		7		8		acernikol
	CD_3_OD	CDCl_3_	Me_2_CO-*d*_6_	CDCl_3_	Me_2_CO-*d*_6_	CD_3_OD	Me_2_CO-*d*_6_	CD_3_OD	Me_2_CO-*d*_6_	CD_3_OD
2	6.57 d (1.8)	6.88 d (1.5)	6.97 d (1.2)	6.88 d (1.4)	6.97 d (1.2)	6.97 d (1.7)	7.03 d (1.5)	6.96 d (1.7)	7.03 d (1.6)	6.96 d (2.0)
5	6.59 d (8.2)	6.86 d (8.1)	6.86 d (8.1)	6.86 d (8.2)	66.86 d (8.1)	6.74 d (8.2)	6.77 d (8.1)	6.74 d (8.2)	6.76 d (8.1)	6.74 d (8.0)
6	6.42 dd (8.2,1.8)	6.84 dd (8.1,1.5)	6.81 dd (8.2,1.2)	6.84 dd (8.2,1.4)	6.81 dd (8.2,1.2)	6.78 dd (8.2,1.7)	6.83 dd (8.1,1.5)	6.78 dd (8.2,1.7)	6.83 dd (8.1,1.6)	6.77 dd (8.0,2.0)
7	5.46 d (2.1)	4.60 d (6.1)	4.57 d (6.3)	4.61 d (6.1)	4.57 d (6.3)	4.90 d (5.1)	4.43 d (4.2)	4.90 overlap	4.38 d (4.2)	4.89 d (4.9)
8	2.66 m	4.05 m	4.14 m	4.05 m	4.14 m	4.24 m	4.17 m	4.24 m	4.17 m	4.23 m
9a	3.83 m	3.97 m	3.89 m	3.97 m	3.89 m	3.90 overlap	3.91 m	3.90 dd (6.9,5.4)	3.91 m	3.90 m
9b	3.46 overlap	3.62 m	3.46 m	3.62 m	3.46 m	3.59 overlap	3.55 m	3.60 overlap	3.54 m	3.58 m
2′	6.38 brs	6.61 s	6.73 s	6.61 s	6.73 s	6.71 s	6.74 s	6.71 s	6.74 s	6.70 d (2.0)
6′	6.62 brs	6.61 s	6.73 s	6.61 s	6.73 s	6.71 s	6.74 s	6.71 s	6.74 s	6.70 d (2.0)
7′	4.05 d (8.8)	5.52 d (7.4)	5.55 d (6.6)	5.53 d (7.3)	5.55 d (6.6)	5.55 d (6.0)	5.59 d (6.5)	5.55 d (5.8)	5.58 d (6.5)	5.54 d (6.1)
8′	1.72 m	3.57 m	3.46 m	3.57 m	3.46 m	3.46 m	3.47 m	3.46 m	3.43 m	3.45 m
9′a	3.67 m	3.94 m	3.81 m	3.95 m	3.81 m	3.90 overlap	3.86 overlap	3.86 overlap	3.86 overlap	3.81 m
9′b	3.51 dd (11.2,3.3)	3.91 m	3.57 m	3.92 m	3.57 m	3.76 m	3.79 m	3.75 m	3.79 m	3.75 m
2′′		6.67 d (2.3)	6.77 s	6.67 brd (2.8)	6.77 s	6.75 brs	6.84 s	6.75 brs	6.83 s	6.75 d (2.0)
3′′	6.62 s									
6′′	6.68 s	6.67 d (2.3)	6.77 s	6.67 brd (2.8)	6.77 s	6.72 brs	6.84 s	6.72 brs	6.83 s	6.73 d (2.0)
7′′	2.77 m	2.67 t (7.6)	2.61 t (7.5)	2.67 t (7.6)	2.61 t (7.5)	2.63 t (7.5)	2.61 m	2.63 t (7.6)	2.62 m	2.62 t (7.3)
8′′	1.88 m	1.88 m	1.78 m	1.88 m	1.78 m	1.82 m	1.79 m	1.82 m	1.78 m	1.81 m
9′′	3.67 m	3.69 t (6.3)	3.54 m	3.69 t (6.3)	3.54 m	3.57 t (6.5)	3.55 m	3.57 t (6.5)	3.54 m	3.56 m
CH_3_O-3	3.72 s	3.88 s	3.84 s	3.88 s	3.84 s	3.82s	3.82 s	3.80 s	3.82 s	3.80 s
CH_3_O-7		3.32 s	3.23 s	3.32 s	3.24 s					
CH_3_O-3′	3.46 s	3.73 s	3.77 s	3.73 s	3.77 s	3.79 s	3.84 s	3.79 s	3.84 s	3.77 s
CH_3_O-5′		3.73 s	3.77 s	3.73 s	3.77 s	3.79 s	3.84 s	3.79 s	3.84 s	3.77 s
CH_3_O-3′′		3.88 s	3.84 s	3.88 s	3.84 s	3.87 s	3.86 s	3.88 s	3.86 s	3.87 s
CH_3_O-5′′	3.85 s									

[400 MHz, *δ* in ppm, Mult. (*J* in Hz)].

**Table 3:** ^13^C NMR Data for compounds 4–8 and acernikol.

*no.*	4^a^	5^b^	6^b^	7^a^	8^a^	acernikol^a^
1	134.9	131.1	131.1	133.8	133.8	133.8
2	110.2	109.9	109.9	111.4	111.4	111.5
3	148.8	146.6	146.6	148.7	148.6	148.7
4	146.9	145.2	145.2	146.8	146.7	146.7
5	115.7	114.0	114.0	115.7	115.7	115.7
6	118.9	120.9	120.9	120.7	120.7	120.7
7	87.5	82.6	82.6	74.1	74.0	74.1
8	54.2	86.2	86.2	87.4	87.2	87.4
9	63.3	60.3	60.3	61.6	61.6	61.7
1′	138.2	137.4	137.4	139.7	139.6	139.8
2′	112.8	103.4	103.4	104.0	103.8	104.0
3′	149.0	153.4	153.4	154.6	154.5	154.7
4′	147.9	135.5	135.5	136.3	136.2	136.3
5′	128.3	153.4	153.4	154.6	154.5	154.7
6′	122.9	103.4	103.4	104.0	103.8	104.0
7′	45.5	87.8	87.8	88.6	88.6	88.6
8′	49.3	54.1	54.1	55.7	55.7	55.8
9′	61.4	64.0	64.0	65.1	65.0	65.1
1′′	131.9	135.8	135.8	137.2	137.2	137.2
2′′	130.4	112.6	112.6	114.2	114.1	114.3
3′′	115.6	144.4	144.4	145.3	145.2	145.3
4′′	145.8	146.5	146.5	147.5	147.3	147.5
5′′	144.2	127.6	127.6	129.5	129.4	129.6
6′′	113.6	116.1	116.1	118.0	118.0	118.0
7′′	34.5	32.2	32.2	32.9	32.8	32.9
8′′	39.6	34.7	34.7	35.8	35.7	35.8
9′′	65.9	62.4	62.4	62.2	62.2	62.3
CH_3_O-3	56.4	56.1	56.1	56.4	56.4	56.4
CH_3_O-7		57.4	57.4			
CH_3_O-3′	56.0	56.2	56.2	56.6	56.6	56.7
CH_3_O-5′		56.2	56.2	56.6	56.6	56.7
CH_3_O-3′′		56.1	56.1	56.8	56.8	56.9
CH_3_O-5′′	56.6					

(100 MHz, *δ* in ppm). ^a^In CD_3_OD; ^b^In CDCl_3_.

**Table 4:** Antioxidant Activities of compounds 1−8 (DPPH Test).

Compounds	Removaleffect (%)^a^	IC_50_(*μ*g/mL)	Compounds	Removaleffect (%)^a^	IC_50_(*μ*g/mL)
**1a**	81.149	(−)^b^	**5a**	34.069	(−)^b^
**1b**	86.989	5.987 ± 1.212	**5b**	34.299	(−)^b^
**2a**	81.747	16.622 ± 0.797	**6a**	33.103	(−)^b^
**2b**	78.069	17.941 ± 1.994	**6b**	37.471	(−)^b^
**3a**	77.287	13.306 ± 0.907	**7a**	49.563	(−)^b^
**3b**	81.609	9.641 ± 0.865	**7b**	56.736	(−)^b^
**4a**	57.609	(−)^b^	**8a**	51.540	(−)^b^
**4b**	66.943	26.784 ± 0.812	**8b**	48.598	(−)^b^
			**Trolox**	95.494^c^	4.485 ± 0.157

^a^Concentration of removal effect: 50 μg/mL; ^b^IC_50_ value not determined; ^c^Concentration of removal effect: 25 μg/mL.

## References

[b1] Suzuki S. & Umezawa T. Biosynthesis of lignans and norlignans. J. Wood Sci. 53, 273–284 (2007).

[b2] Pan J. Y. *et al.* An update on lignans: natural products and synthesis. Nat. Prod. Rep. 26, 1251–1292 (2009). 19779640 10.1039/b910940d

[b3] Pereira A. C. *et al.* Schistosomicidal and trypanocidal structure-activity relationships for (±)-licarin A and its (−)-and (+)-enantiomers. Phytochemistry. 72, 1424–1430 (2011). 21570099 10.1016/j.phytochem.2011.04.007

[b4] Silva R. D. *et al.* Trypanocidal structure-activity relationship for *cis*- and *trans*-methylpluviatolide. Phytochemistry. 69, 1890–1894 (2008). 18479721 10.1016/j.phytochem.2008.04.002

[b5] Lai Y. J. *et al.* Neolignans with a rare 2-oxaspiro[4.5]deca-6,9-dien-8-one motif from the stem bark of *Cinnamomum subavenium*. J. Nat. Prod. 78, 1740–1744 (2015). 26087384 10.1021/np5010533

[b6] Shi Y. S. *et al.* Chiral resolution and absolute configuration of a pair of rare racemic spirodienone sesquineolignans from *Xanthium sibiricum*. Org. Lett. 16, 5406–5409 (2014). 25275854 10.1021/ol502649a

[b7] Cheng Z. B. *et al.* (±)-Torreyunlignans A–D, rare 8–9′ linked neolignan enantiomers as phosphodiesterase-9A inhibitors from *Torreya yunnanensis*. J. Nat. Prod. 77, 2651–2657 (2014). 25495612 10.1021/np500528u

[b8] Yang D. T. *et al.* (+)- and (−)-liriodenol, a pair of novel enantiomeric lignans from *Liriodendron hybrid*. Bioorg. Med. Chem. Lett. 25, 1976–1978 (2015). 25817591 10.1016/j.bmcl.2015.03.015

[b9] Qi W. Y., Hua L. & Gao K. Chemical constituents of the plants from the genus *Phyllanthus*. Chem. Biodivers. 11, 364–395 (2014). 24634068 10.1002/cbdv.201200244

[b10] Lv J. J. *et al.* Stereochemistry of cleistanthane diterpenoid glucosides from *Phyllanthus emblica*. RSC. Adv. 5, 29098–29107 (2015).

[b11] Fan Y. Y. *et al.* Phainanoids A–F, A new class of potent immunosuppressive triterpenoids with an unprecedented carbon skeleton from *Phyllanthus hainanensis*. J. Am. Chem. Soc. 137, 138–141 (2014). 25522036 10.1021/ja511813g

[b12] Lv J. J. *et al.* Anti-hepatitis B virus norbisabolane sesquiterpenoids from *Phyllanthus acidus* and the establishment of their absolute configurations using theoretical calculations. J. Org. Chem. 79, 5432–5447 (2014). 24824117 10.1021/jo5004604

[b13] Zhao J. Q. *et al.* Phyllanflexoid C: first example of phenylacetylene-bearing 18-nor-diterpenoid glycoside from the roots of *Phyllanthus flexuosus*. Tetrahedron Lett. 54, 4670–4674 (2013).

[b14] Ren Y. L. *et al.* Potent cytotoxic arylnaphthalene lignan lactones from *Phyllanthus poilanei*. J. Nat. Prod. 77, 1494–1504 (2014). 24937209 10.1021/np5002785 PMC4073661

[b15] Thanh N. V. *et al.* A new flavone sulfonic acid from *Phyllanthus urinaria*. Phytochem. Lett. 7, 182–185 (2014).

[b16] Mensah J. L., Gleye J., Moulis C. & Fouraste I. Alkaloids from the leaves of *Phyllanthus discoideus*. J. Nat. Prod. 51, 1113–1115 (1988).

[b17] Zhang Y. J., Abe T., Tanaka T., Yang C. R. & Kouno I. Phyllanemblinins A–F, new ellagitannins from *Phyllanthus emblica*. J. Nat. Prod. 64, 1527–1532 (2001). 11754604 10.1021/np010370g

[b18] Hu Z. X. *et al.* Phytochemical and chemotaxonomic studies on *Phyllanthus urinaria*. Biochem. Syst. Ecol. 56, 60–64 (2014).

[b19] Zhu H. C. *et al.* Bioactive acylphloroglucinols with adamantyl skeleton from *Hypericum sampsonii*. Org. Lett. 16, 6322–6325 (2014). 25453445 10.1021/ol5030579

[b20] Lai Y. J. *et al.* Scapiformolactones A–I: germacrane sesquiterpenoids with an unusual Δ^3^-15,6-lactone moiety from *Salvia scapiformis*. Phytochemistry. 96, 378–388 (2013). 24189346 10.1016/j.phytochem.2013.10.003

[b21] Yu S. *et al.* New cytotoxic lignan glycosides from *Phyllanthus glaucus*. Nat. Prod. Res. 30, 419–425 (2016). 25791206 10.1080/14786419.2015.1023198

[b22] Agrawal P. K., Agarwal S. K. & Rastogi R. P. A new neolignan and other phenolic constituents from *Cedrus deodara*. Phytochemistry. 19, 1260–1261 (1980).

[b23] Liu Q. B. *et al.* Antioxidant and anti-inflammatory active dihydrobenzofuran neolignans from the seeds of *Prunus tomentosa*. J. Agric. Food Chem. 62, 7796–7803 (2014). 25019337 10.1021/jf502171z

[b24] Antus S. *et al.* Chiroptical properties of 2,3-dihydrobenzo[b]furan and chromane chromophores in naturally occurring O-heterocycles. Chirality. 13, 493–506 (2001). 11466774 10.1002/chir.1067

[b25] Hsiao J. J. & Chiang H. C. Lignans from the wood of *Aralia bipinnata*. Phytochemistry. 39, 899–902 (1995).

[b26] Zhu J. X. *et al.* Phenylpropanoids and lignanoids from *Euonymus acanthocarpus*. *Arch*. Pharm. Res. 35, 1739–1747 (2012). 10.1007/s12272-012-1005-y 23139124

[b27] Zhou C. C. *et al.* Two new compounds from *Crataegus pinnatifida* and their antithrombotic activities. J. Asian Nat. Products Res. 16, 169–174 (2014). 10.1080/10286020.2013.848429 24161196

[b28] Zeng Q. *et al.* Chemical constituents from *Metasequoia glyptostroboides* Hu *et* Cheng. Biochem. Syst. Ecol. 50, 406–410 (2013).

[b29] Liu Y. B. *et al.* Chemical constituents of *Toona ciliata* var. *pubescens*. *Chin*. J. Nat. Med. 9, 115–119 (2011).

[b30] Rayanil K., Nimnoun C. & Tuntiwachwuttikul P. New phenolics from the wood of *Casearia grewiifolia*. Phytochem. Lett. 5, 59–62 (2012).

[b31] Yoshikawa K., Mimura N. & Arihara S. Isolation and absolute structures of enantiomeric 1,2-*bis*(4-hydroxy-3-methoxyphenyl)-1,3-propanediol 1-*O*-glucosides from the bark of *Hovenia trichocarpa*. J. Nat. Prod. 61, 1137–1139 (1998). 9748383 10.1021/np980003d

[b32] Wu W., Bi X. L., Cao J. Q., Zhang K. Q. & Zhao Y. Q. New antitumor compounds from *Carya cathayensis*. Bioorg. Med. Chem. Lett. 22, 1895–1898 (2012). 22330636 10.1016/j.bmcl.2012.01.062

[b33] Crabbé P. & Klyne W. Optical rotatory dispersion and circular dichroism or aromatic compounds: a general survey. Tetrahedron. 23, 3449–3503 (1967).

[b34] Yoshinari K., Shimazaki N., Sashida Y. & Mimaki Y. Flavanone xyloside and lignans from *Prunus jamasakura* bark. Phytochemistry. 29, 1675–1678 (1990).

[b35] Xiong L. *et al.* Lignans and neolignans from *Sinocalamus affinis* and their absolute configurations. J. Nat. Prod. 74, 1188–1200 (2011). 21469695 10.1021/np200117y

[b36] Morikawa T., Tao J., Ueda K., Matsuda H. & Yoshikawa M. Medicinal foodstuffs. XXXI. Structures of new aromatic constituents and inhibitors of degranulation in RBL-2H3 cells from a Japanese folk medicine, the stem bark of *Acer nikoense*. Chem. Pharm. Bull. 51, 62–67 (2003). 12520130 10.1248/cpb.51.62

[b37] Huo C. H., Liang H., Zhao Y. Y., Wang B. & Zhang Q. Y. Neolignan glycosides from *Symplocos caudata*. Phytochemistry. 69, 788–795 (2008). 17905350 10.1016/j.phytochem.2007.08.022

[b38] Fang L. *et al.* Neolignans and glycosides from the stem bark of *Illicium difengpi*. J. Nat. Prod. 73, 818–824 (2010). 20411974 10.1021/np900712v

[b39] Hanawa F., Shiro M. & Hayashi Y. Heartwood constituents of *Betula maximowicziana*. Phytochemistry. 45, 589–595 (1997).

[b40] Shi S. P., Jiang D., Dong C. X. & Tu P. F. Lignans from the roots and rhizomes of *Clematis manshurica*. Zeitschrift für Naturforschung B. 61, 1299–1303 (2006).

[b41] Liu J. F. *et al.* Two new lignans and anti-HBV constituents from *Illicium henryi*. Chem. Biodivers. 8, 692–698 (2011). 21480515 10.1002/cbdv.201000110

[b42] Liu Q. B. *et al.* Neolignans from the seeds of *Prunus tomentosa* (Rosaceae) and their chemotaxonomic interest. Biochem. Syst. Ecol. 55, 236–240 (2014).

[b43] Auten R. L. & Davis J. M. Oxygen toxicity and reactive oxygen species: the devil is in the details. Pediatr. Res. 66, 121–127 (2009). 19390491 10.1203/PDR.0b013e3181a9eafb

[b44] Coombes J. S. & Fassett R. G. Antioxidant therapy in hemodialysis patients: a systematic review. Kidney Int. 81, 233–246 (2012). 21975860 10.1038/ki.2011.341

[b45] Perl A. Oxidative stress in the pathology and treatment of systemic lupus erythematosus. Nat. Rev. Rheumatol. 9, 674–686 (2013). 24100461 10.1038/nrrheum.2013.147 PMC4046645

[b46] Schiller J., Fuchs B., Arnhold J. & Arnold K. Contribution of reactive oxygen species to cartilage degradation in rheumatic diseases: molecular pathways, diagnosis and potential therapeutic strategies. Curr. Med. Chem. 10, 2123–2145 (2003). 12871089 10.2174/0929867033456828

[b47] Miller M. W. & Sadeh N. Traumatic stress, oxidative stress and post-traumatic stress disorder: neurodegeneration and the accelerated-aging hypothesis. Mol. Psychiatr. 19, 1156–1162 (2014). 10.1038/mp.2014.111 PMC4211971 25245500

[b48] Gorrini C., Harris I. S. & Mak T. W. Modulation of oxidative stress as an anticancer strategy. Nat. Rev. Drug Discov. 12, 931–947 (2013). 24287781 10.1038/nrd4002

[b49] Saleem M., Kim H. J., Ali M. S. & Lee Y. S. An update on bioactive plant lignans. Nat. Prod. Rep. 22, 696–716 (2005). 16311631 10.1039/b514045p

[b50] Krishnaiah D., Sarbatly R. & Nithyanandam R. A review of the antioxidant potential of medicinal plant species. Food Bioprod. Process. 89, 217–233 (2011).

[b51] Smith S. G. & Goodman J. M. Assigning stereochemistry to single diastereoisomers by GIAO NMR calculation: The DP4 probability. J. Am.Chem. Soc. 132, 12946–12959 (2010). 20795713 10.1021/ja105035r

[b52] Frisch M. J. *et al.* Gaussian 09. Gaussian Inc. : Wallingford, CT, 2010.

[b53] Dennington R., Keith T. & Millam J. GaussView, Version 5.0. Semichem Inc.: Shawnee Mission, KS, 2009

[b54] Tähtinen P., Bagno A., Klika K. D. & Pihlaja K. Modeling NMR parameters by DFT methods as an aid to the conformational analysis of cis-fused 7a(8a)-methyl octa(hexa)hydrocyclopenta [*d]*[1,3] oxazines and [3,1] benzoxazines. J. Am. Chem. Soc. 125, 4609–4618 (2003). 12683833 10.1021/ja021237t

