# Supplementary material for: Enantiomeric Lignans and Neolignans from Phyllanthus glaucus: Enantioseparation and Their Absolute Configurations
Source: Sci Rep. 2016 Apr 29;6:24809. doi: 10.1038/srep24809 (PMC4850383; doi:10.1038/srep24809)

Enantiomeric Lignans and Neolignans from *Phyllanthus glaucus*: Enantioseparation and Their Absolute Configurations

Zhaodi Wu,[1,*] Yongji Lai,[2,*] Lei Zhou,[1] Ye Wu,[1] Hucheng Zhu,[1] Zhengxi Hu,[1] Jing Yang,[3] Jinwen Zhang,[4] Jianping Wang,[1] Zengwei Luo,[1] Yongbo Xue,[1] and Yonghui Zhang[1]

[1]Hubei Key Laboratory of Natural Medicinal Chemistry and Resource Evaluation, School of Pharmacy, Tongji Medical College, Huazhong University of Science and Technology, Wuhan 430030, Hubei Province, People's Republic of China.

[2]Department of Pharmacy, the Central Hospital of Wuhan, Wuhan 430014, Hubei Province, People's Republic of China.

[3]State Key Laboratory of Phytochemistry and Plant Resources in West China, Kunming Institute of Botany, Chinese Academy of Sciences, Kunming 650204, People's Republic of China.

[4]Tongji Hospital Affiliated to Tongji Medical College,Huazhong University of Science and Technology,Wuhan 430030, People's Republic of China.

[*]These authors contributed equally to the work. Correspondence and requests for materials should be addressed to Y. Z. (zhangyh@mails.tjmu.edu.cn) or Y. X. (yongboxue@mail.hust.edu.cn)

CONTENTS

ECD Calculations for compound 7*R*,8*R*-**2** at LC-wPBE/6-311++G(2d,p)//B3LYP/6-311++G(2d,p) level..3

ECD Calculations for compound 7*R*,8*R*-**2** at CAM-B3LYP/6-311++G(2d,p)//B3LYP/6-311++G(2d,p) level................................12

ECD Calculations for compound 7*R*,8*R*-**2** at WB97XD/6-311++G(2d,p)//B3LYP/6-311++G(2d,p) level 14

HRESIMS, UV, IR, and NMRspectra of **1** 17

HRESIMS, UV, IR, and NMRspectra of **2** 21

HRESIMS, UV, IR, and NMRspectra of **3** 26

HRESIMS, UV, IR, and NMRspectra of **4** 30

HRESIMS, UV, IR, and NMRspectra of **5** 35

HRESIMS, UV, IR, and NMRspectra of **6** 40

HRESIMS, UV, IR, and NMRspectra of **7** 45

HRESIMS, UV, IR, and NMRspectra of **8** 51

Chiral HPLC separation profiles of **1a**/**1b**−**8a**/**8b** 56

ECD Calculation

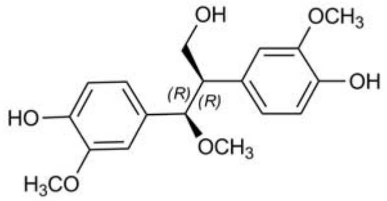

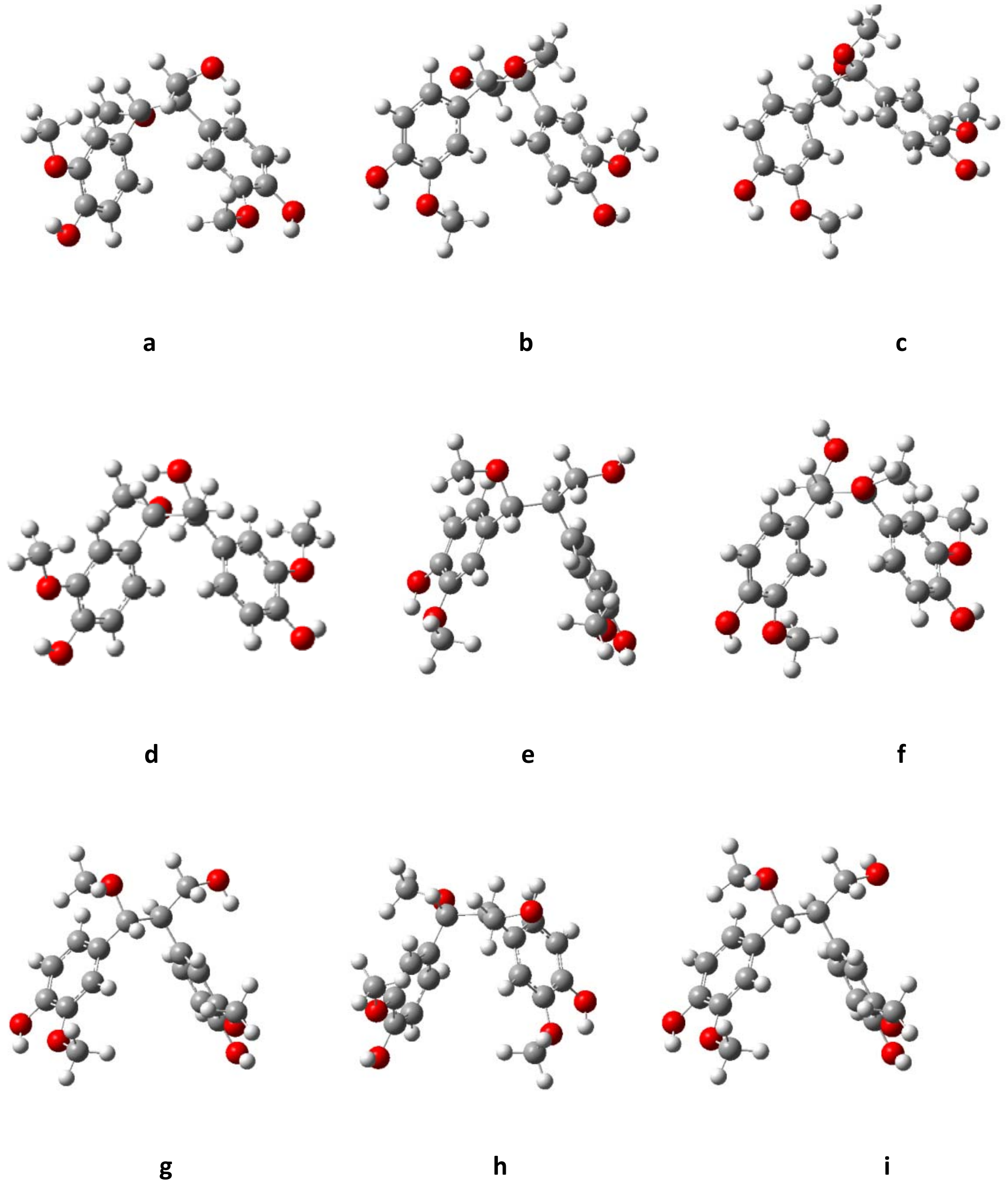

Figure S1. Optimized geometries of predominant conformers for compound 7*R*,8*R*-**2** at the B3LYP/6-311++G(2d,p) level in MeOH using the IEFPCM solvation model.

Table S1. Important thermodynamic parameters (a.u.) and Boltzmann distributions of the optimized compound 7*R*,8*R*-**2** calculated at LC-wPBE/6-311++G(2d,p)//B3LYP/6-311++G(2d,p) level in MeOH with IEFPCM solvation model.

Conformations	E+ZPE	G	%
**a**	-1149.747078	-1150.513587	1.6
**b**	-1149.745583	-1150.512212	0.33
**c**	-1149.746992	-1150.513539	1.46
**d**	-1149.745532	-1150.512444	0.31
**e**	-1149.749154	-1150.516256	14.42
**f**	-1149.747124	-1150.513755	1.68
**g**	-1149.750597	-1150.517638	66.49
**h**	-1149.745795	-1150.512478	0.41
**i**	-1149.749077	-1150.516285	13.29

E is the total electronic energy in MeOH at LC-wPBE/6-311++G(2d,p)//B3LYP/6-311++G(2d,p) level with IEFPCM solvation model, *ZPE* is the zero point energy in MeOH at B3LYP/6-311++G(2d,p) level with IEFPCM solvation model, and *G* is the Gibbs free energy by the sum of *E* and the thermal correction obtained at B3LYP/6-311++G(2d,p) level. %: Boltzmann distributions, using the relative Gibbs free energies as weighting factors.

Table S2. Optimized coordinates of 7*R*,8*R*-**2** in MeOH (Å) at B3LYP/6-311++G(2d,p) level using IEFPCM solvation model.

**a**				**b**			
C	1.695498	1.24566	-1.781071	C	1.675083	1.305759	-1.746384
C	2.80427	1.541562	-1.006386	C	2.799466	1.576151	-0.985033
C	3.191554	0.662256	0.017627	C	3.203664	0.666481	0.005268
C	2.4616	-0.496755	0.245978	C	2.473705	-0.496082	0.214971
C	1.328604	-0.790433	-0.523855	C	1.32455	-0.763933	-0.540362
C	0.959606	0.087402	-1.537985	C	0.93971	0.143408	-1.522205
O	3.524426	2.678883	-1.246672	O	3.518926	2.717797	-1.206834
C	0.538304	-2.058741	-0.242261	C	0.534214	-2.036236	-0.277196
C	-2.901844	-1.447399	-0.28392	C	-2.885122	-1.45242	-0.306374
C	-1.748658	-0.96044	0.327432	C	-1.748502	-0.9609	0.330532
C	-0.651544	-1.922162	0.753013	C	-0.639859	-1.915356	0.74061
C	-3.928084	-0.591632	-0.680905	C	-3.919885	-0.604401	-0.700271
C	-3.818266	0.770033	-0.460269	C	-3.832285	0.75403	-0.453785
C	-2.669767	1.279674	0.165758	C	-2.698068	1.267027	0.194735
C	-1.648883	0.421643	0.552194	C	-1.670985	0.416639	0.578957

O	-1.188004	-1.762871	3.152069	O	-1.172235	-1.866062	3.081661
C	-0.147378	-1.652868	2.177396	C	-0.103245	-1.664014	2.149827
O	-4.826409	1.611198	-0.842955	O	-4.848235	1.589682	-0.833245
O	0.000273	-2.634262	-1.431116	O	-0.024363	-2.581589	-1.470112
C	0.976848	-3.246757	-2.26403	C	0.937241	-3.17661	-2.332145
O	4.308728	1.055428	0.703297	O	4.336188	1.036132	0.679125
C	4.780716	0.227515	1.767643	C	4.828909	0.17445	1.706484
O	-2.680308	2.638176	0.333267	O	-2.729601	2.623488	0.3878
C	-1.579351	3.252129	1.005812	C	-1.647044	3.237786	1.088065
H	1.413473	1.927916	-2.574125	H	1.380463	2.011205	-2.514184
H	2.774711	-1.186513	1.019148	H	2.799177	-1.208825	0.961765
H	0.098804	-0.136018	-2.153888	H	0.066306	-0.059185	-2.127321
H	4.259803	2.706777	-0.616429	H	4.266546	2.725404	-0.590605
H	1.227237	-2.785606	0.210731	H	1.227221	-2.77587	0.1471
H	-3.005869	-2.511568	-0.457369	H	-2.972127	-2.514467	-0.501148
H	-1.095843	-2.921461	0.771855	H	-1.075356	-2.918901	0.758298
H	-4.821886	-0.976854	-1.156849	H	-4.802113	-0.994001	-1.194105
H	-0.765368	0.829455	1.021838	H	-0.803412	0.827273	1.074522
H	-1.876158	-1.122731	2.928632	H	-0.841772	-1.672538	3.966455
H	0.337469	-0.67477	2.253365	H	0.300684	-0.65257	2.251537
H	0.590632	-2.407539	2.453459	H	0.708723	-2.369094	2.358388
H	-4.571194	2.51388	-0.600522	H	-4.608613	2.489812	-0.567007
H	1.710025	-2.521781	-2.63083	H	1.665777	-2.444726	-2.694552
H	1.503806	-4.044585	-1.727355	H	1.471499	-3.987086	-1.822196
H	0.444604	-3.676055	-3.111933	H	0.390591	-3.58605	-3.180737
H	5.656689	0.730078	2.16965	H	5.715498	0.661803	2.103954
H	4.023345	0.128323	2.548467	H	4.08902	0.053329	2.500874
H	5.062468	-0.760723	1.397231	H	5.098697	-0.802695	1.299525
H	-1.482868	2.867876	2.023887	H	-1.559028	2.834334	2.09955
H	-0.648044	3.090289	0.458974	H	-0.705656	3.099553	0.551863
H	-1.804842	4.315082	1.035915	H	-1.88684	4.297028	1.13669
**c**				**d**			
C	1.482045	1.444303	-1.779465	C	-1.814898	1.961531	1.156591
C	2.61233	1.751249	-1.040989	C	-3.091679	1.880552	0.625962
C	3.077911	0.845913	-0.074167	C	-3.537934	0.671507	0.070664
C	2.402482	-0.349053	0.135928	C	-2.695271	-0.432899	0.054333
C	1.249196	-0.656349	-0.598228	C	-1.395356	-0.350043	0.569379
C	0.802477	0.248071	-1.556298	C	-0.97124	0.853668	1.125458
O	3.277639	2.925974	-1.262788	O	-3.916255	2.970855	0.657971
C	0.528827	-1.968754	-0.33779	C	-0.489591	-1.569161	0.525943
C	-2.932596	-1.545927	-0.161481	C	1.234458	0.722924	-1.380693
C	-1.772852	-1.013139	0.396848	C	1.510597	-0.460556	-0.6983
C	-0.605433	-1.924753	0.729364	C	0.533718	-1.621861	-0.645454

C	-4.027578	-0.738356	-0.465717	C	2.16453	1.761543	-1.426083
C	-3.979584	0.619743	-0.205784	C	3.3836	1.632797	-0.785289
C	-2.823523	1.174402	0.364673	C	3.680513	0.447301	-0.094246
C	-1.734712	0.364897	0.658944	C	2.753061	-0.584245	-0.059296
O	0.86483	-2.692208	2.479039	O	-0.74318	-3.16034	-2.098067
C	-0.061565	-1.653573	2.13332	C	-0.148423	-1.858705	-2.003098
O	-5.054766	1.414324	-0.498479	O	4.295413	2.652471	-0.830905
O	-0.059104	-2.508191	-1.521471	O	0.270685	-1.720426	1.725441
C	0.886007	-3.062899	-2.426572	C	-0.503677	-2.121108	2.848489
O	4.20925	1.254118	0.580033	O	-4.821276	0.709685	-0.402898
C	4.764463	0.395386	1.577546	C	-5.374277	-0.479705	-0.968892
O	-2.898106	2.525393	0.578511	O	4.917485	0.434569	0.493245
C	-1.792733	3.180332	1.203006	C	5.312512	-0.728522	1.22248
H	1.139774	2.147519	-2.529448	H	-1.488057	2.897976	1.592726
H	2.769557	-1.058335	0.866303	H	-3.055443	-1.373256	-0.342085
H	-0.075781	0.016041	-2.143743	H	0.021884	0.928795	1.547143
H	4.039185	2.956589	-0.664705	H	-4.761379	2.72024	0.255924
H	1.266055	-2.685179	0.041281	H	-1.126662	-2.457084	0.41513
H	-2.988643	-2.60831	-0.36525	H	0.28597	0.859056	-1.883427
H	-0.994641	-2.946965	0.744718	H	1.116703	-2.526217	-0.447795
H	-4.926402	-1.159319	-0.900263	H	1.946101	2.679169	-1.959194
H	-0.848223	0.808687	1.087286	H	2.983273	-1.499347	0.468355
H	1.204353	-2.512774	3.363344	H	-1.666755	-3.113186	-1.830982
H	-0.898517	-1.644195	2.838515	H	0.611986	-1.81596	-2.783921
H	0.435991	-0.680561	2.185433	H	-0.892331	-1.089847	-2.224654
H	-4.834501	2.32143	-0.239216	H	5.077226	2.376508	-0.329742
H	1.581435	-2.305443	-2.802024	H	-1.255887	-1.370714	3.111264
H	1.460084	-3.866351	-1.949856	H	-1.007016	-3.076269	2.656564
H	0.321527	-3.473799	-3.262957	H	0.18674	-2.241546	3.682478
H	5.64055	0.912068	1.961069	H	-6.383909	-0.220926	-1.277361
H	4.051641	0.228982	2.38832	H	-4.79635	-0.802989	-1.837674
H	5.062798	-0.562326	1.145116	H	-5.411037	-1.282382	-0.228816
H	-1.608489	2.768805	2.19811	H	4.634903	-0.912503	2.059216
H	-0.890759	3.092658	0.593476	H	5.342805	-1.605343	0.571636
H	-2.075912	4.226533	1.286901	H	6.31039	-0.518781	1.599246
**e**				**f**			
C	-3.226225	0.228304	-1.582693	C	1.478937	1.436255	-1.787765
C	-3.438877	-0.961361	-0.904963	C	2.601564	1.753347	-1.041755
C	-2.700184	-1.243489	0.254175	C	3.064449	0.855481	-0.066775
C	-1.75976	-0.329518	0.713971	C	2.394205	-0.342455	0.14372
C	-1.535678	0.86784	0.027364	C	1.248236	-0.659605	-0.597581
C	-2.278713	1.137271	-1.118617	C	0.80417	0.237592	-1.563716
O	-4.370536	-1.851313	-1.363201	O	3.261888	2.930601	-1.264171

C	-0.506299	1.862986	0.53823	C	0.532967	-1.974742	-0.336953
C	1.431881	0.103043	-1.901527	C	-2.932082	-1.532141	-0.184364
C	1.41745	0.682955	-0.637934	C	-1.771254	-1.011772	0.383663
C	0.716498	2.011235	-0.402394	C	-0.6115	-1.932285	0.71704
C	2.072937	-1.115129	-2.130922	C	-4.020216	-0.714729	-0.486863
C	2.710457	-1.767974	-1.092062	C	-3.964151	0.640817	-0.215327
C	2.708811	-1.195317	0.190776	C	-2.807063	1.182886	0.365248
C	2.069202	0.016425	0.4101	C	-1.725194	0.36369	0.658203
O	2.719263	3.271031	-0.842012	O	0.88493	-2.648795	2.560216
C	1.674293	3.084865	0.123072	C	-0.076049	-1.677173	2.135471
O	3.340968	-2.962376	-1.31336	O	-5.032426	1.445061	-0.506363
O	-1.072072	3.169273	0.692443	O	-0.036821	-2.526178	-1.524193
C	-2.002324	3.270922	1.762667	C	0.923679	-3.073785	-2.41764
O	-3.006332	-2.442435	0.838728	O	4.188187	1.272994	0.594168
C	-2.319435	-2.810522	2.036066	C	4.73806	0.424063	1.603105
O	3.373585	-1.933081	1.133119	O	-2.87384	2.532332	0.590164
C	3.448878	-1.427943	2.466971	C	-1.765171	3.175688	1.220851
H	-3.807894	0.434766	-2.47318	H	1.13859	2.133738	-2.543935
H	-1.1918	-0.540564	1.610363	H	2.761492	-1.045917	0.879564
H	-2.130407	2.066491	-1.654822	H	-0.068035	-0.002109	-2.157116
H	-4.379814	-2.611695	-0.762837	H	4.018494	2.968683	-0.660218
H	-0.154168	1.520319	1.520616	H	1.271127	-2.683735	0.055837
H	0.937265	0.601807	-2.726498	H	-2.994105	-2.592325	-0.397932
H	0.338147	2.363072	-1.366653	H	-1.010865	-2.952111	0.714966
H	2.082337	-1.562067	-3.117845	H	-4.919634	-1.125905	-0.929474
H	2.078732	0.448831	1.401484	H	-0.838211	0.79838	1.094873
H	3.26441	4.011704	-0.553225	H	0.44122	-3.50155	2.645084
H	2.099892	2.778622	1.086117	H	-0.919634	-1.647135	2.831832
H	1.129156	4.017094	0.272437	H	0.439184	-0.718641	2.198512
H	3.727984	-3.255741	-0.475109	H	-4.806872	2.348762	-0.239718
H	-2.875675	2.630783	1.603221	H	1.614525	-2.310088	-2.788635
H	-1.533698	3.000859	2.716449	H	1.501628	-3.869083	-1.931983
H	-2.32412	4.310795	1.804077	H	0.37228	-3.493912	-3.25812
H	-2.718869	-3.780174	2.32206	H	5.607637	0.948028	1.991517
H	-1.244959	-2.893784	1.858847	H	4.018121	0.25945	2.407878
H	-2.50868	-2.086183	2.831578	H	5.045764	-0.534989	1.180312
H	3.962201	-0.464015	2.490005	H	-1.586114	2.757242	2.213989
H	2.452322	-1.326918	2.902876	H	-0.862539	3.084878	0.612757
H	4.021165	-2.160345	3.030629	H	-2.040933	4.223421	1.310061
**g**				**h**			
C	-3.23784	0.216766	-1.596305	C	-1.677508	-1.281765	-1.765657
C	-3.447139	-0.976161	-0.923252	C	-2.790594	-1.564816	-0.992367
C	-2.71113	-1.258665	0.237543	C	-3.184236	-0.668065	0.013866

C	-1.776718	-0.341741	0.703656	C	-2.455825	0.494932	0.226711
C	-1.556115	0.859137	0.021855	C	-1.31836	0.77571	-0.541452
C	-2.296282	1.128728	-1.126068	C	-0.943297	-0.119369	-1.538196
O	-4.372851	-1.86894	-1.387667	O	-3.509049	-2.706372	-1.217411
C	-0.530866	1.85514	0.538364	C	-0.529664	2.048447	-0.276382
C	1.487092	0.143143	-1.907369	C	2.892602	1.431779	-0.311843
C	1.415922	0.697353	-0.633052	C	1.748675	0.955919	0.323804
C	0.691578	2.013266	-0.398138	C	0.652499	1.923761	0.732175
C	2.158855	-1.058178	-2.136895	C	3.917243	0.569938	-0.702517
C	2.773223	-1.718633	-1.088951	C	3.811551	-0.786651	-0.453726
C	2.719927	-1.170049	0.204068	C	2.669714	-1.284064	0.19413
C	2.050069	0.0249	0.424367	C	1.653063	-0.420142	0.575495
O	2.744235	3.336478	-0.755888	O	1.142399	1.779927	3.152041
C	1.650945	3.092025	0.133263	C	0.122579	1.691029	2.154656
O	3.432666	-2.89524	-1.310833	O	4.816949	-1.635997	-0.830149
O	-1.095461	3.161467	0.695046	O	0.017888	2.603707	-1.470245
C	-2.029159	3.260878	1.762629	C	-0.952087	3.202754	-2.320322
O	-3.013745	-2.460825	0.817097	O	-4.305743	-1.049702	0.699143
C	-2.32999	-2.829387	2.016111	C	-4.786075	-0.20268	1.744464
O	3.368158	-1.9123	1.152234	O	2.68412	-2.640233	0.389453
C	3.399857	-1.428246	2.496255	C	1.591903	-3.240361	1.087248
H	-3.817498	0.423426	-2.488045	H	-1.390641	-1.977501	-2.545157
H	-1.211541	-0.552774	1.601818	H	-2.773944	1.197935	0.985774
H	-2.150519	2.060408	-1.658717	H	-0.07867	0.093373	-2.152425
H	-4.380894	-2.631256	-0.789731	H	-4.247735	-2.724096	-0.590692
H	-0.180257	1.510126	1.520482	H	-1.22154	2.783533	0.158087
H	1.010131	0.648168	-2.738839	H	2.99312	2.492251	-0.509113
H	0.315726	2.362769	-1.364058	H	1.096131	2.925489	0.728603
H	2.210432	-1.485837	-3.130906	H	4.805016	0.947194	-1.195966
H	2.017086	0.438049	1.423392	H	0.779794	-0.818728	1.070854
H	3.206334	2.500843	-0.902414	H	1.511305	2.671444	3.134834
H	2.024536	2.813465	1.126155	H	-0.306118	0.695212	2.26548
H	1.126989	4.039768	0.226481	H	-0.671768	2.414873	2.367128
H	3.798399	-3.198257	-0.466327	H	4.565013	-2.532657	-0.563479
H	-2.902174	2.621494	1.598539	H	-1.682096	2.471863	-2.681649
H	-1.563633	2.988132	2.717133	H	-1.483677	4.008597	-1.800362
H	-2.350553	4.300773	1.805654	H	-0.413249	3.619099	-3.17054
H	-2.725554	-3.802071	2.29712	H	-5.665033	-0.698102	2.148837
H	-1.254353	-2.906437	1.843199	H	-4.034846	-0.088931	2.529227
H	-2.526455	-2.108744	2.813224	H	-5.065191	0.778516	1.353852
H	3.898243	-0.457724	2.548376	H	1.507027	-2.835847	2.098531
H	2.390563	-1.349278	2.906281	H	0.653754	-3.089666	0.548804
H	3.96761	-2.161826	3.062774	H	1.817726	-4.30264	1.136211

i							
C	-3.18743	0.20692	-1.618757				
C	-3.414513	-0.974373	-0.931051				
C	-2.699755	-1.242461	0.24625				
C	-1.76795	-0.32351	0.713565				
C	-1.529184	0.865352	0.017191				
C	-2.249156	1.121231	-1.14653				
O	-4.337584	-1.869287	-1.396853				
C	-0.511828	1.866839	0.538333				
C	1.470763	0.137388	-1.899389				
C	1.427944	0.690563	-0.62434				
C	0.730747	2.017167	-0.376939				
C	2.113266	-1.078048	-2.139687				
C	2.724177	-1.754962	-1.100374				
C	2.694647	-1.209276	0.193952				
C	2.054111	-0.000087	0.423894				
O	2.810534	3.333608	-0.664967				
C	1.6895	3.0803	0.189211				
O	3.355737	-2.946693	-1.332062				
O	-1.084547	3.173117	0.669472				
C	-2.027439	3.284905	1.727523				
O	-3.01941	-2.433327	0.840008				
C	-2.358846	-2.785628	2.056789				
O	3.335088	-1.968932	1.135442				
C	3.389316	-1.487877	2.479299				
H	-3.751127	0.402902	-2.523061				
H	-1.219274	-0.523499	1.624403				
H	-2.090197	2.044215	-1.690393				
H	-4.359961	-2.621918	-0.787141				
H	-0.177557	1.533351	1.530225				
H	0.996234	0.654523	-2.724935				
H	0.366102	2.386444	-1.341441				
H	2.144421	-1.503983	-3.135404				
H	2.042042	0.410368	1.424474				
H	2.491345	3.766093	-1.466063				
H	2.114586	2.743107	1.136499				
H	1.140903	4.00353	0.377597				
H	3.723001	-3.25864	-0.491622				
H	-2.894079	2.635487	1.568838				
H	-1.567475	3.034032	2.690692				
H	-2.357635	4.322759	1.748448				
H	-2.765514	-3.750893	2.347376				
H	-1.280904	-2.872263	1.903935				

H	-2.564307	-2.050279	2.838058				
H	3.907403	-0.527497	2.528403				
H	2.38579	-1.388883	2.899327				
H	3.947449	-2.23349	3.039807				

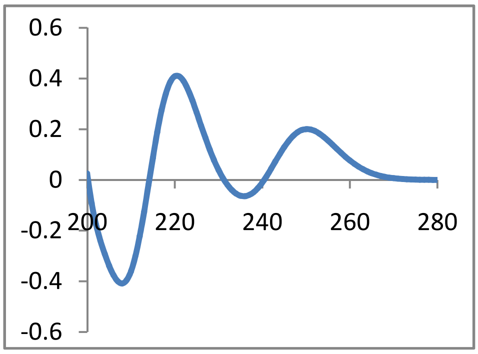

(a)

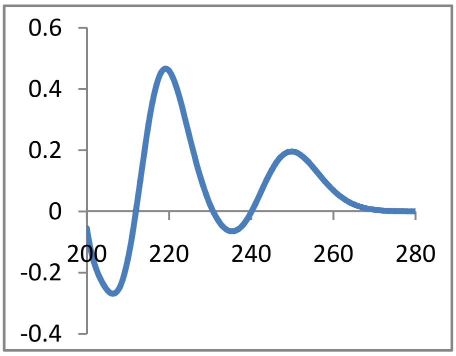

(b)

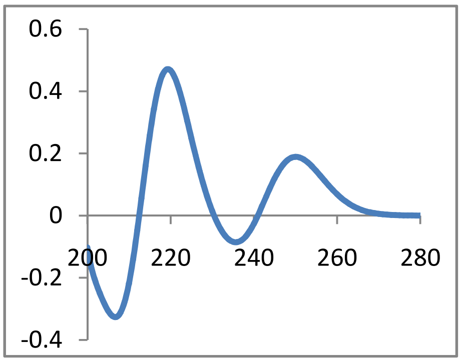

(c)

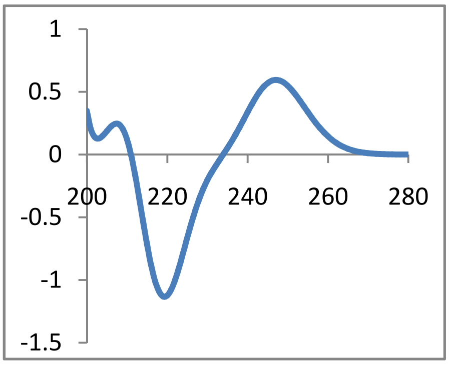

(d)

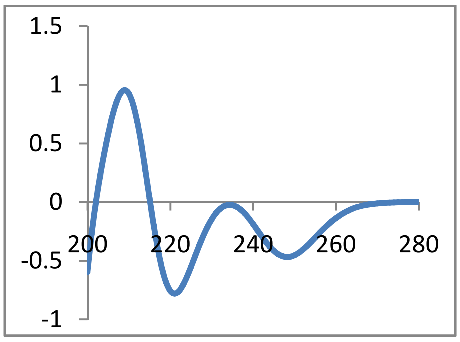

(e)

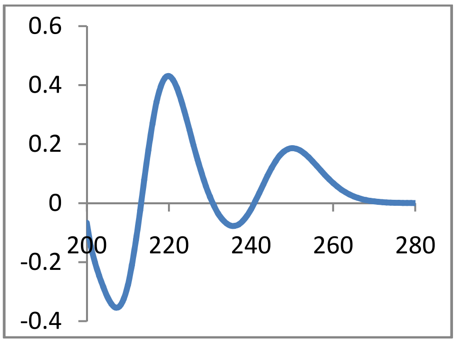

(f)

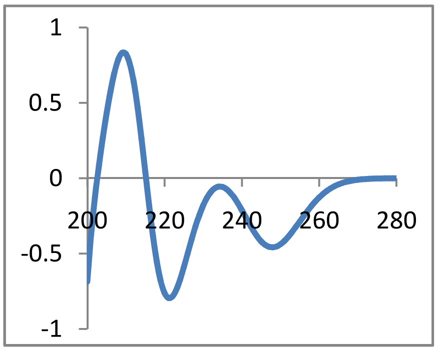

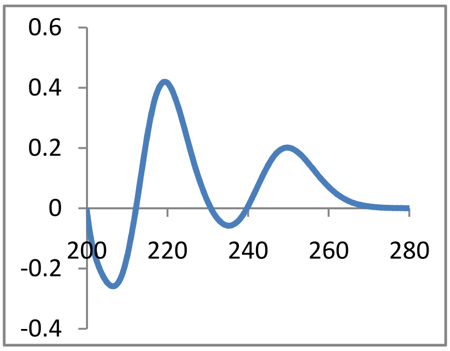

(g) (h)

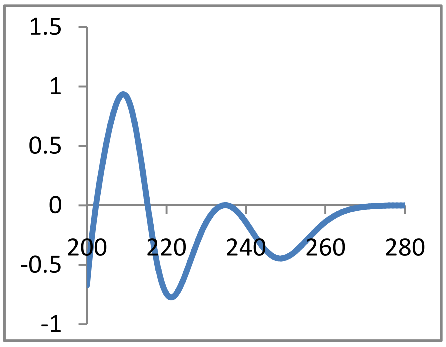

(i)

Figure S2. Calculated ECDs of each conformations of 7*R*,8*R*-**2** (calculated at the LC-wPBE/6-311++G(2d,p)//B3LYP/6-311++G(2d,p) level in MeOH with IEFPCM solvation model.

Table S3. Important thermodynamic parameters (a.u.) and Boltzmann distributions of the optimized compound 7*R*,8*R*-**2** calculated at CAM-B3LYP/6-311++G(2d,p)//B3LYP/6-311++G(2d,p) level in MeOH with IEFPCM solvation model.

Conformations	E+ZPE	G	%
**a**	-1149.973171	-1150.513587	1.6
**b**	-1149.971854	-1150.512212	0.33
**c**	-1149.973336	-1150.513539	1.46
**d**	-1149.971887	-1150.512444	0.31
**e**	-1149.975695	-1150.516256	14.42
**f**	-1149.973400	-1150.513755	1.68
**g**	-1149.976998	-1150.517638	66.49
**h**	-1149.972007	-1150.512478	0.41
**i**	-1149.975586	-1150.516285	13.29

E is the total electronic energy in MeOH at CAM-B3LYP/6-311++G(2d,p)//B3LYP/6-311++G(2d,p) level with IEFPCM solvation model, *ZPE* is the zero point energy in MeOH at B3LYP/6-311++G(2d,p) level with IEFPCM solvation model, and *G* is the Gibbs free energy by the sum of *E* and the thermal correction obtained at B3LYP/6-311++G(2d,p) level. %: Boltzmann distributions, using the relative Gibbs free energies as weighting factors.

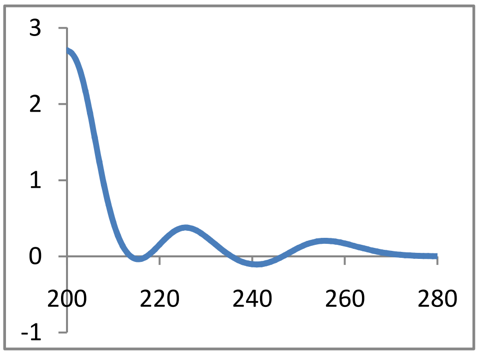

(a)

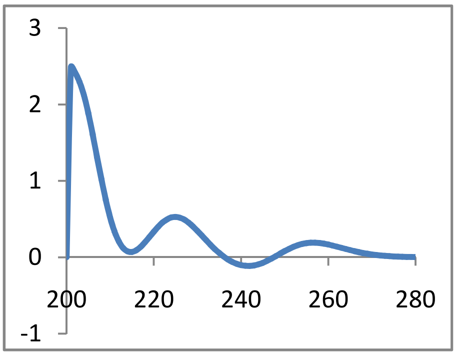

(b)

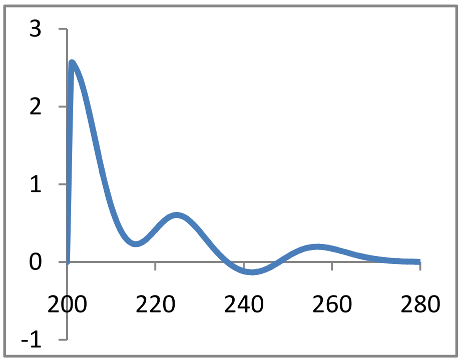

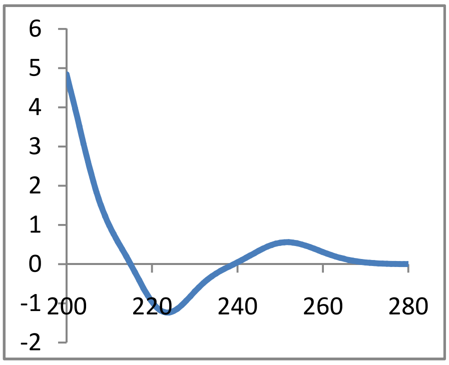

(c) (d)

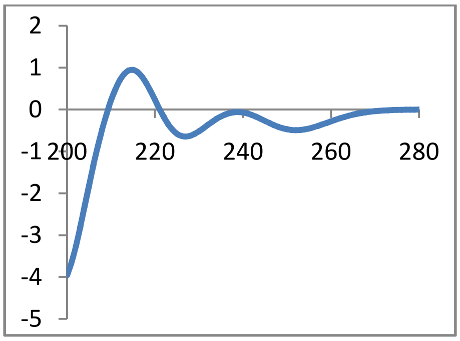

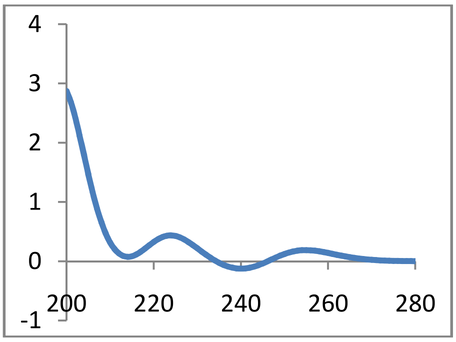

(e) (f)

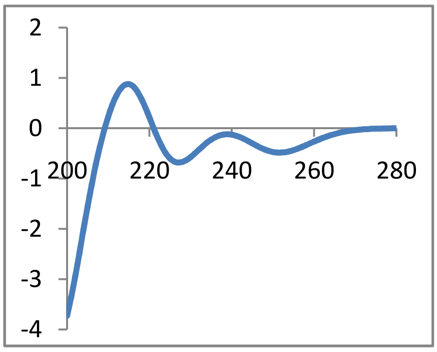

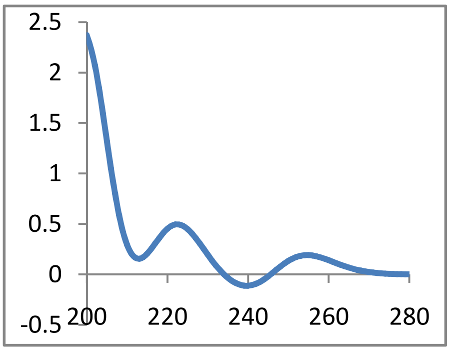

(g) (h)

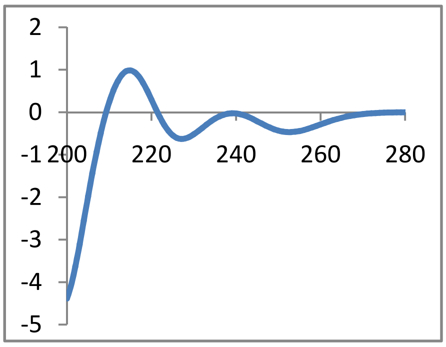

(i)

Figure S3. Calculated ECDs of each conformations of 7*R*,8*R*-**2** (calculated at the

CAM-B3LYP/6-311++G(2d,p)//B3LYP/6-311++G(2d,p) level in MeOH with IEFPCM solvation model.

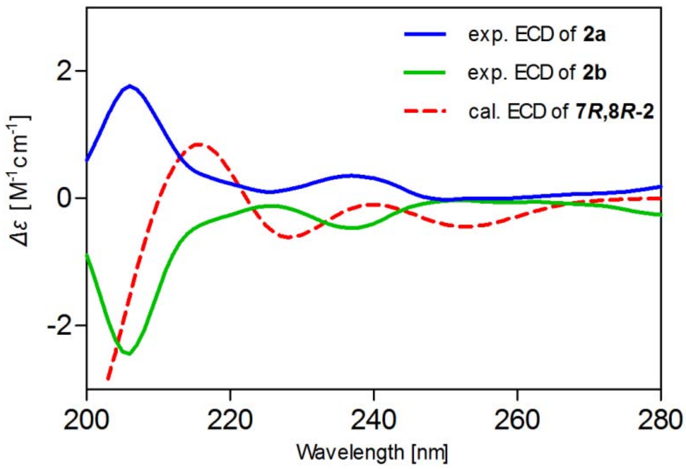

Figure S4. Calculated and experimental ECDs of **2** and 7*R*,8*R*-**2** at the CAM-B3LYP/6-311++G(2d,p)//B3LYP/6-311++G(2d,p) level in MeOH with IEFPCM solvation model.

Table S4. Important thermodynamic parameters (a.u.) and Boltzmann distributions of the optimized compound 7*R*,8*R*-**2** calculated at WB97XD/6-311++G(2d,p)//B3LYP/6-311++G(2d,p) level in MeOH with IEFPCM solvation model.

Conformations	E+ZPE	G	%
**a**	-1150.142381	-1150.513587	5.79
**b**	-1150.140407	-1150.512212	0.72
**c**	-1150.141879	-1150.513539	3.40
**d**	-1150.140170	-1150.512444	0.56
**e**	-1150.142802	-1150.516256	9.04
**f**	-1150.142219	-1150.513755	4.87
**g**	-1150.144647	-1150.517638	63.72
**h**	-1150.140881	-1150.512478	1.18
**i**	-1150.142965	-1150.516285	10.73

E is the total electronic energy in MeOH at WB97XD/6-311++G(2d,p)//B3LYP/6-311++G(2d,p) level with IEFPCM solvation model, *ZPE* is the zero point energy in MeOH at B3LYP/6-311++G(2d,p) level with IEFPCM solvation model, and *G* is the Gibbs free energy by the sum of *E* and the thermal correction obtained at B3LYP/6-311++G(2d,p) level. %: Boltzmann

distributions, using the relative Gibbs free energies as weighting factors.

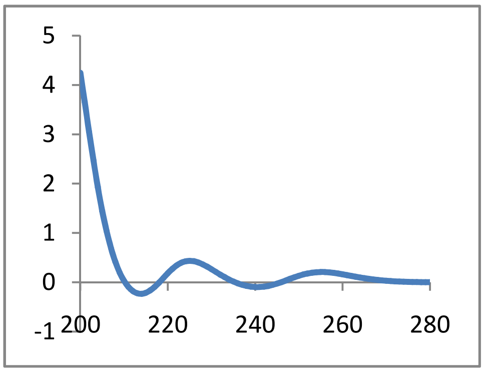

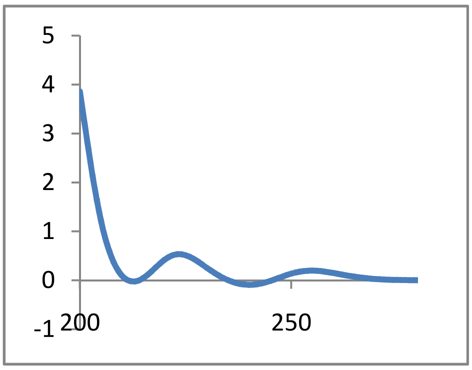

(a)(b)

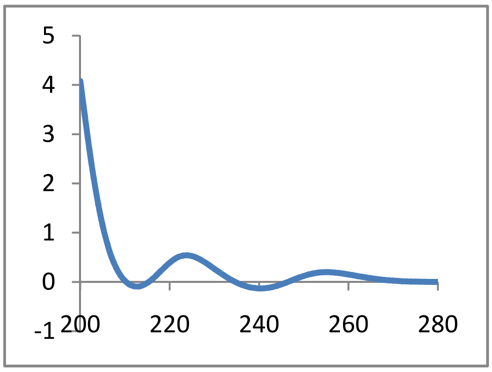

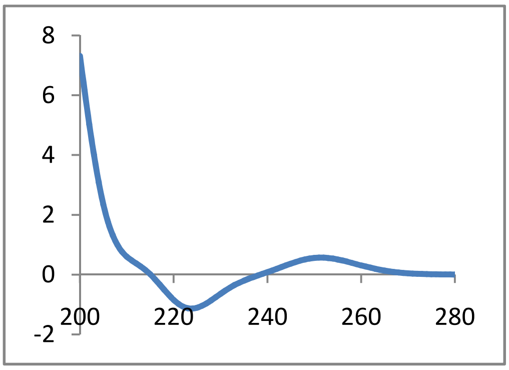

(c) (d)

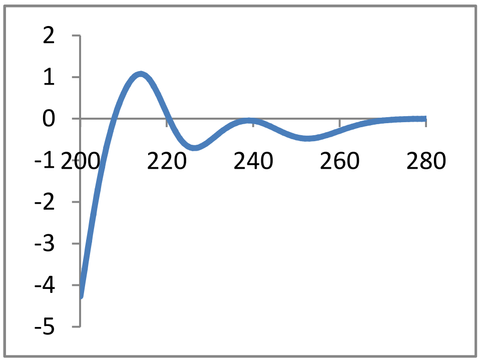

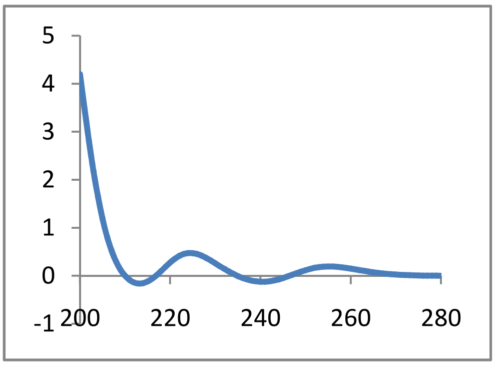

(e) (f)

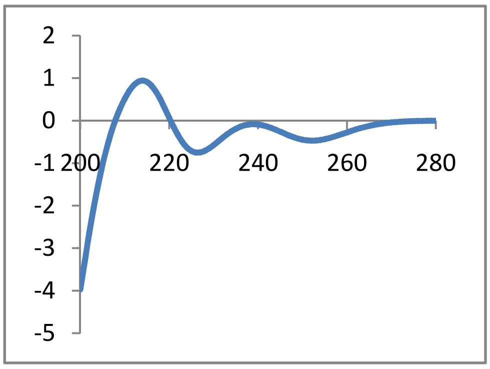

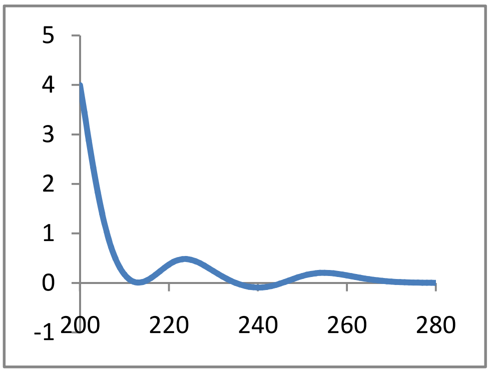

(g) (h)

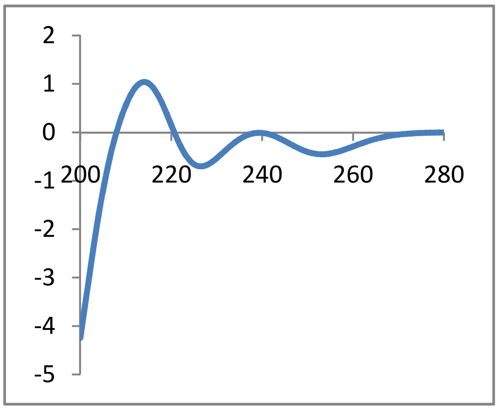

(i)

Figure S5. Calculated ECDs of each conformations of 7*R*,8*R*-**2** (calculated at the WB97XD/6-311++G(2d,p)//B3LYP/6-311++G(2d,p) level in MeOH with IEFPCM solvation model.

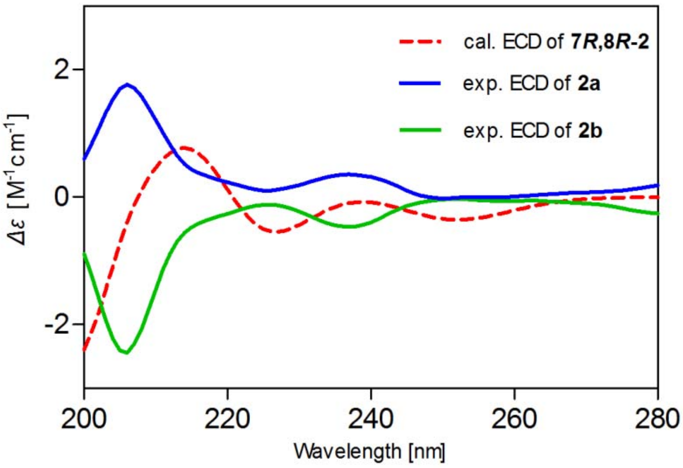

FigureS6. Calculated and experimental ECDs of **2** and 7*R*,8*R*-**2** at the WB97XD/6-311++G(2d,p)//B3LYP/6-311++G(2d,p) level in MeOH with IEFPCM solvation model.

HRESIMS of compound 1

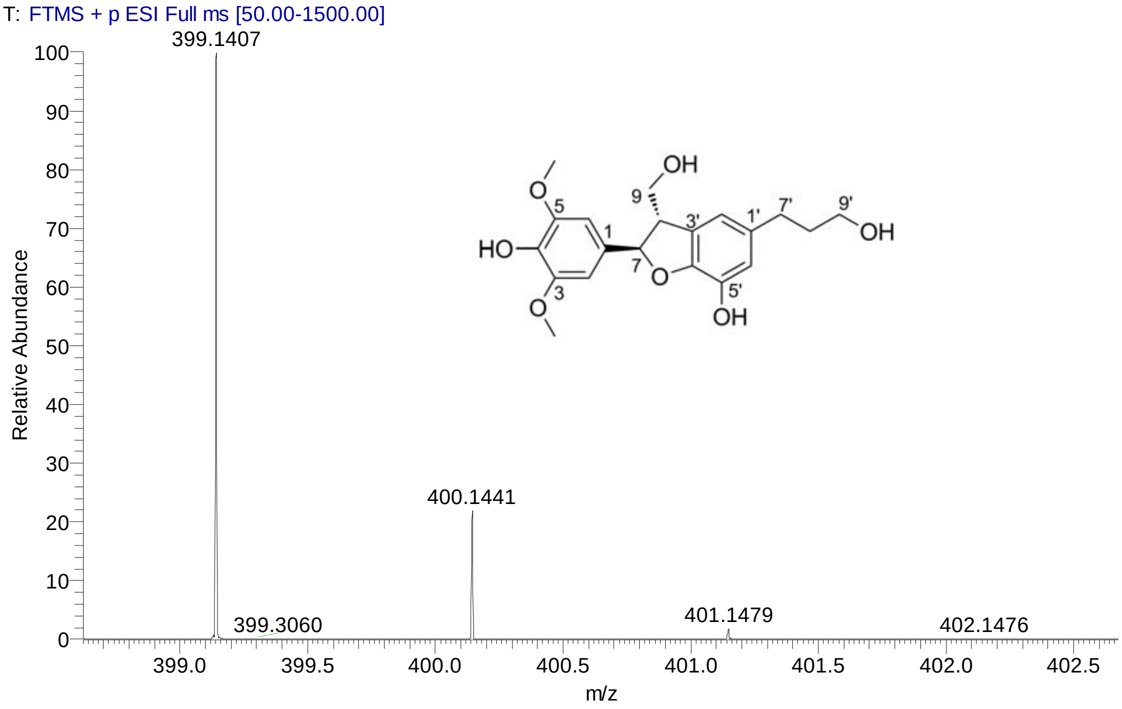

UV spectrum of compound 1

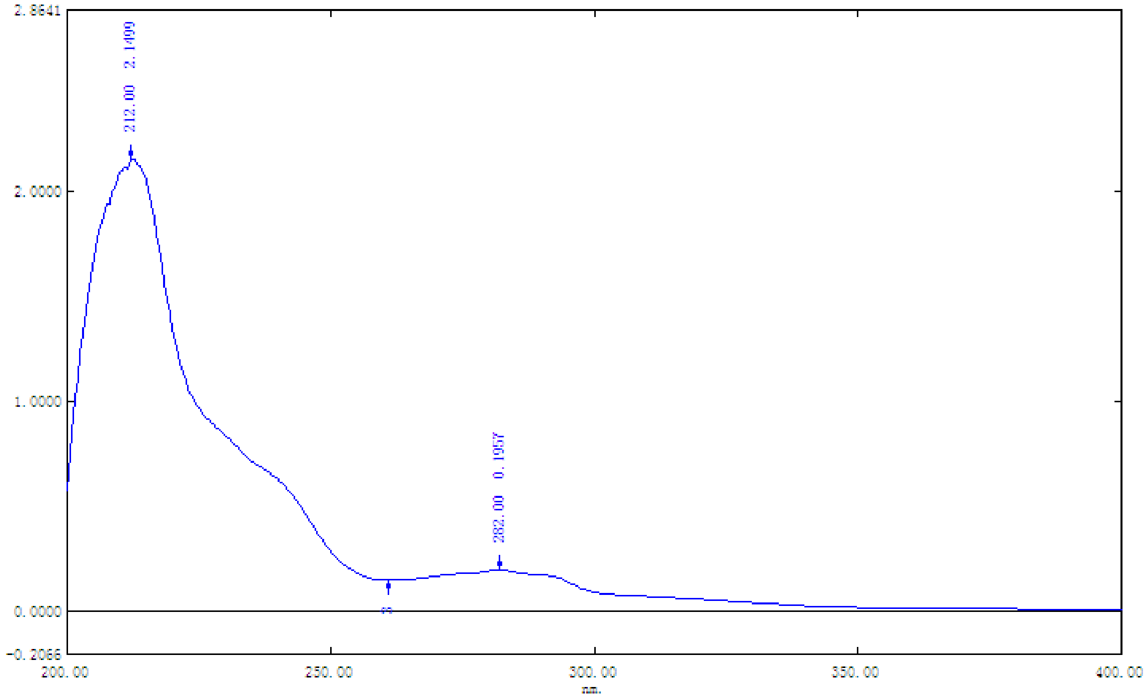

IR spectrum of compound 1

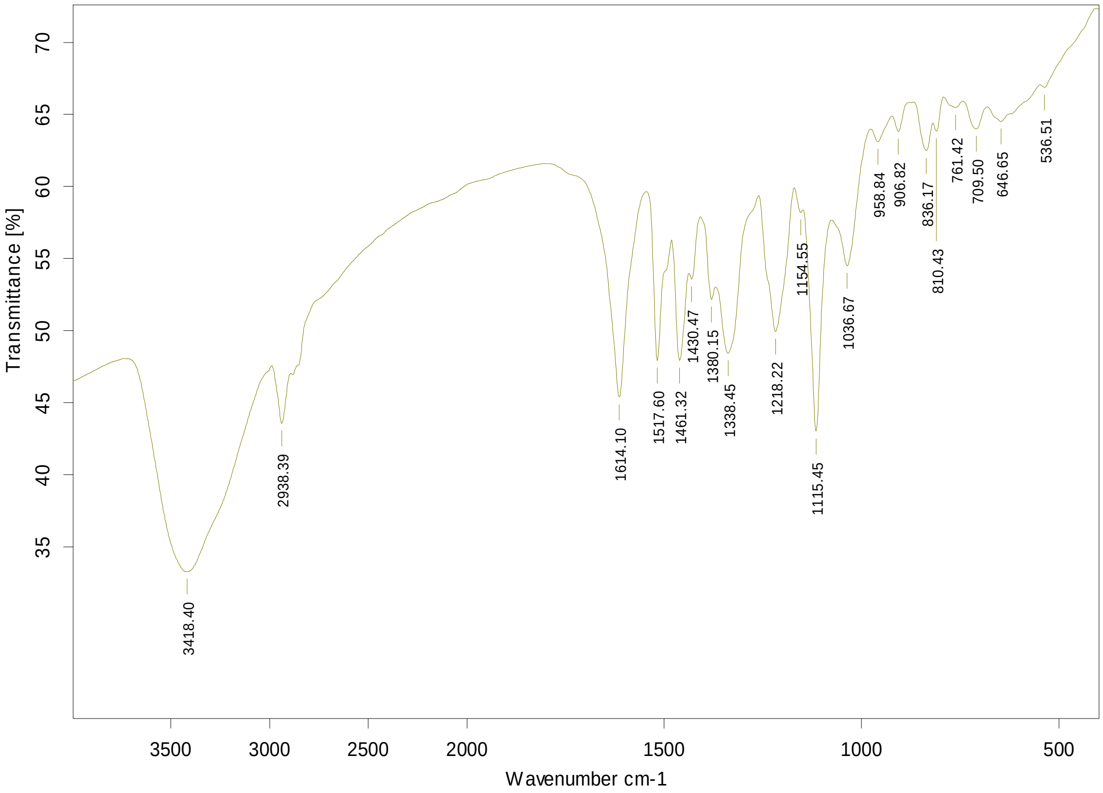

^{1}H NMR of compound 1

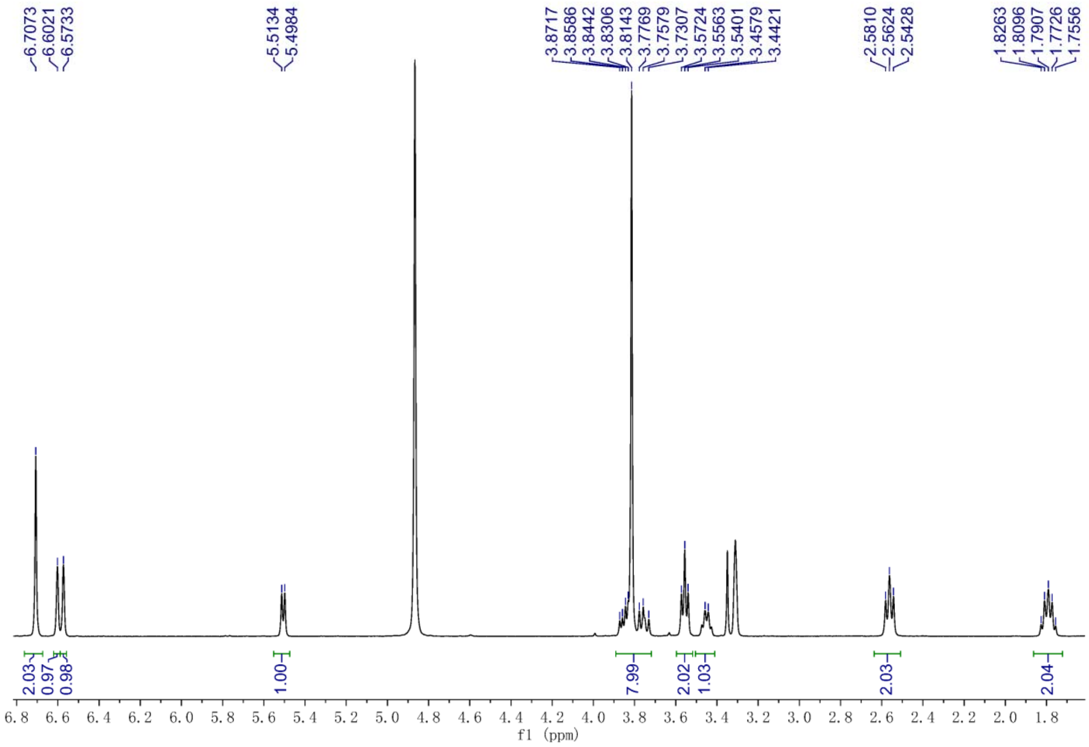

^{13}C NMR and DEPT of compound 1

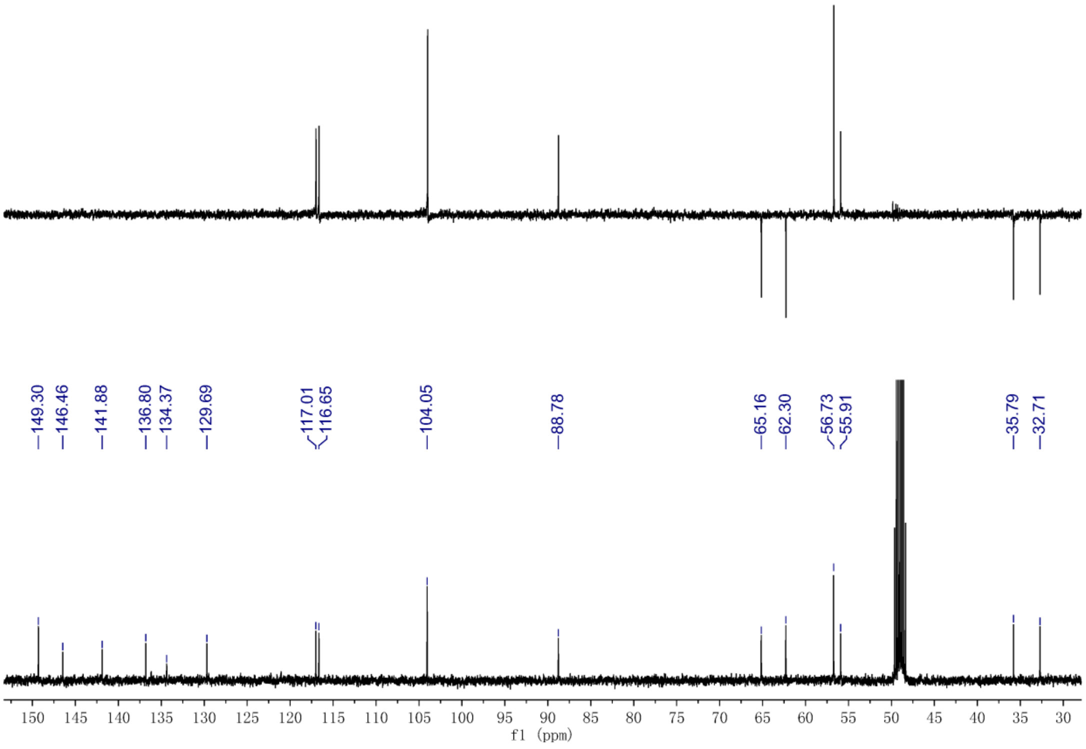

HSQC of compound 1

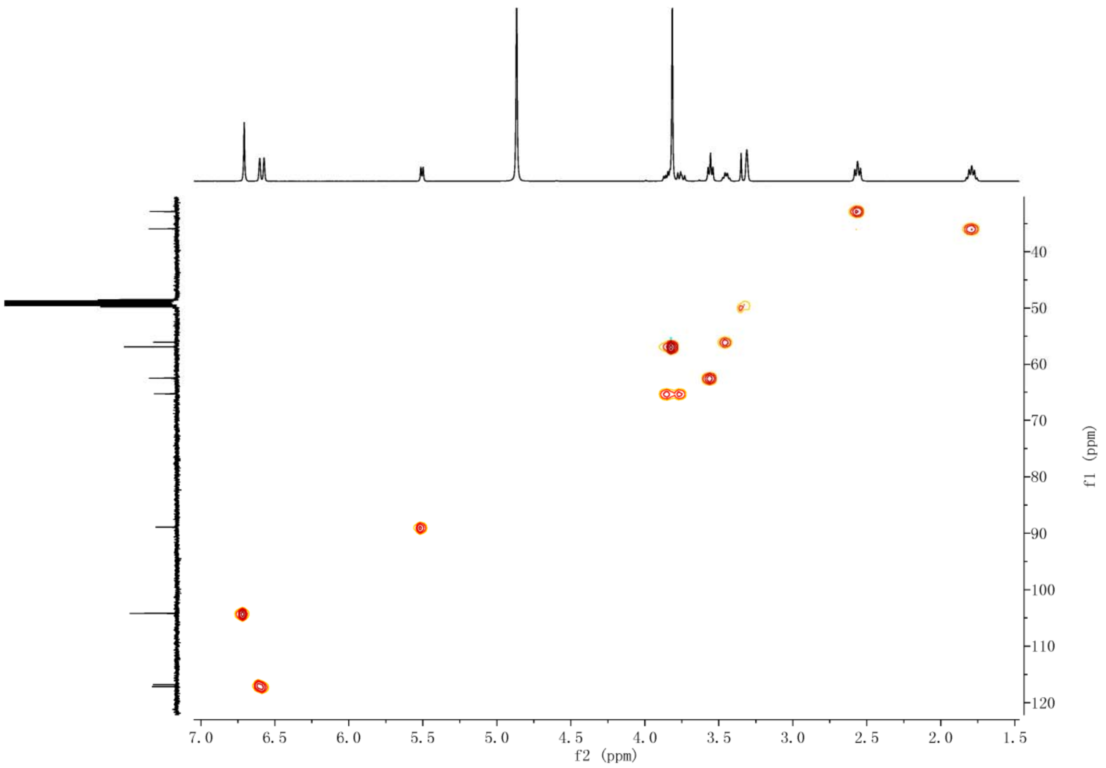

HMBC of compound 1

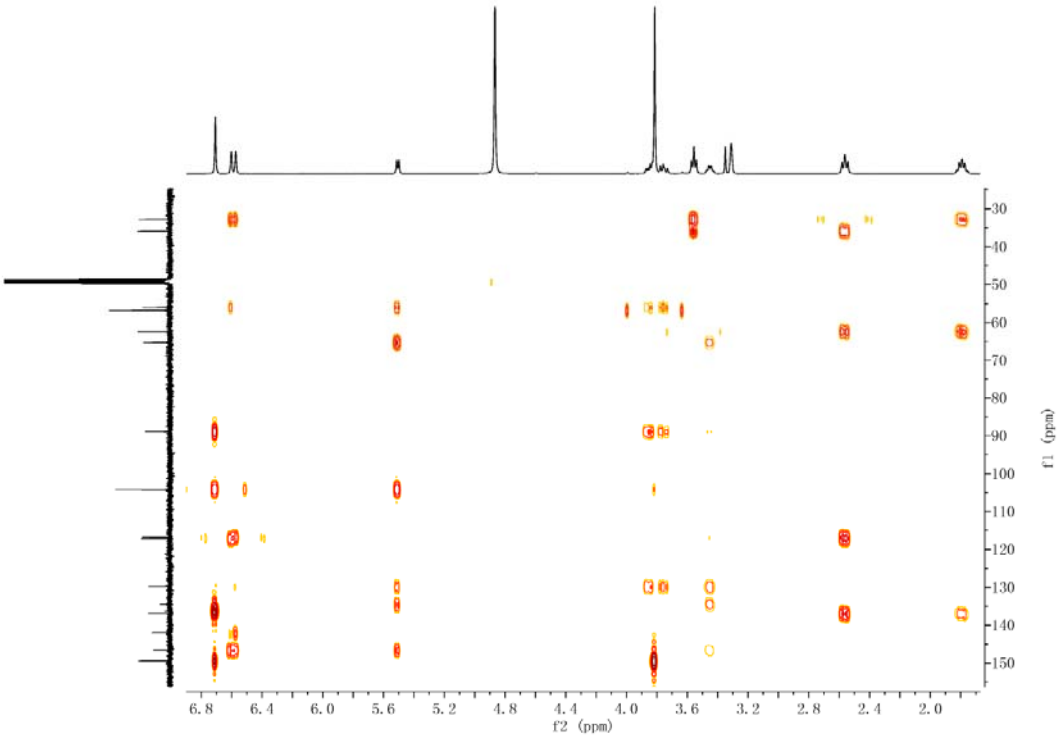

^{1}H–^{1}H COSY of compound 1

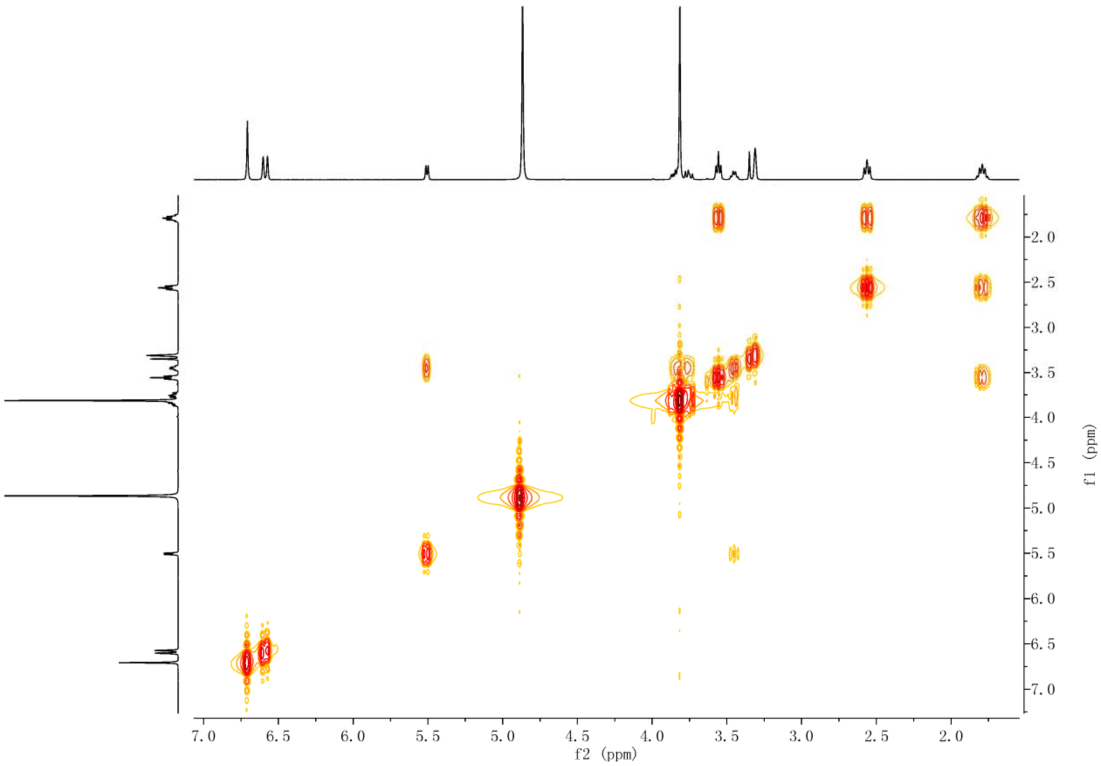

NOESY of compound 1

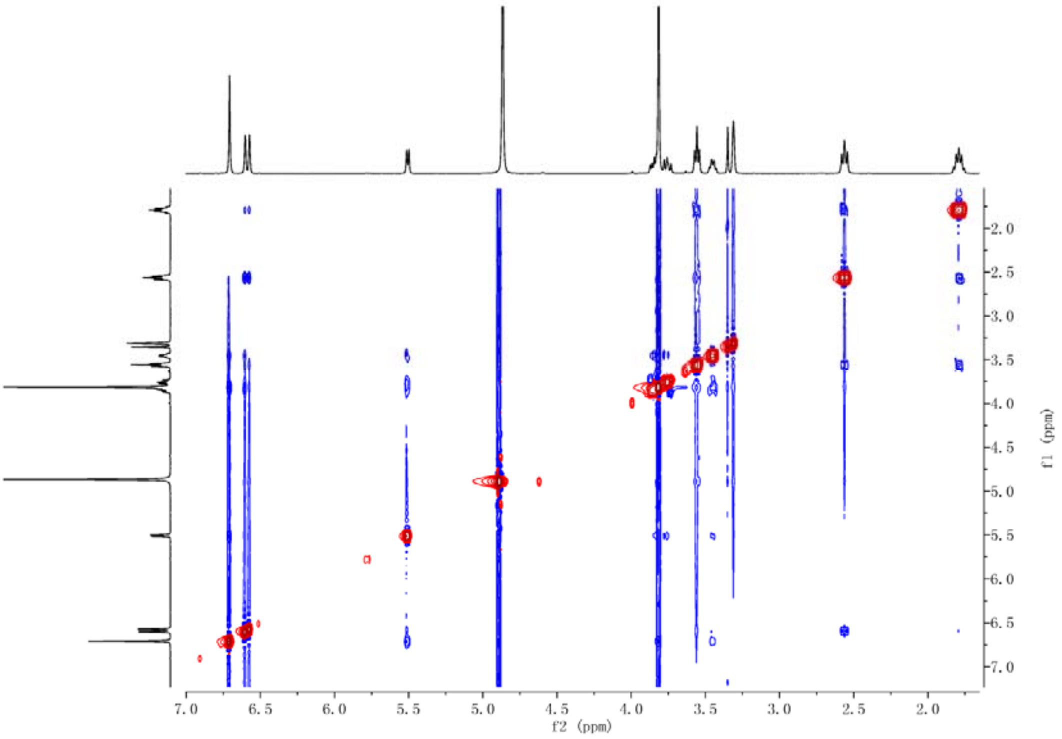

HRESIMS of compound 2

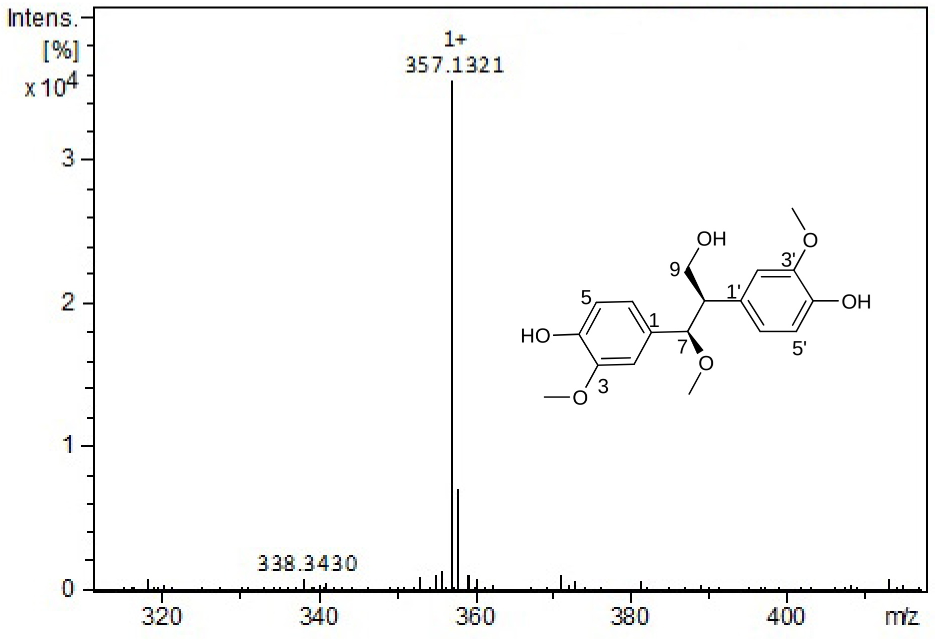

UV spectrum of compound 2

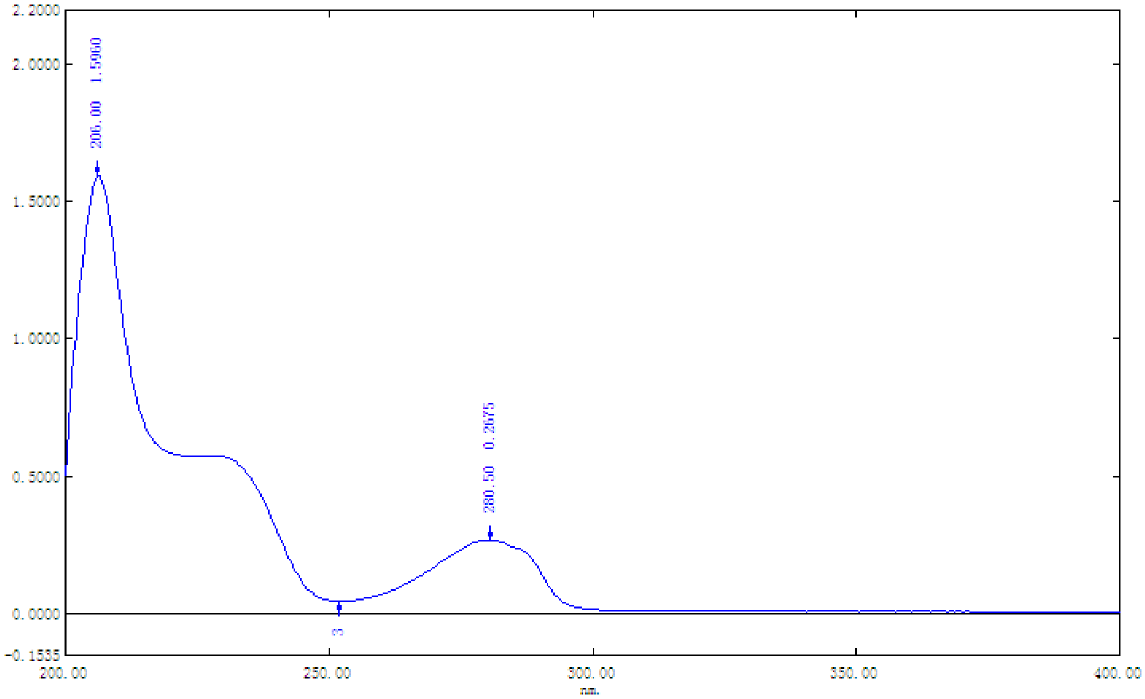

IR spectrum of compound 2

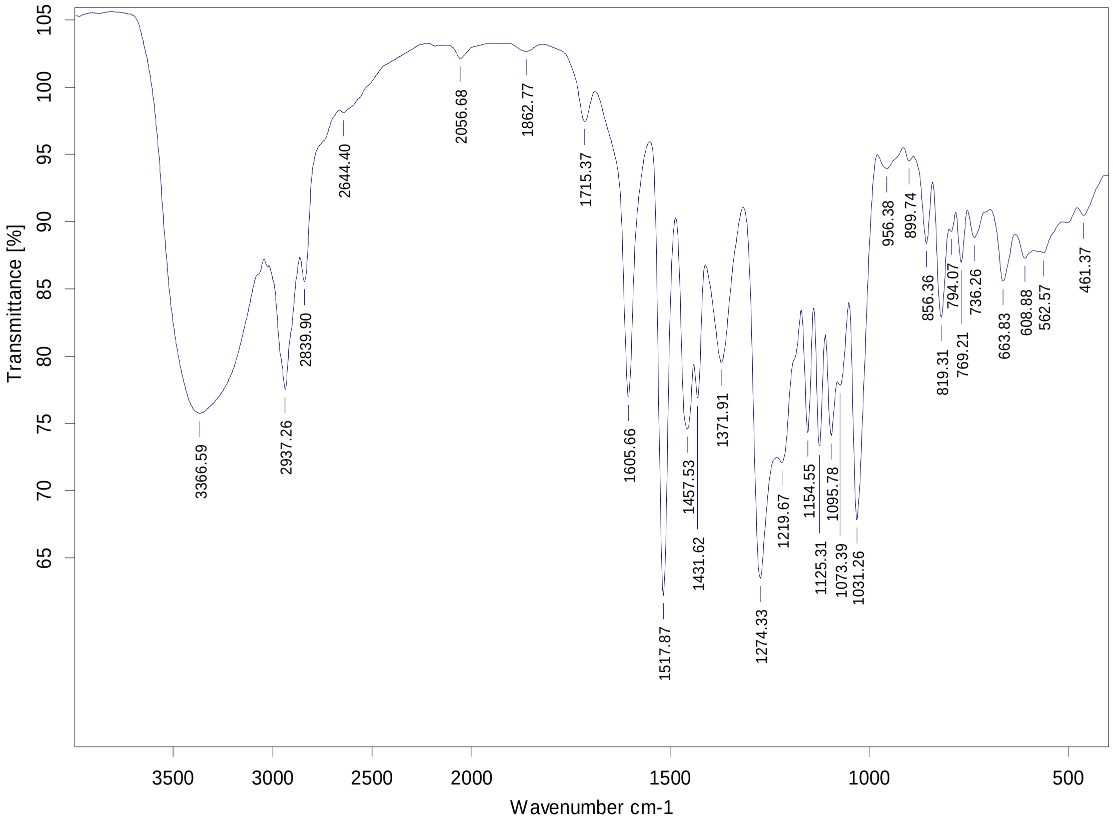

^{1}H NMR of compound 2

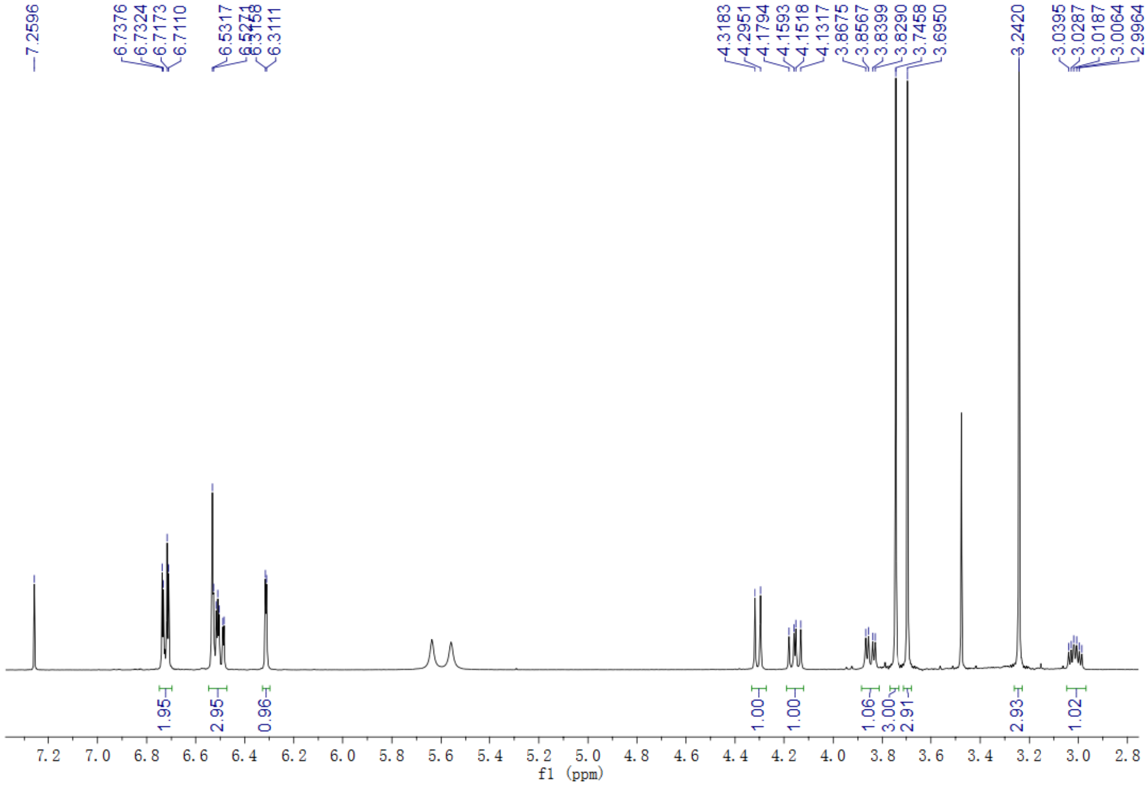

^{13}C NMR and DEPT of compound 2

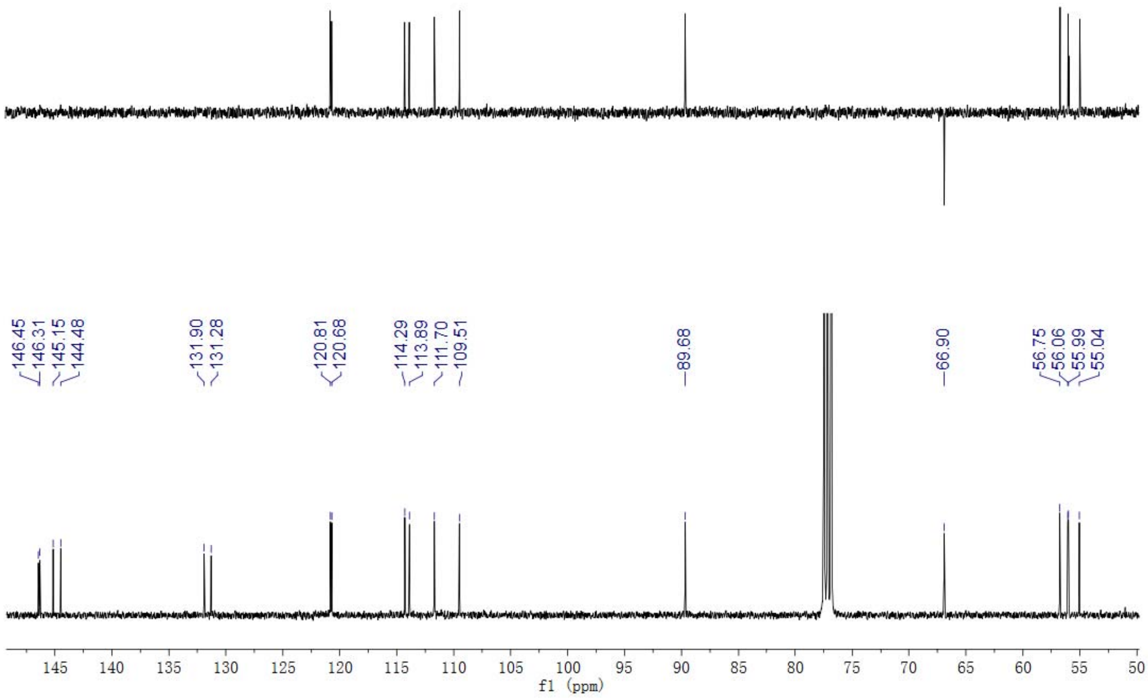

HSQC of compound 2

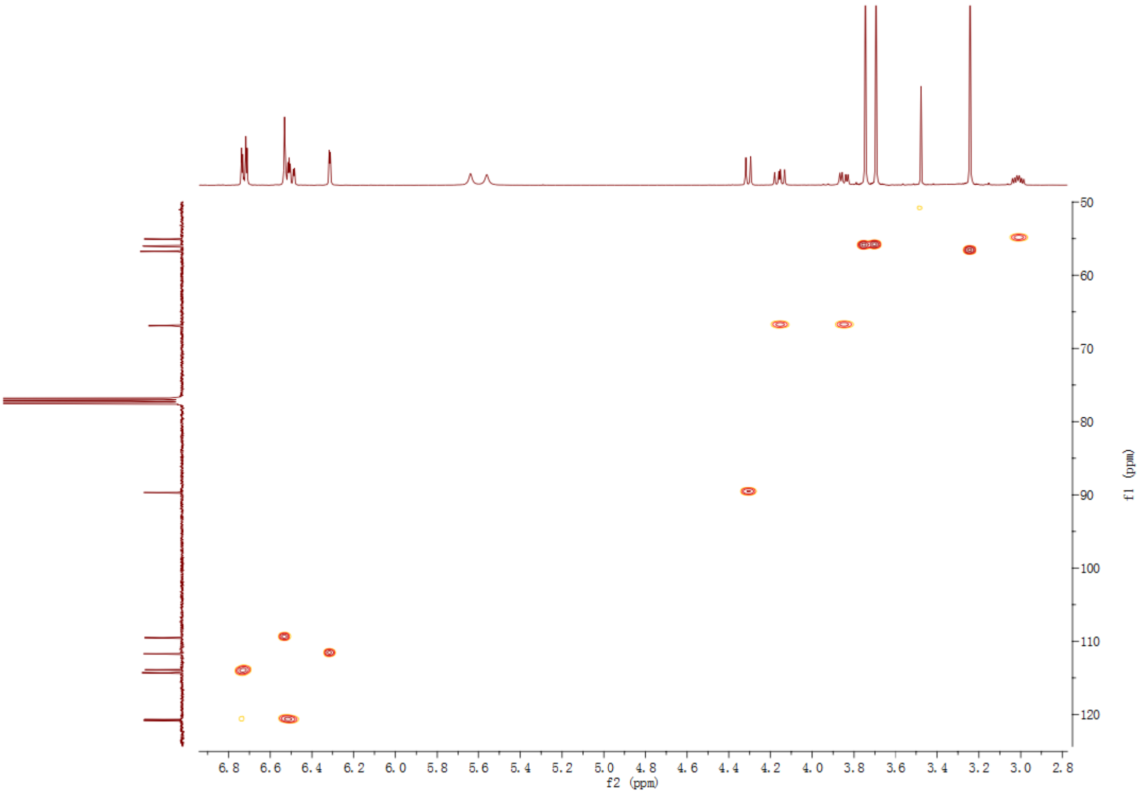

HMBC of compound 2

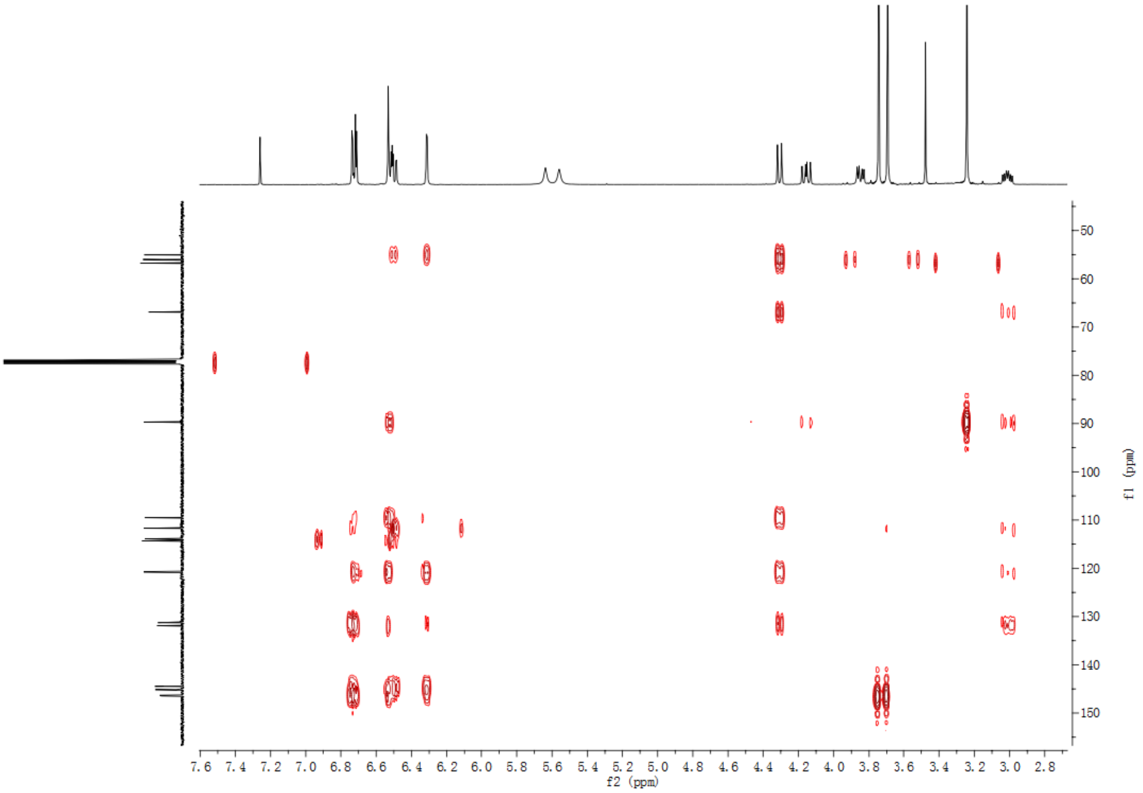

^{1}H–^{1}H COSY of compound 2

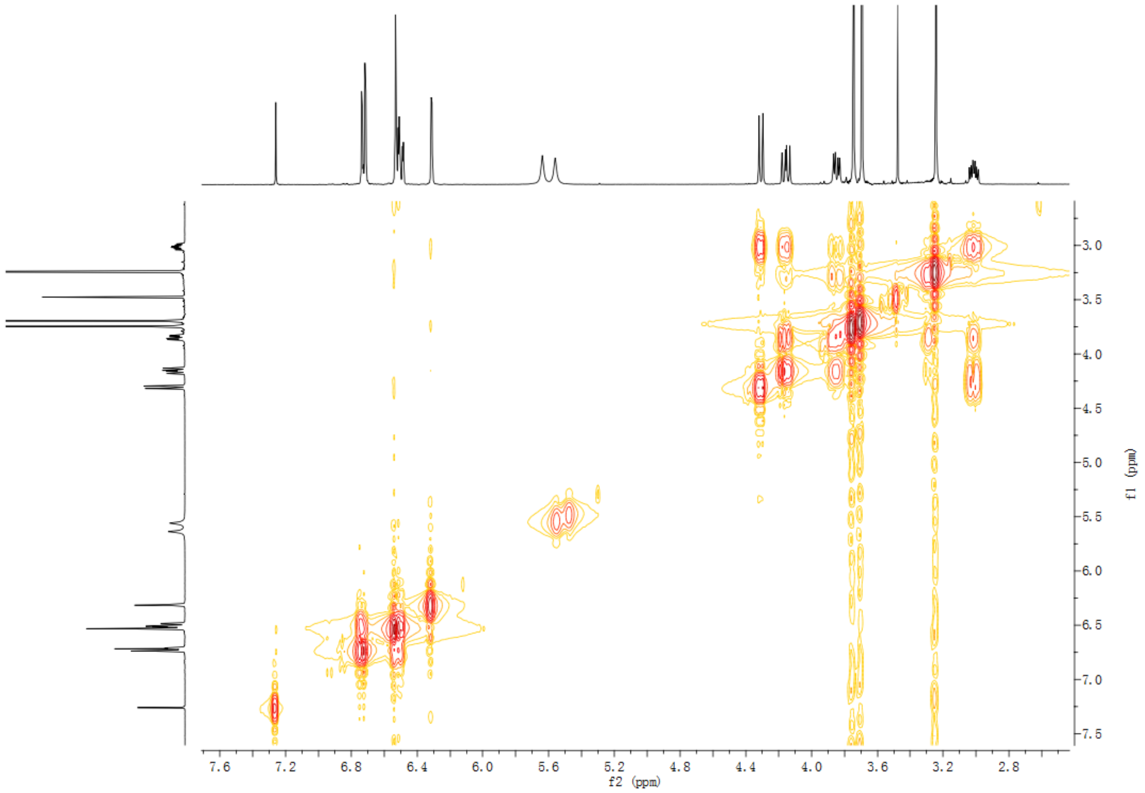

NOESY of compound 2

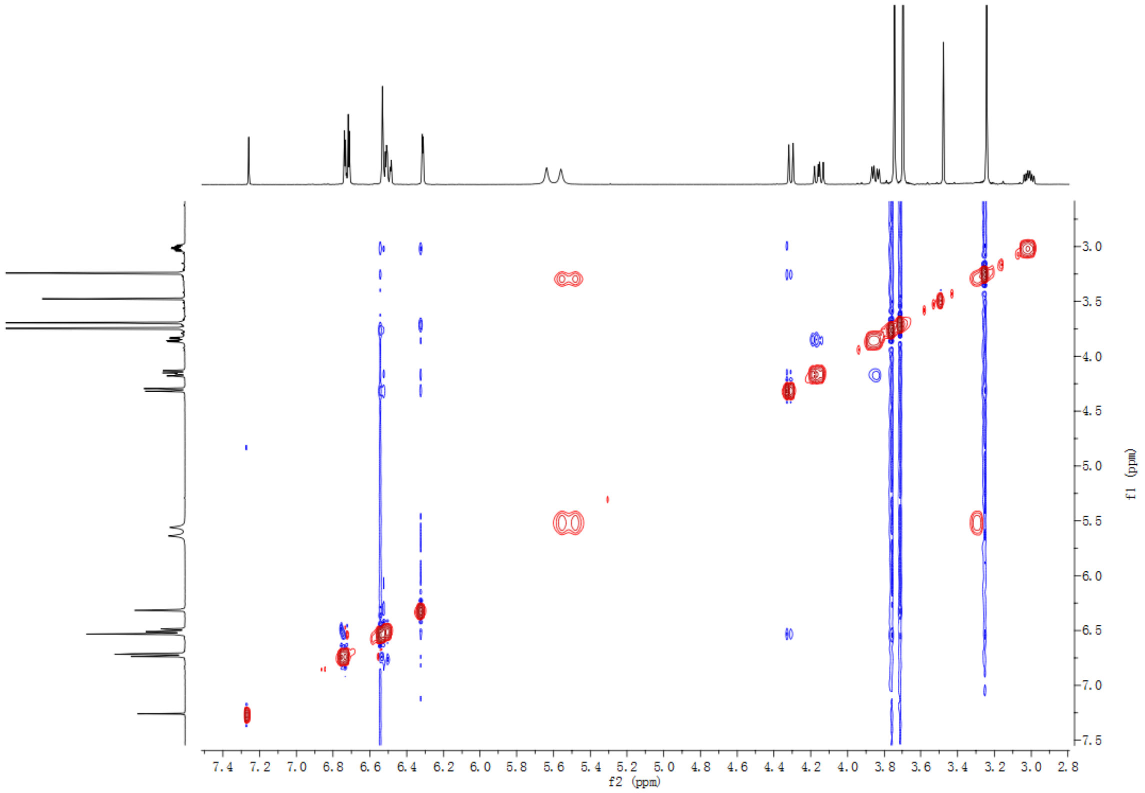

HRESIMS of compound 3

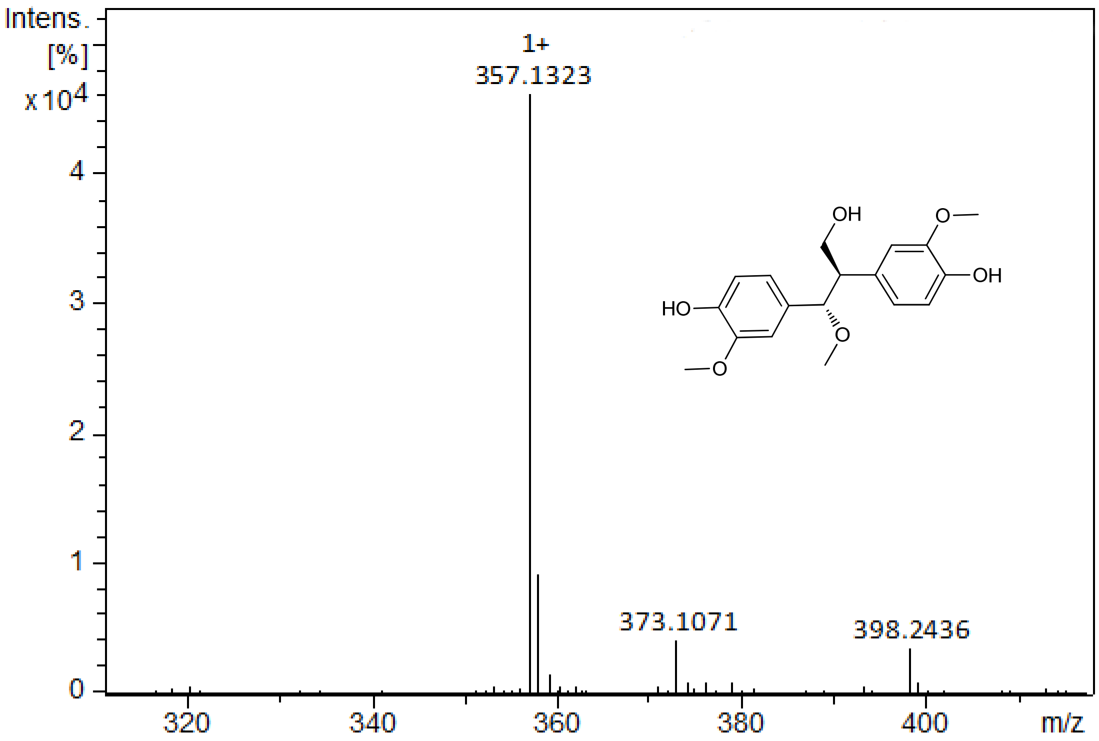

UV spectrum of compound 3

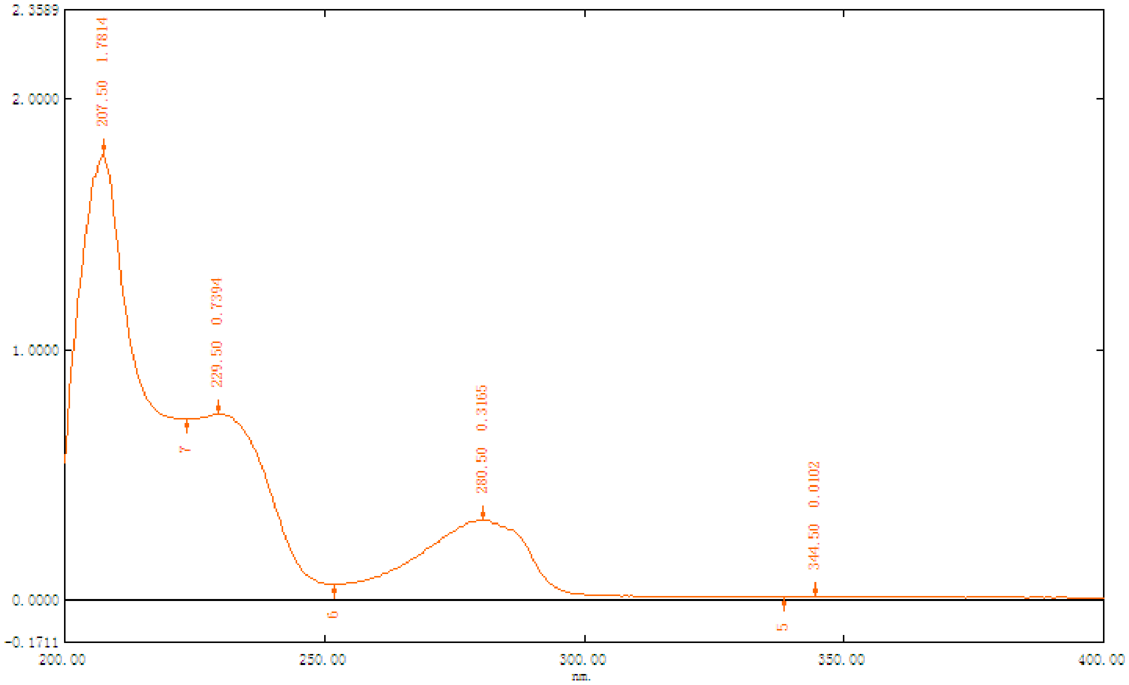

IR spectrum of compound 3

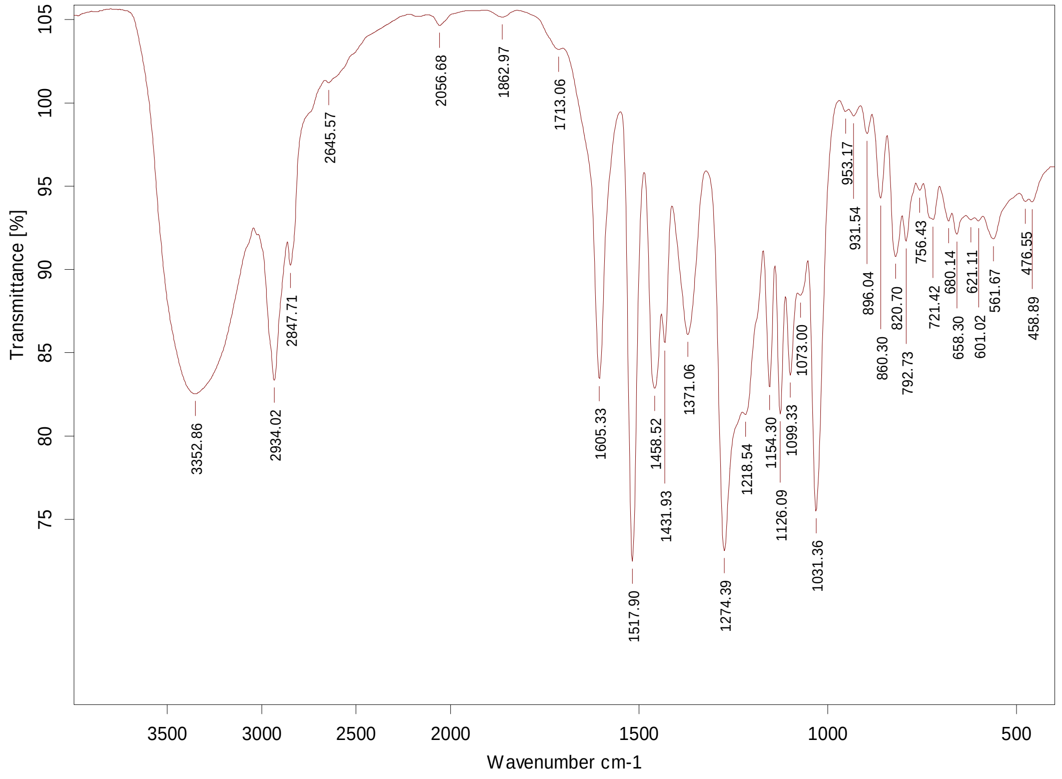

^{1}H NMR of compound 3

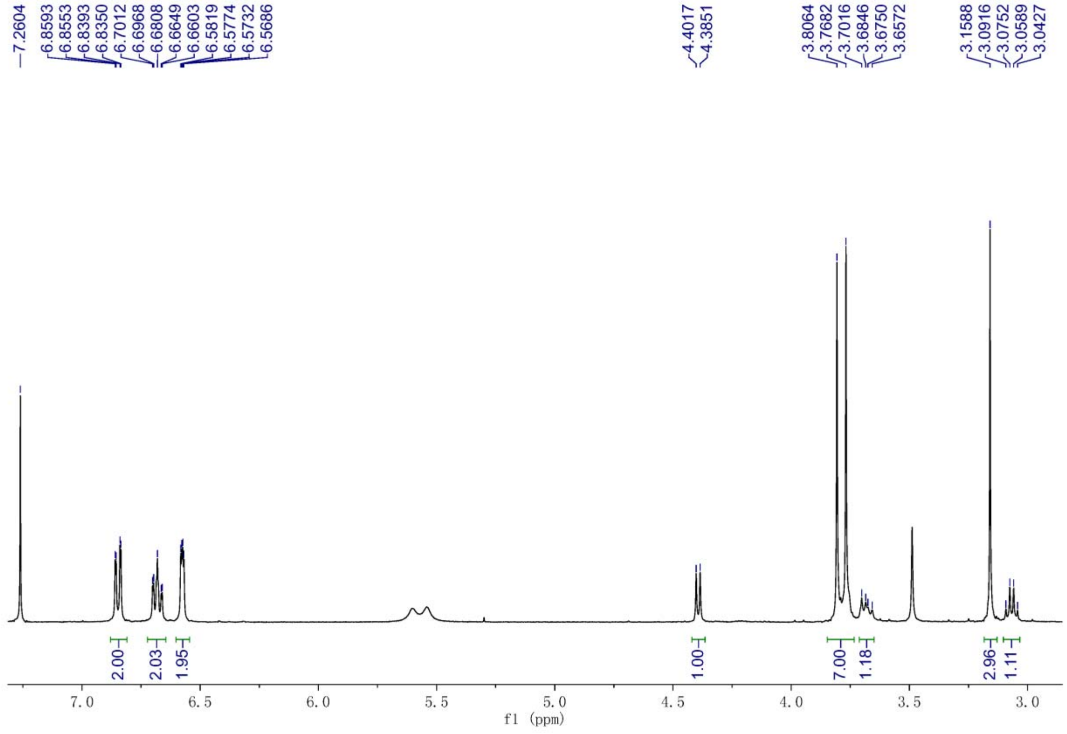

^{13}C NMR and DEPT of compound 3

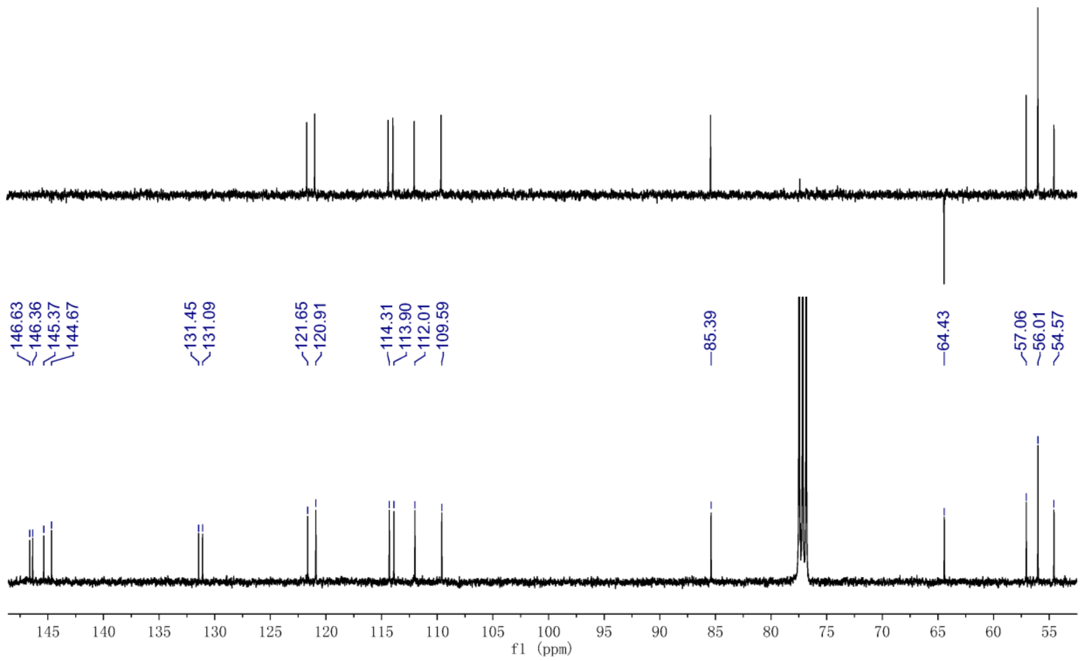

HSQC of compound 3

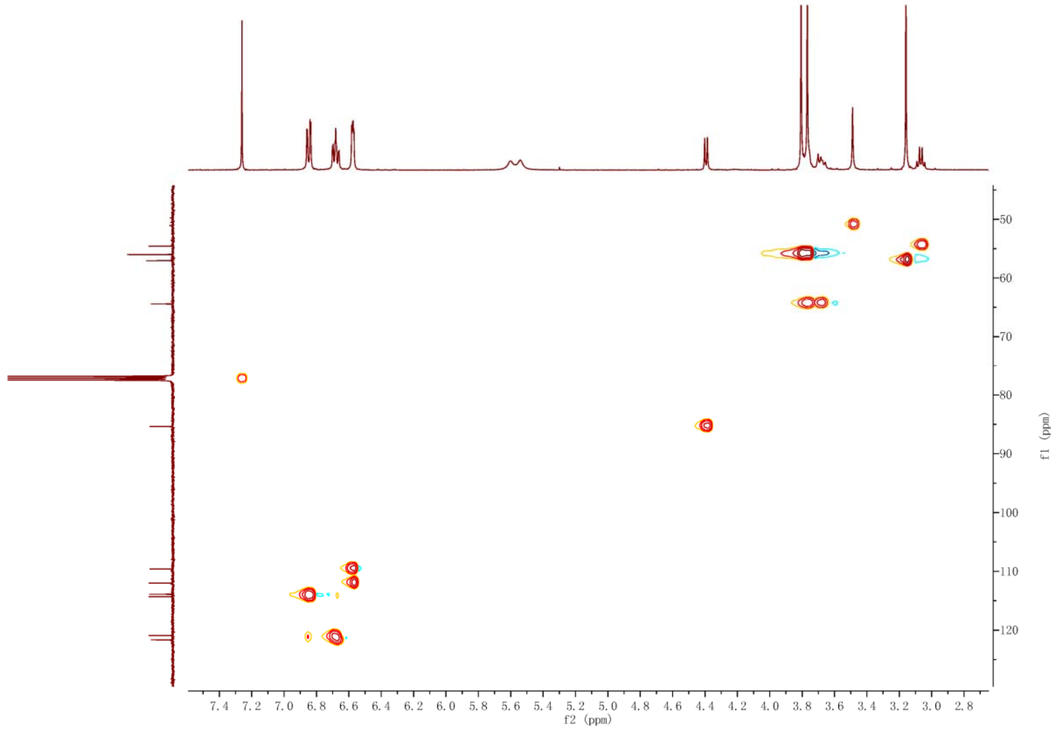

HMBC of compound 3

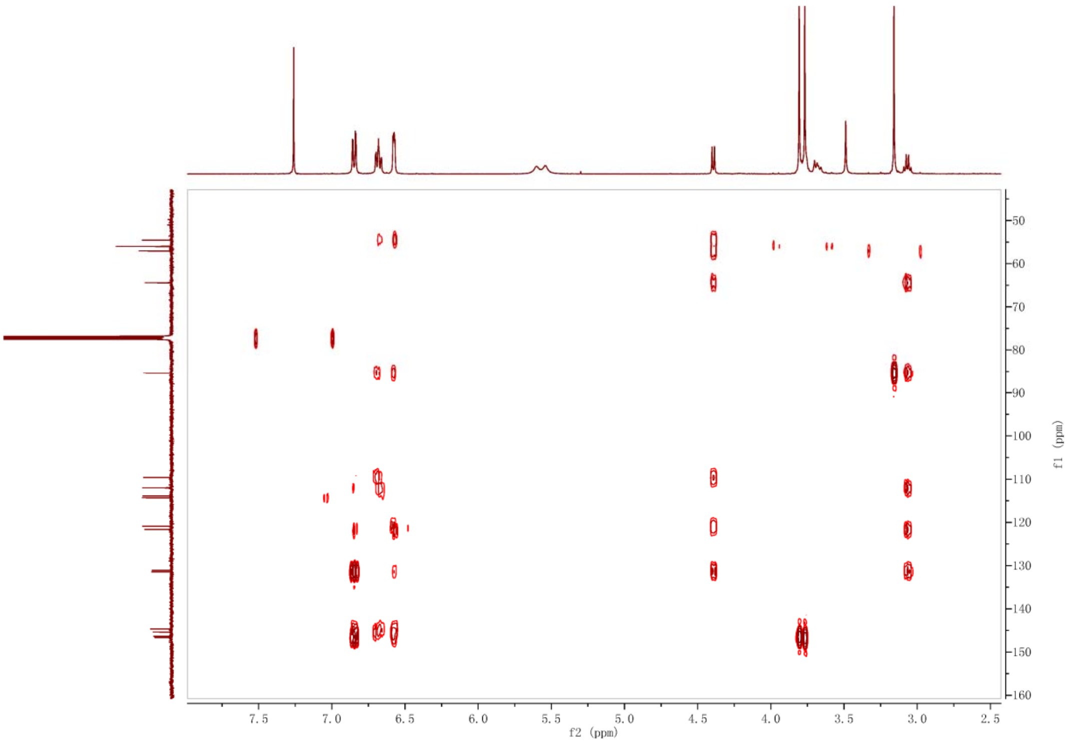

^{1}H–^{1}H COSY of compound 3

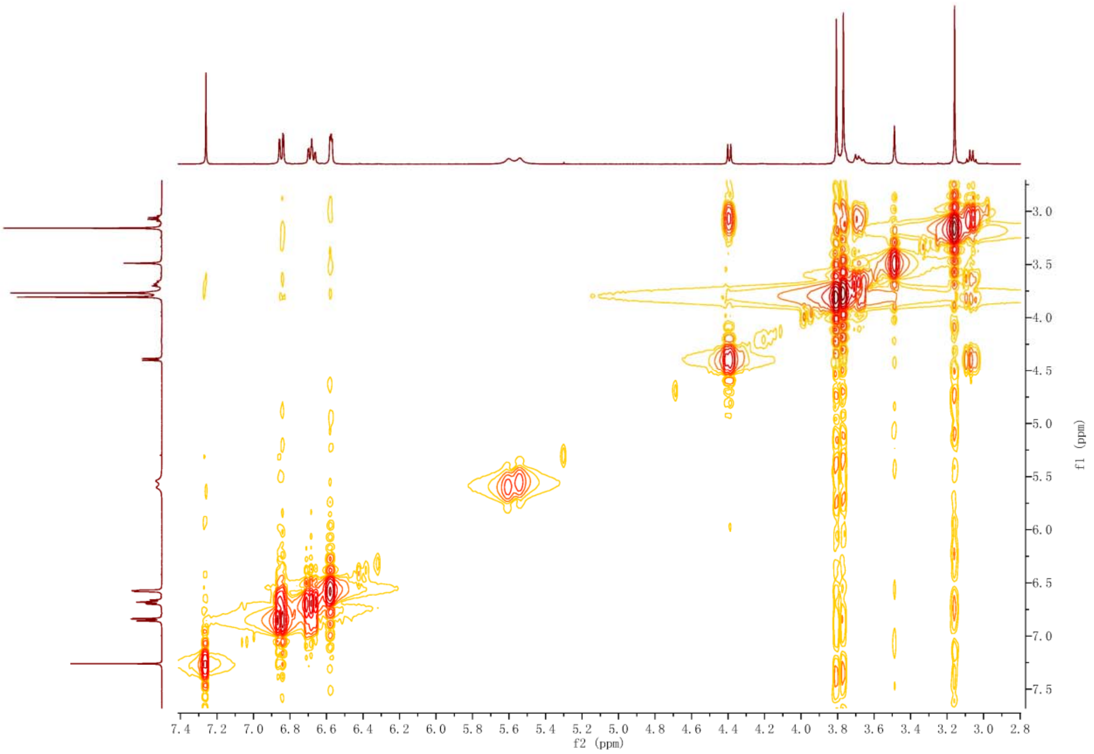

NOESY of compound 3

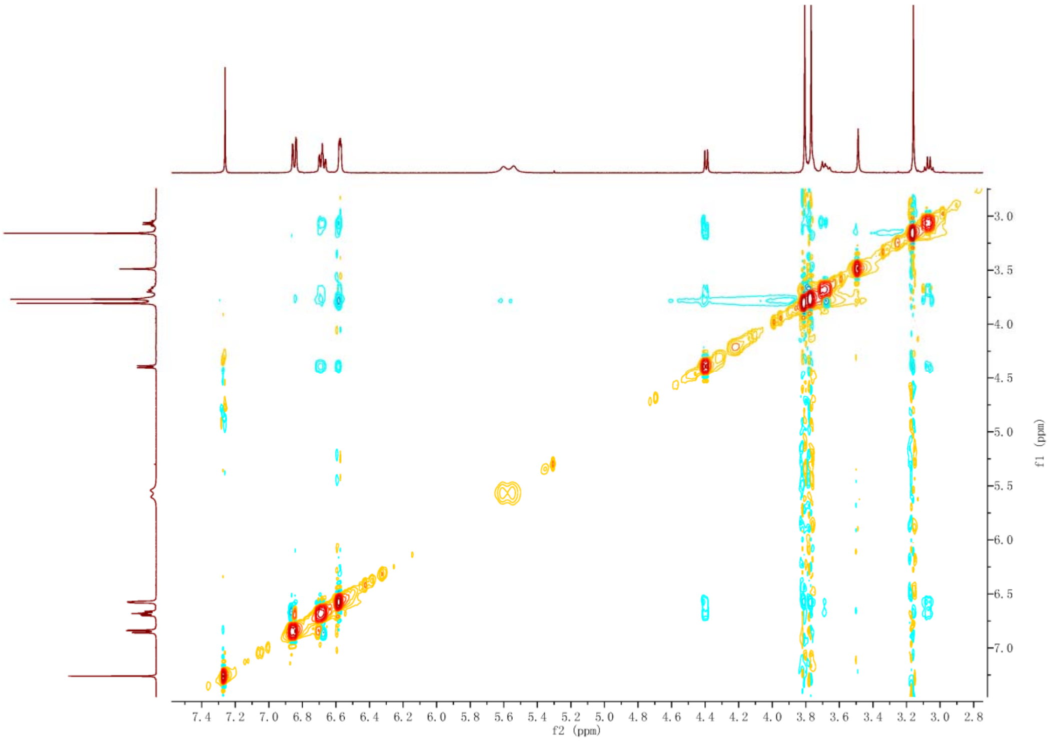

HRESIMS of compound 4

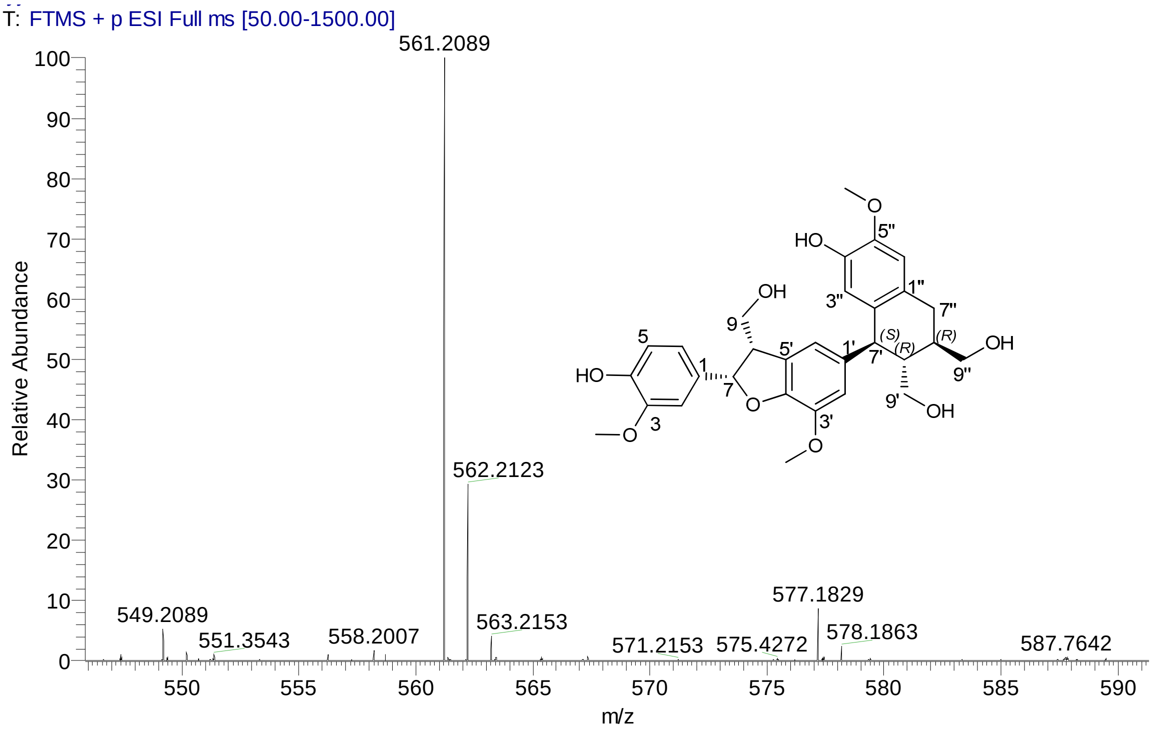

UV spectrum of compound 4

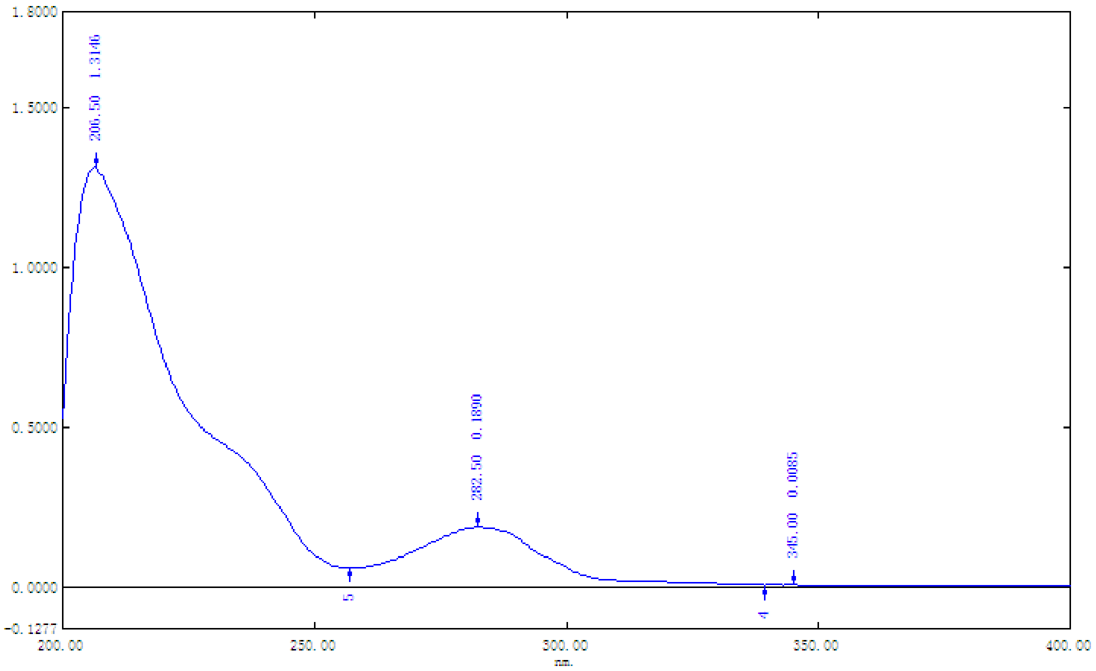

IR spectrum of compound 4

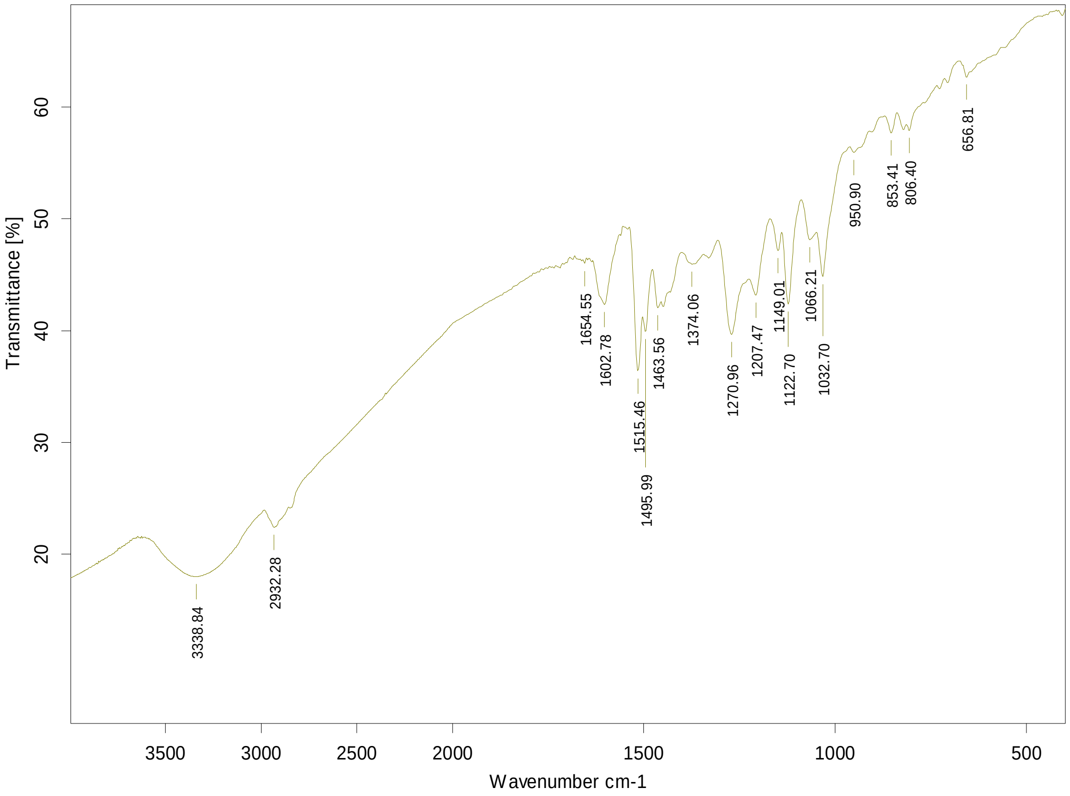

^{1}H NMR of compound 4

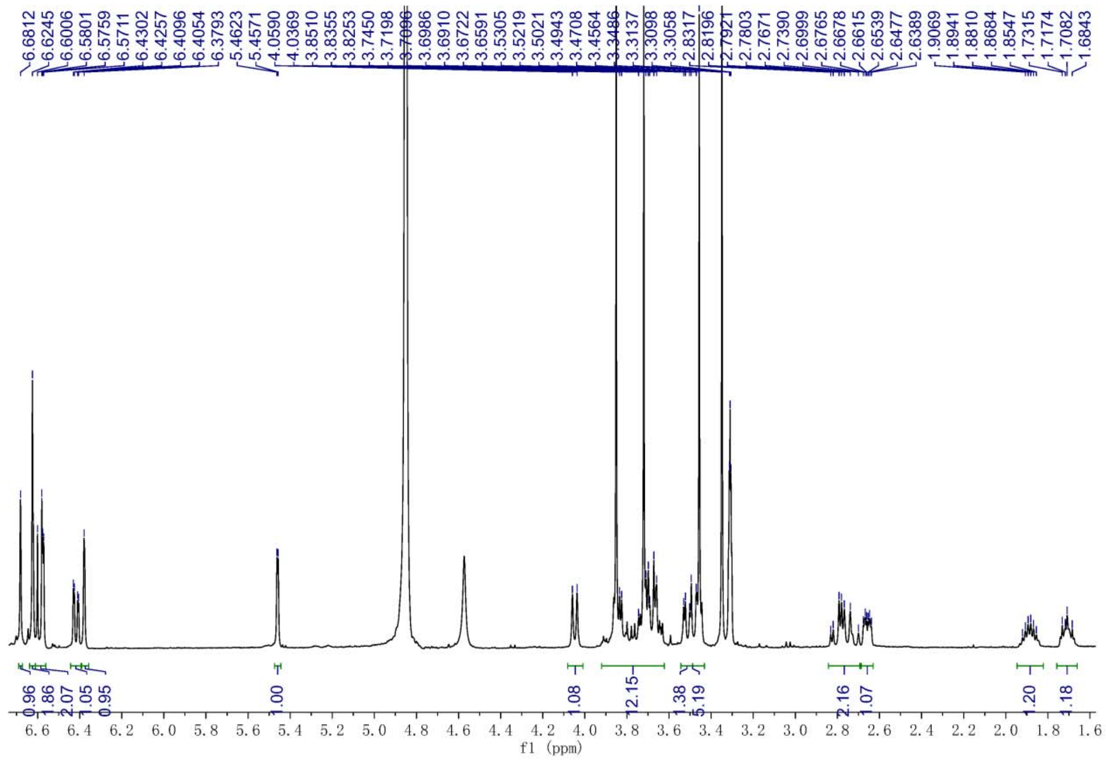

^{13}C NMR and DEPT of compound 4

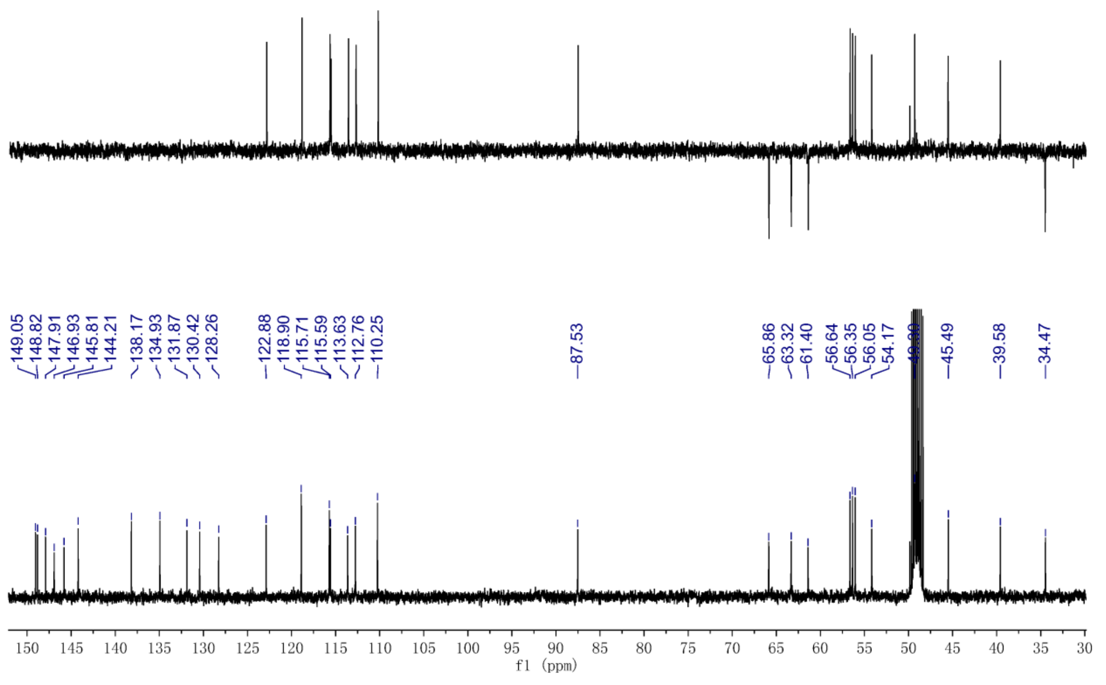

HSQC of compound 4

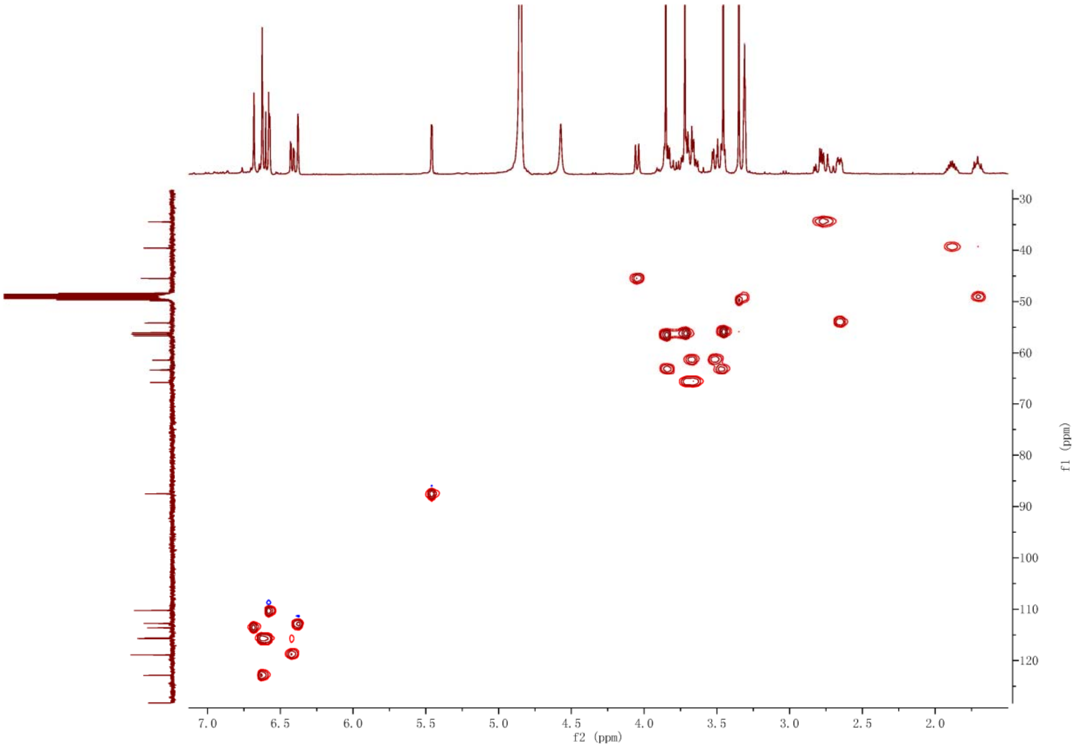

HMBC of compound 4

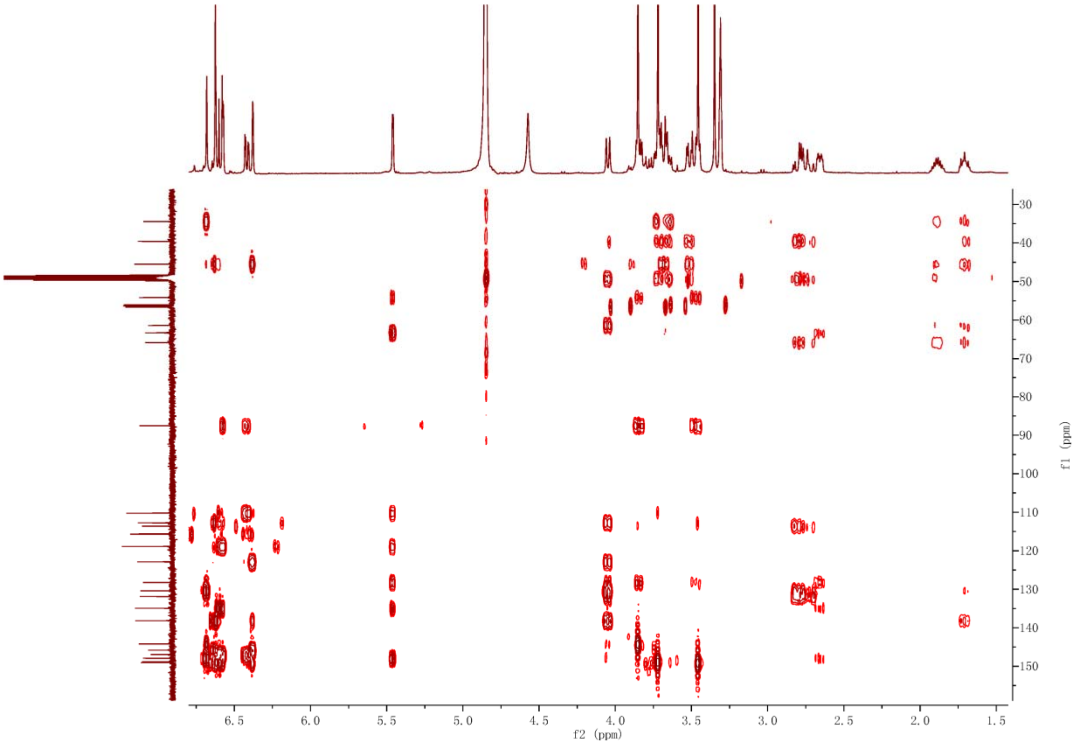

^{1}H–^{1}H COSY of compound 4

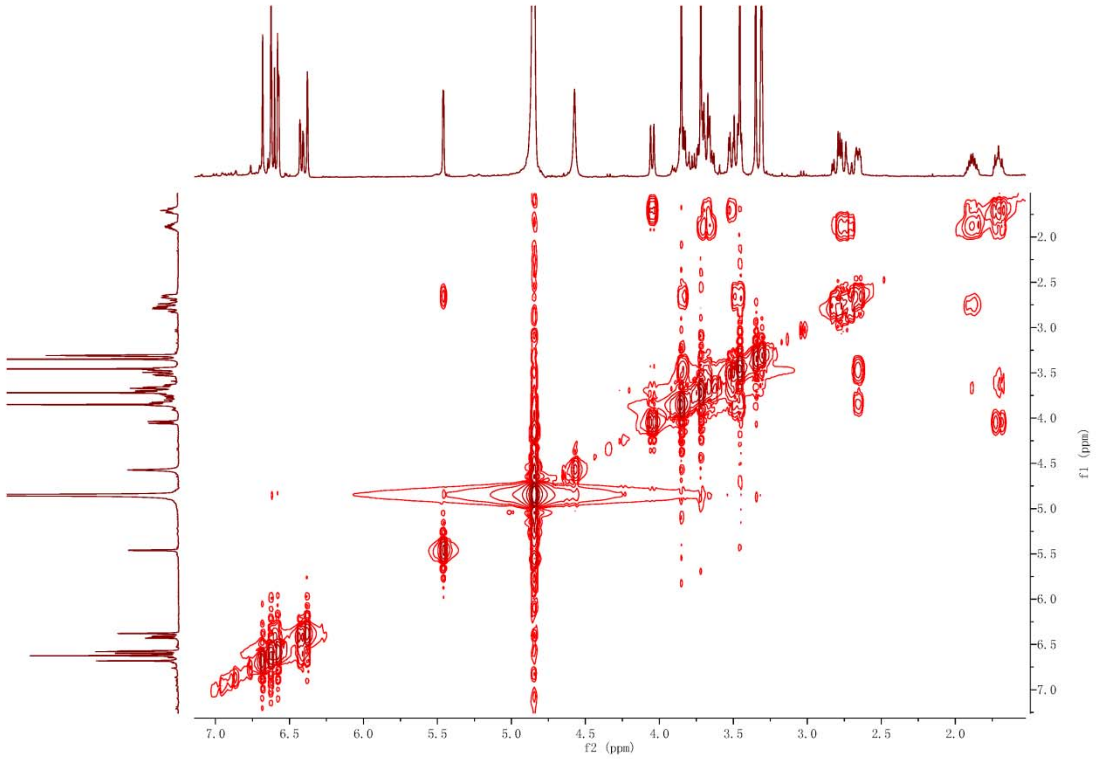

NOESY of compound 4

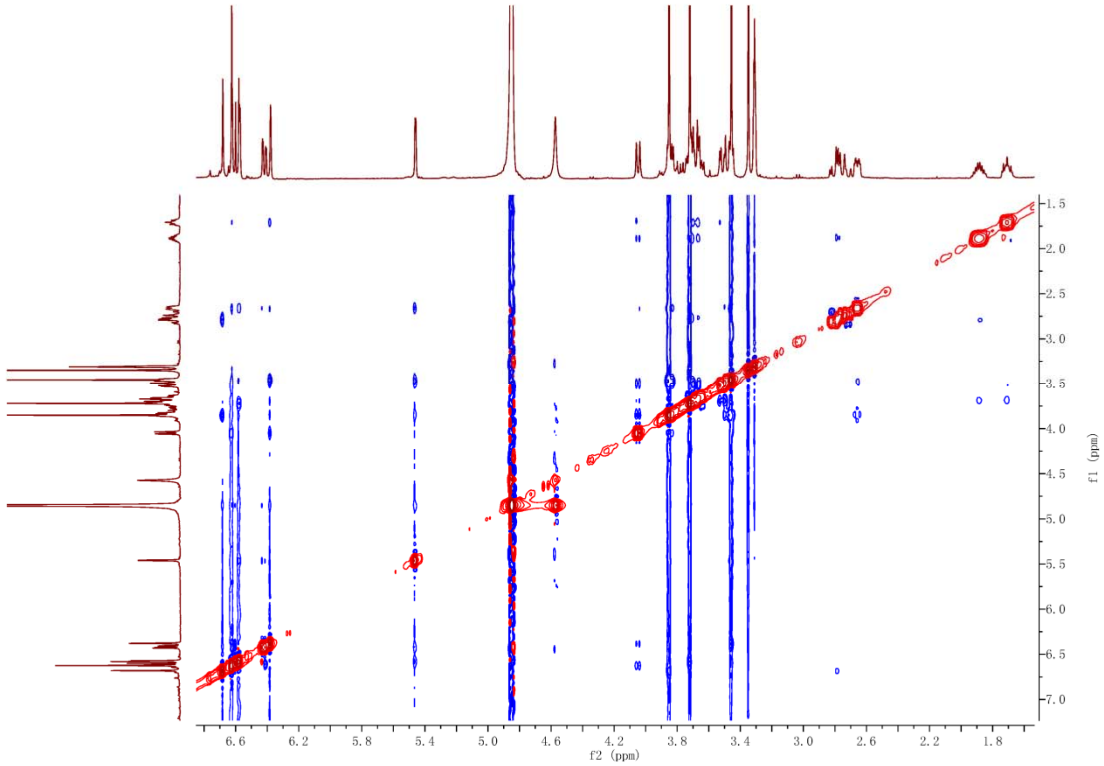

HRESIMS of compound 5

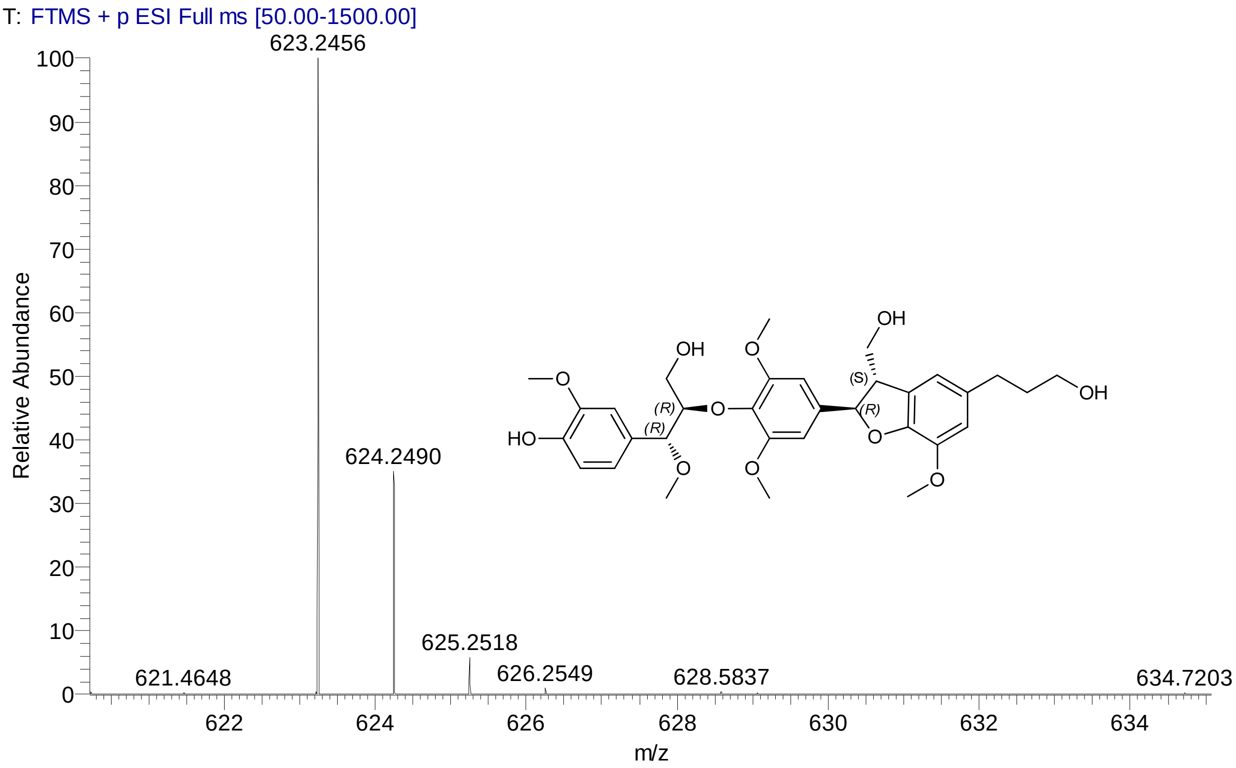

UV spectrum of compound 5

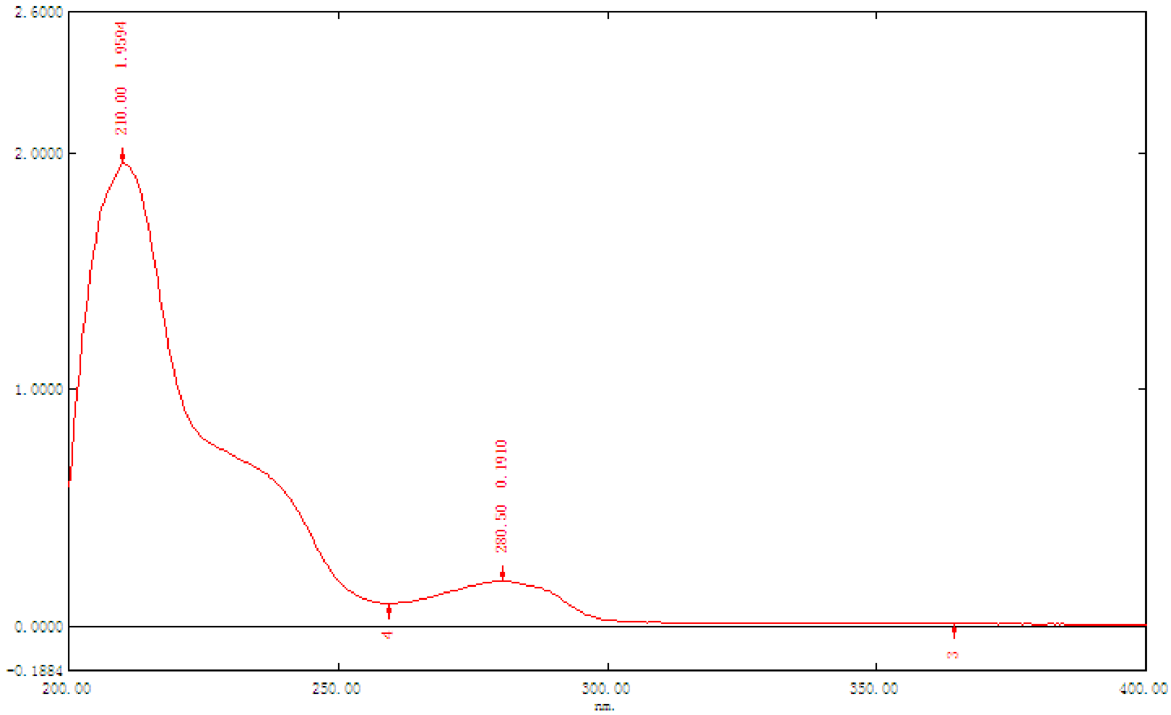

IR spectrum of compound 5

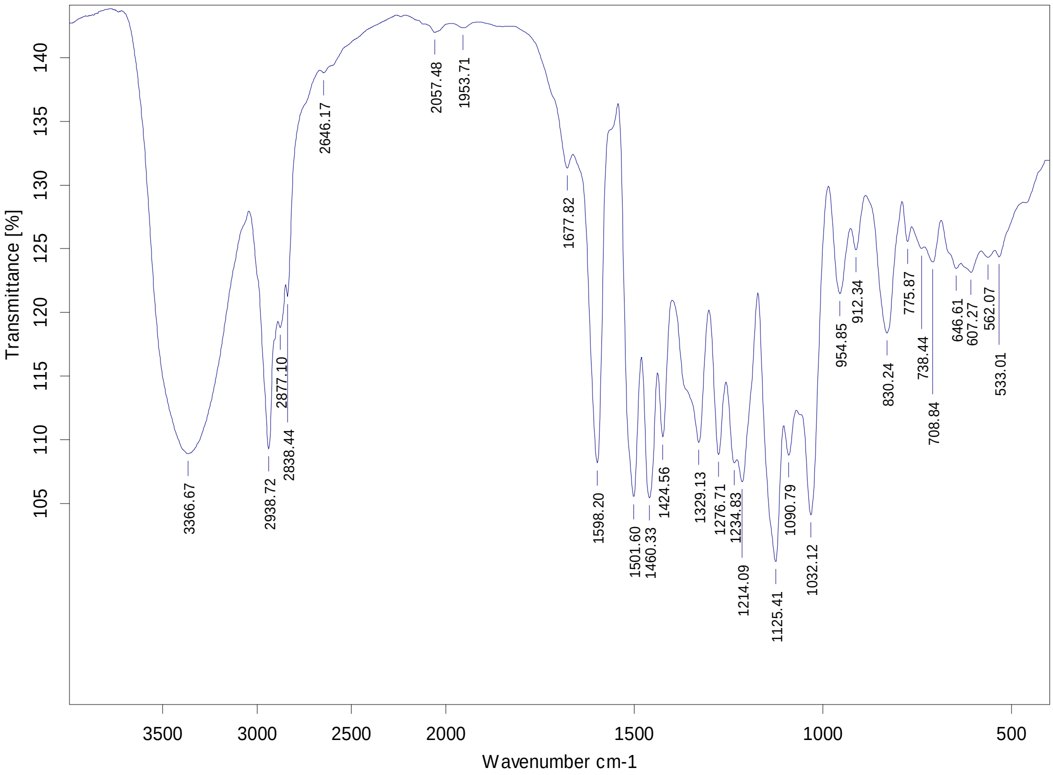

^{1}H NMR of compound 5

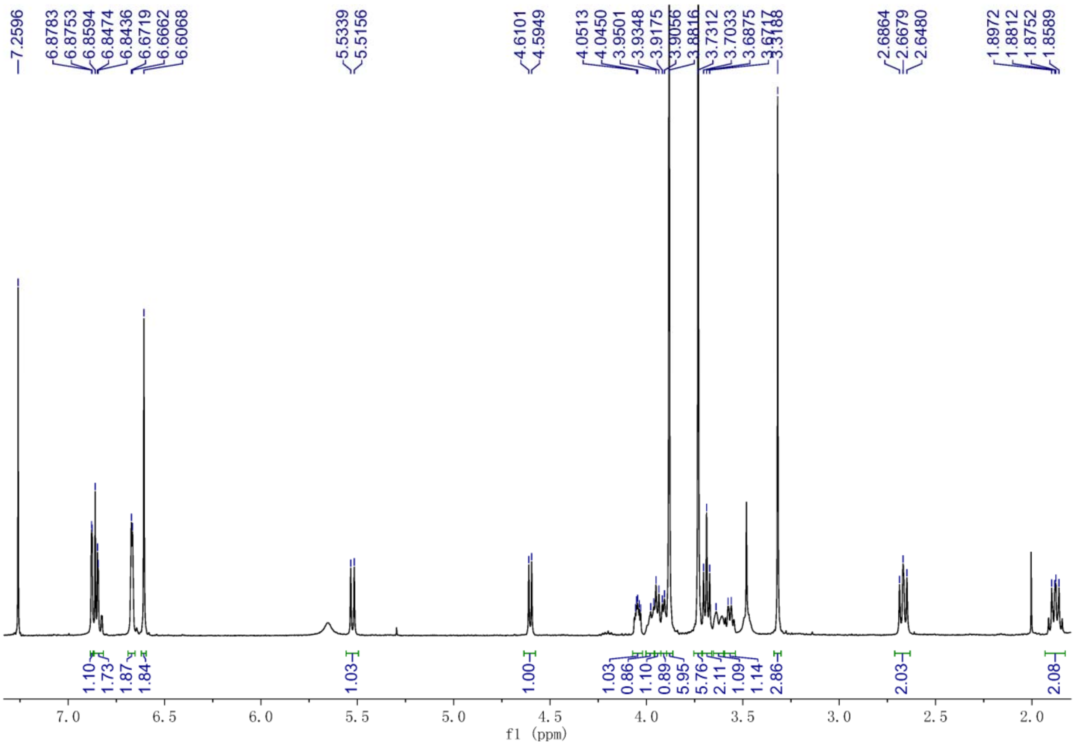

^{13}C NMR and DEPT of compound 5

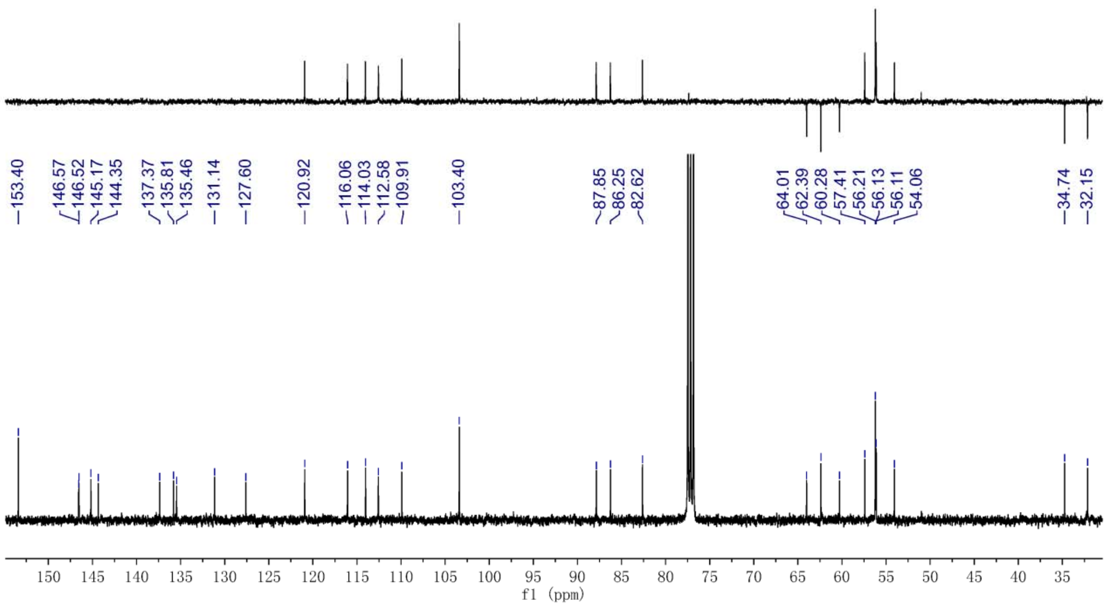

HSQC of compound 5

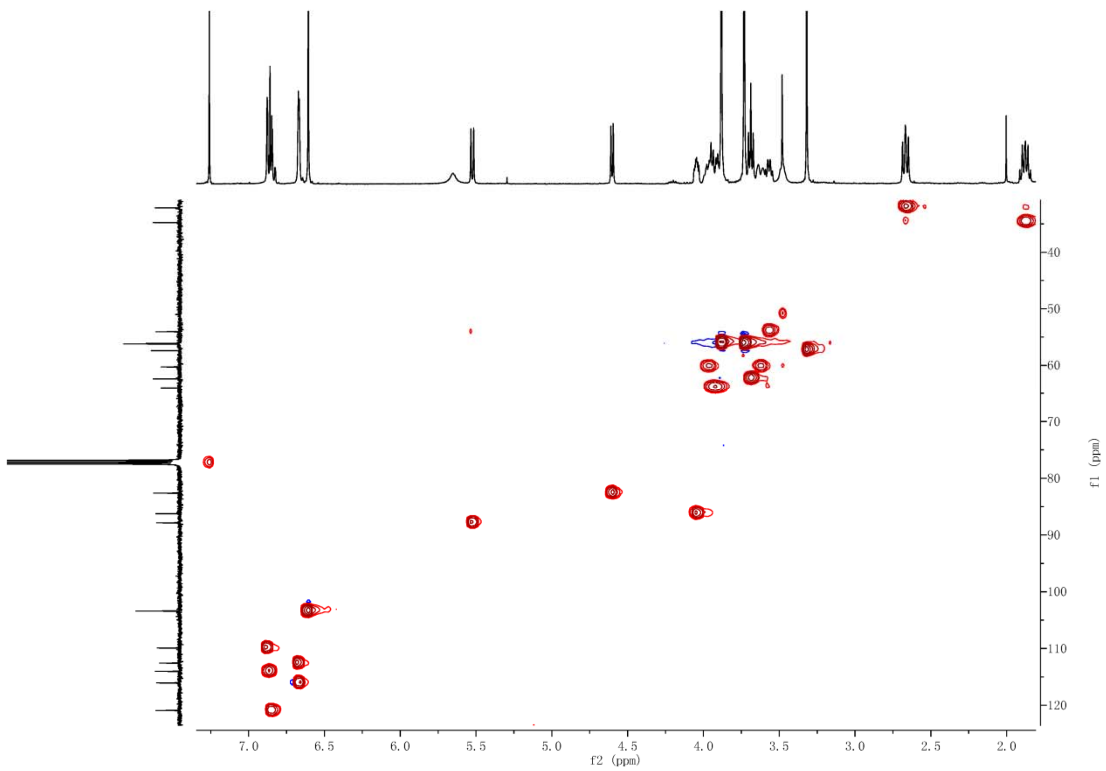

HMBC of compound 5

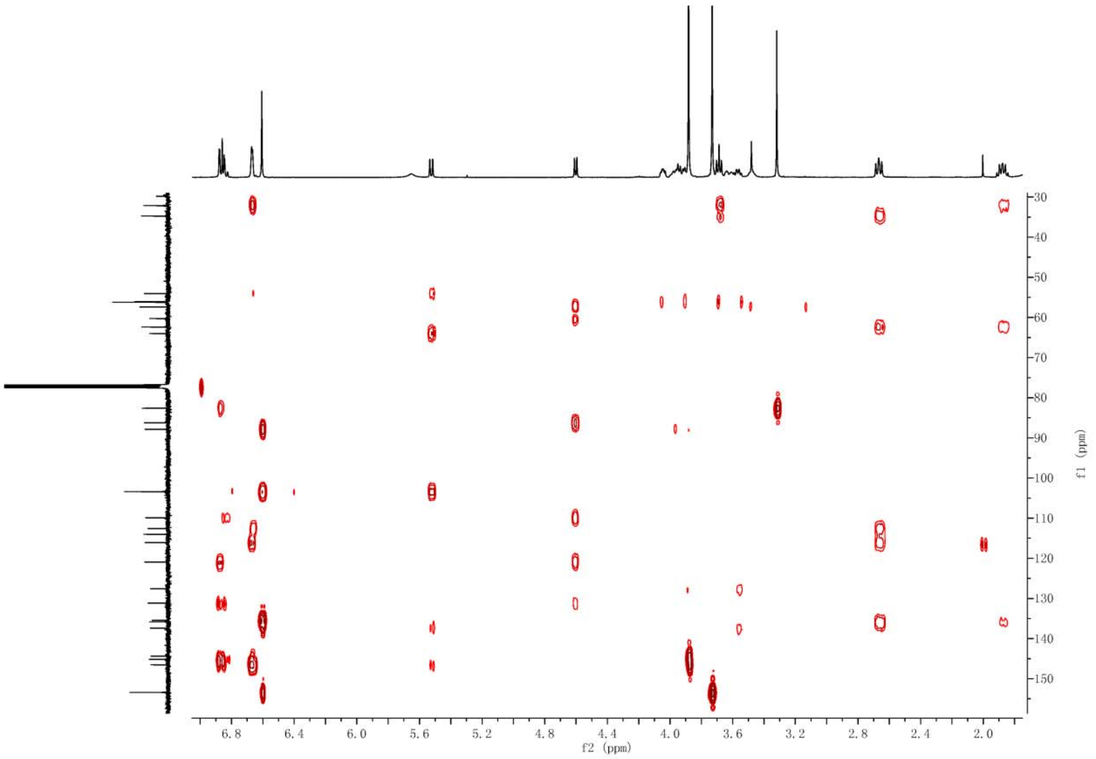

^{1}H–^{1}H COSY of compound 5

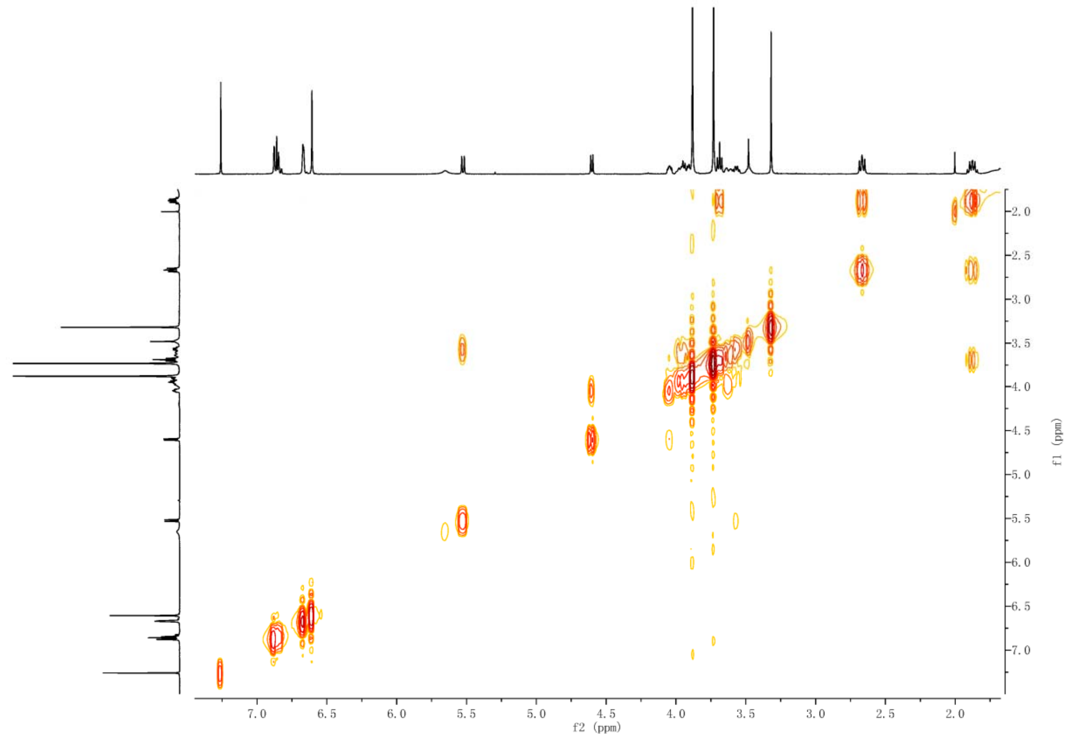

NOESY of compound 5

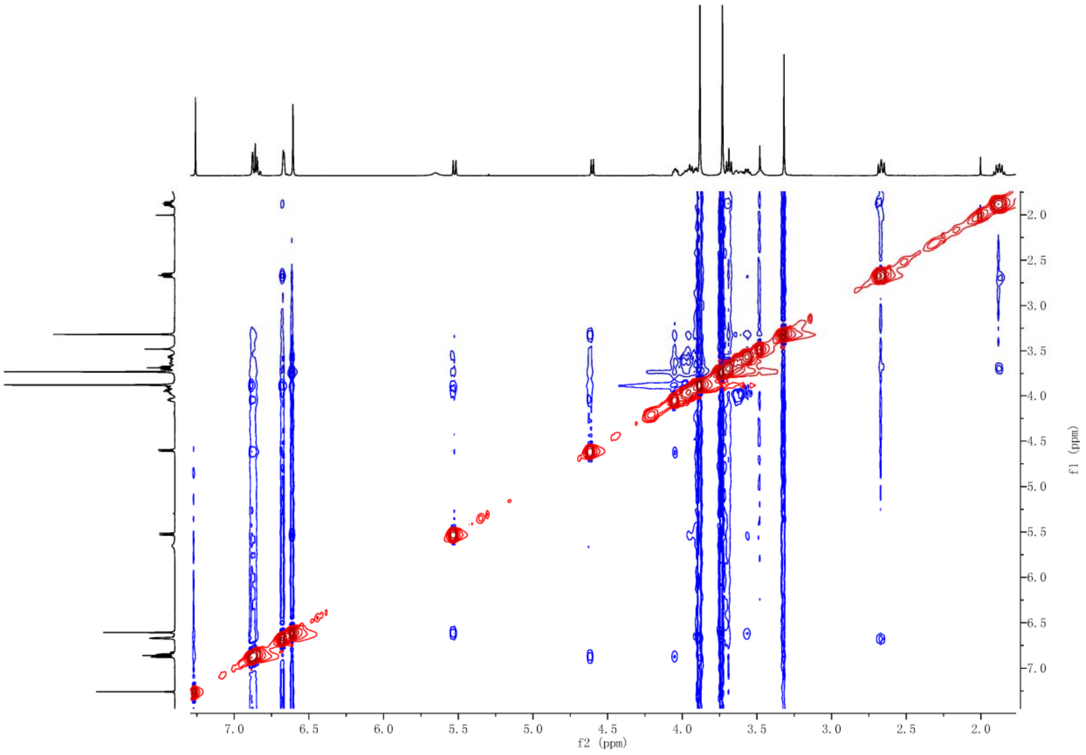

^{1}H NMR of compound 5 in Acetone-d_6(400 MHz)

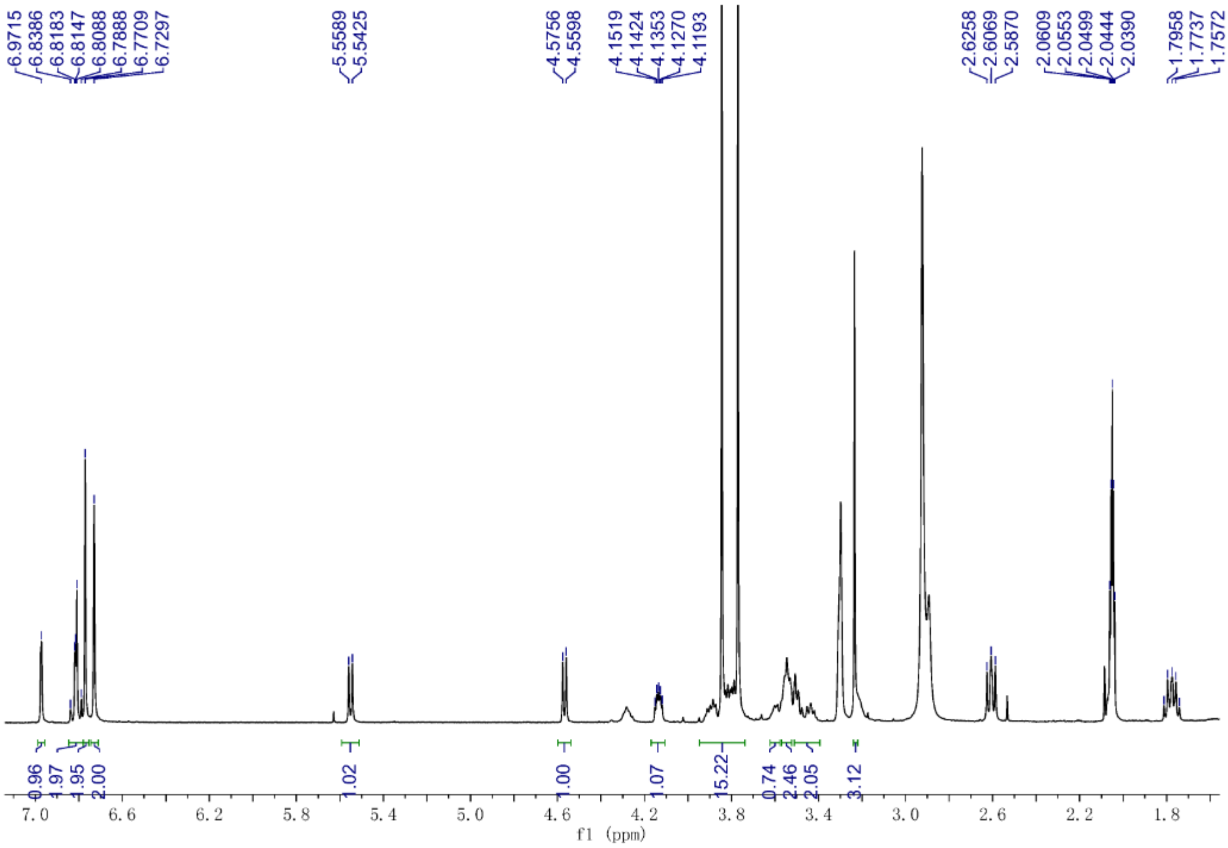

HRESIMS of compound 6

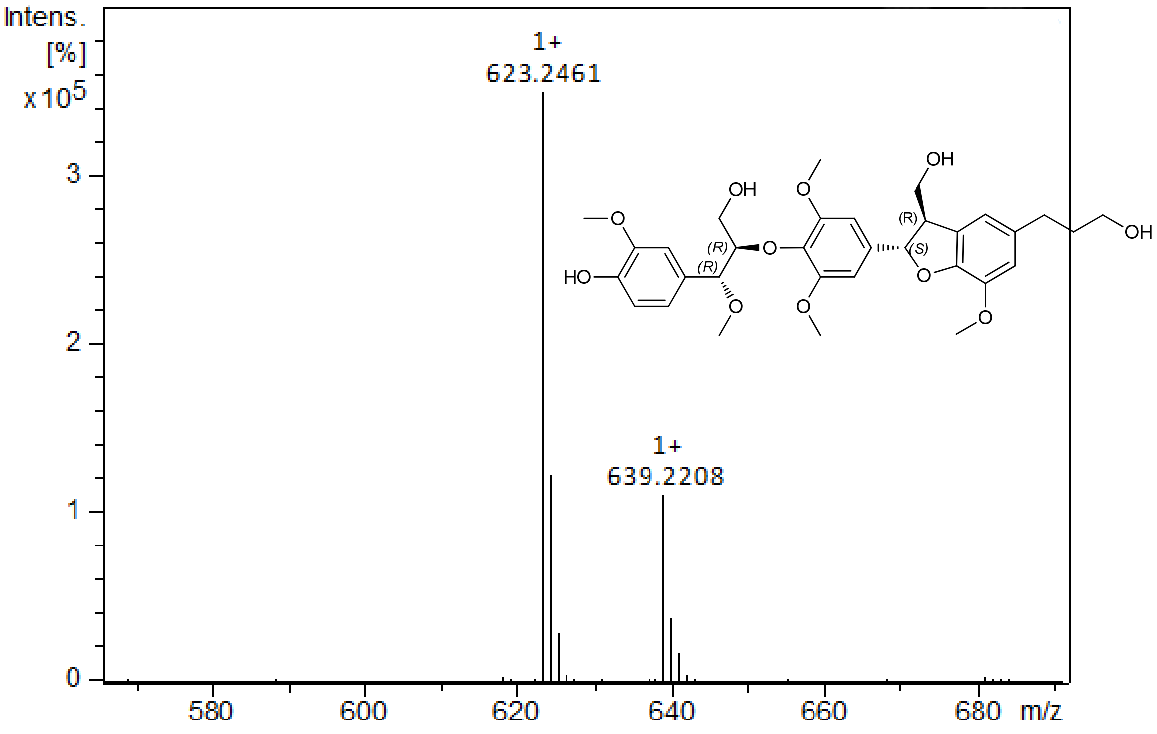

UV spectrum of compound 6

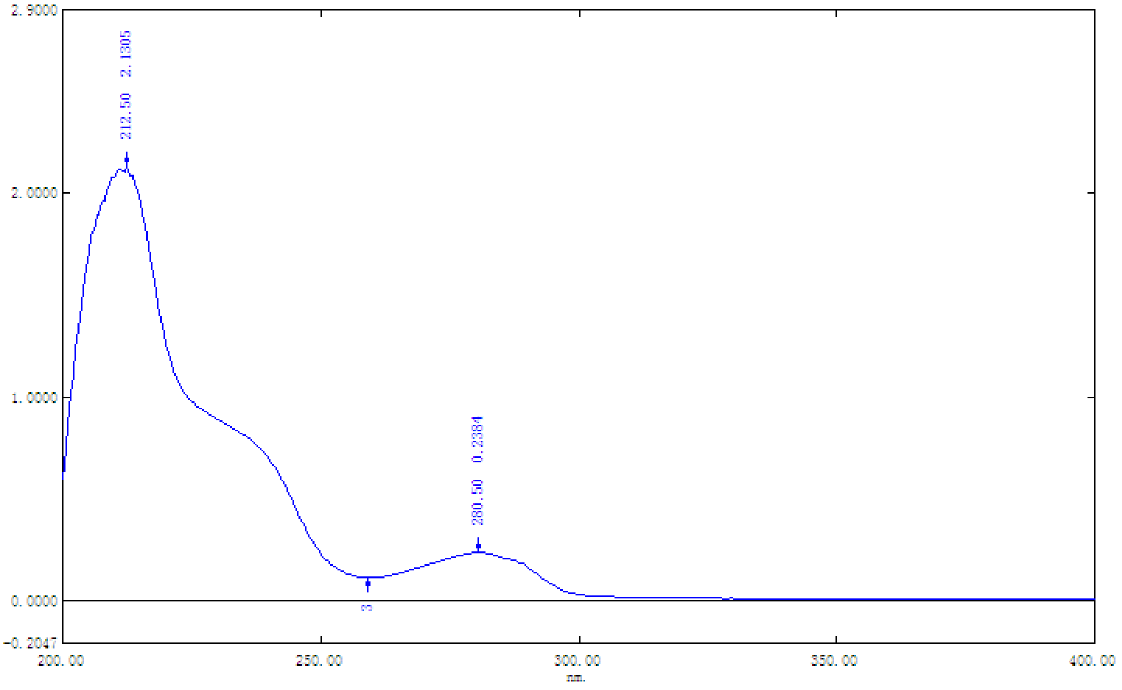

IR spectrum of compound 6

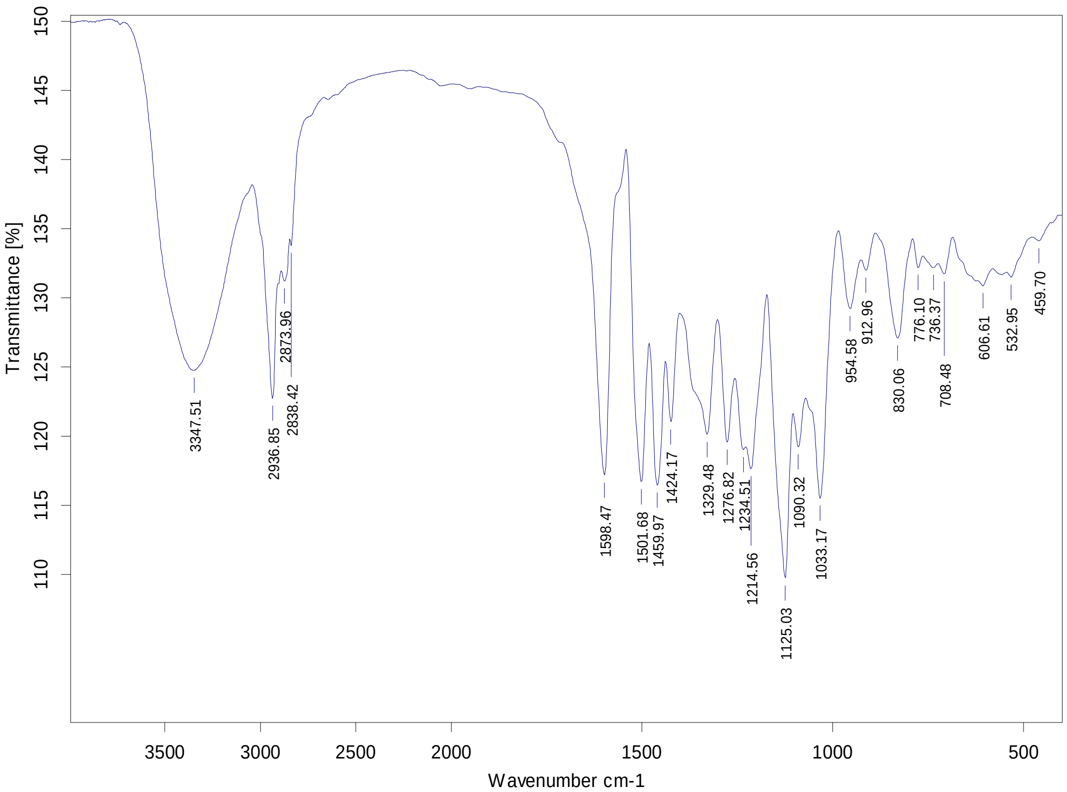

^{1}H NMR of compound 6

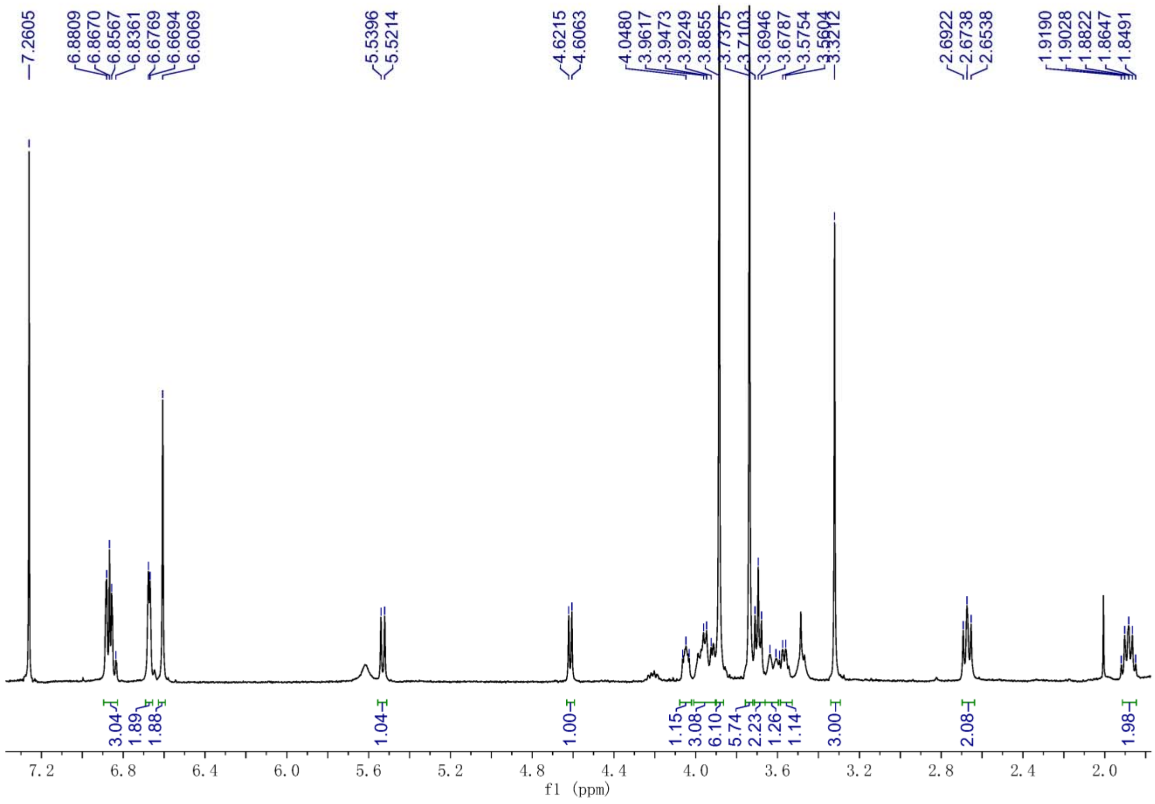

^{13}C NMR and DEPT of compound 6

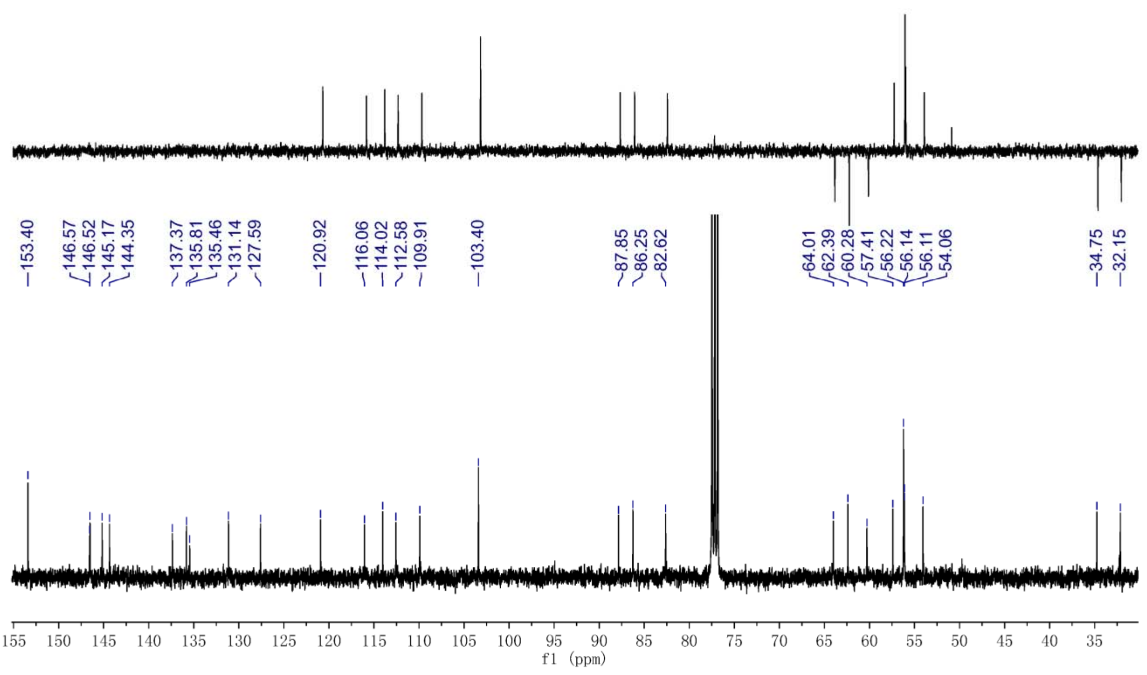

HSQC of compound 6

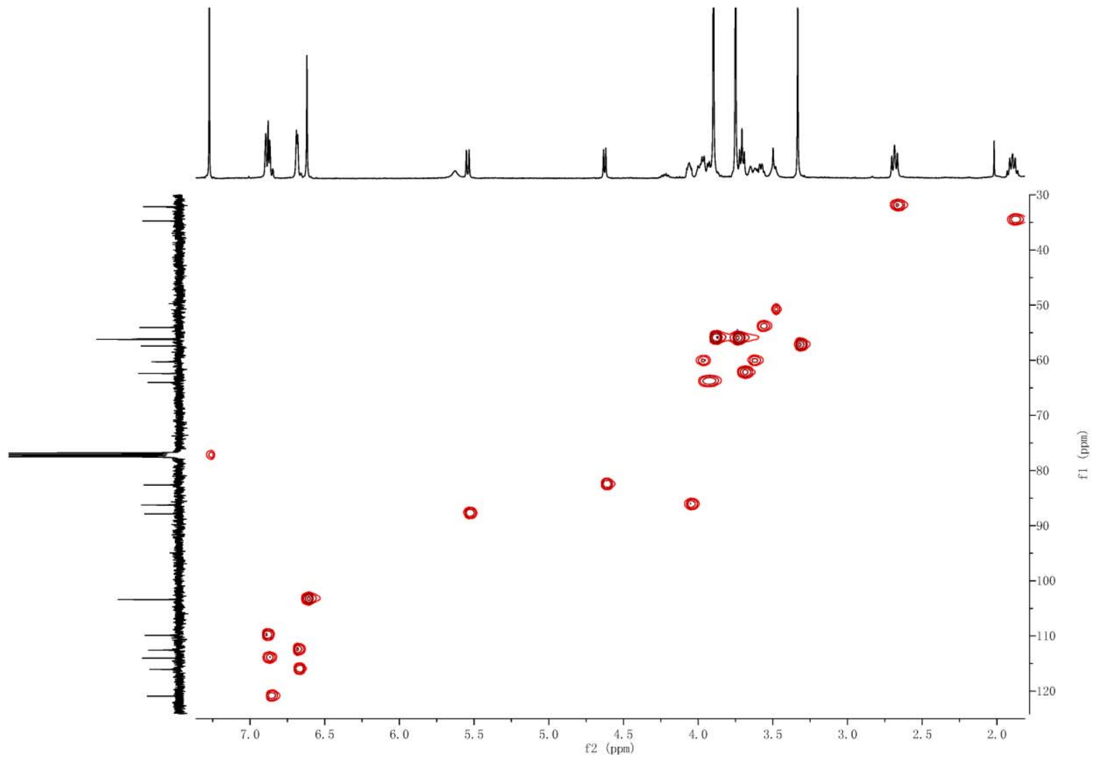

HMBC of compound 6

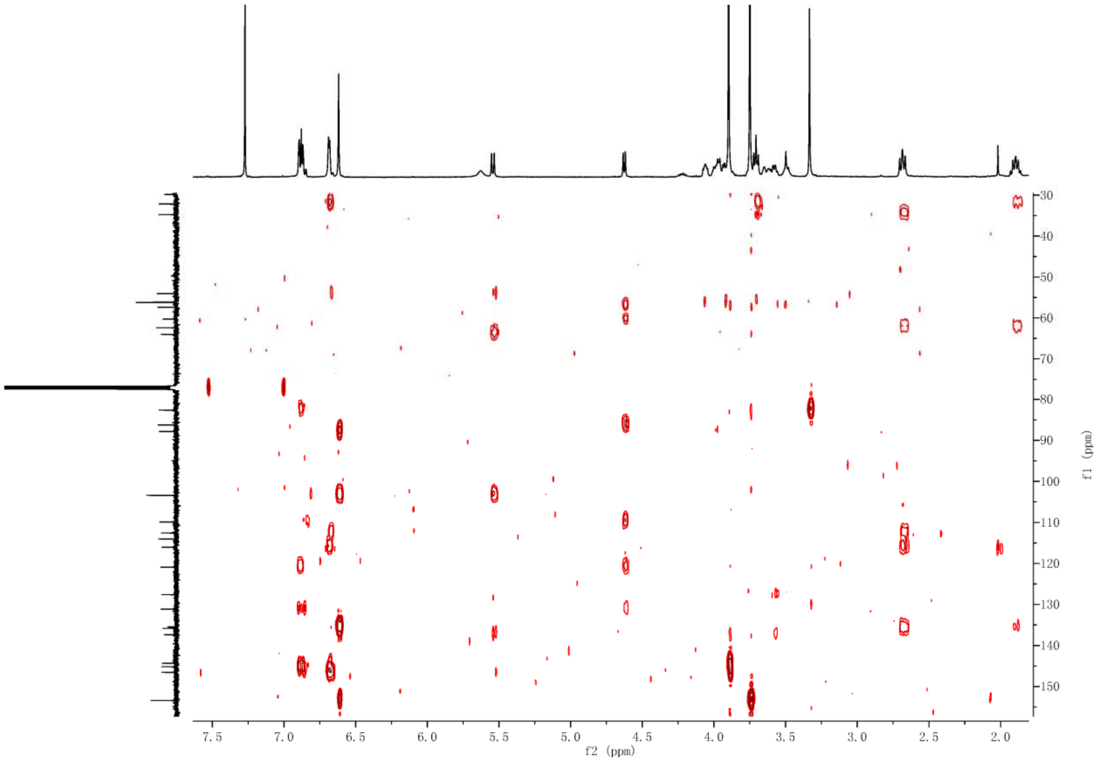

^{1}H–^{1}H COSY of compound 6

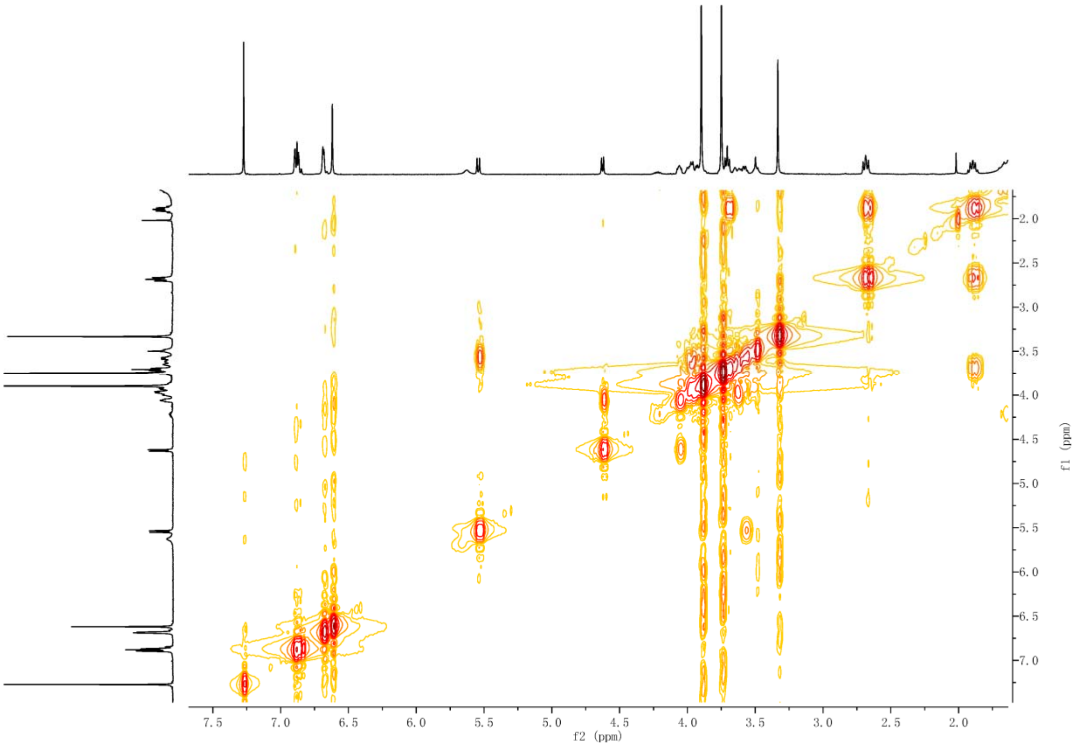

NOESY of compound 6

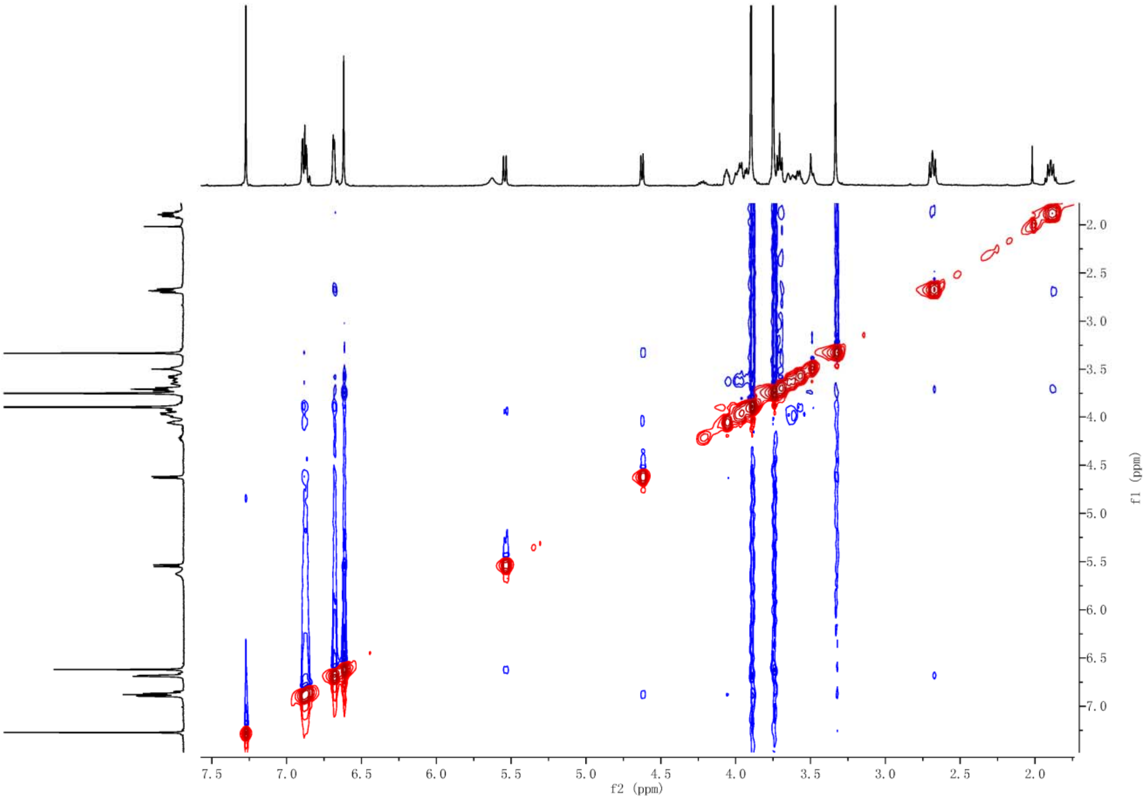

^{1}H NMR of compound 6 in Acetone-d_6(400 MHz)

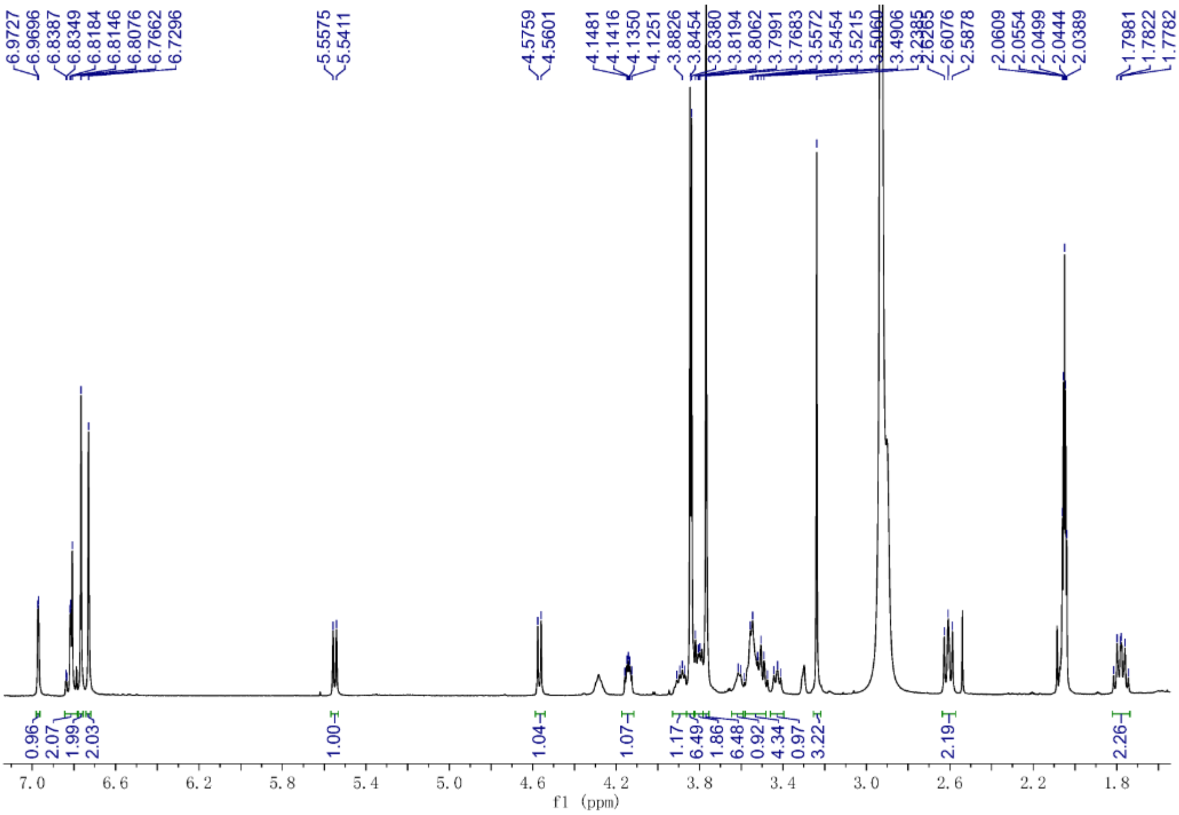

HRESIMS of compound 7

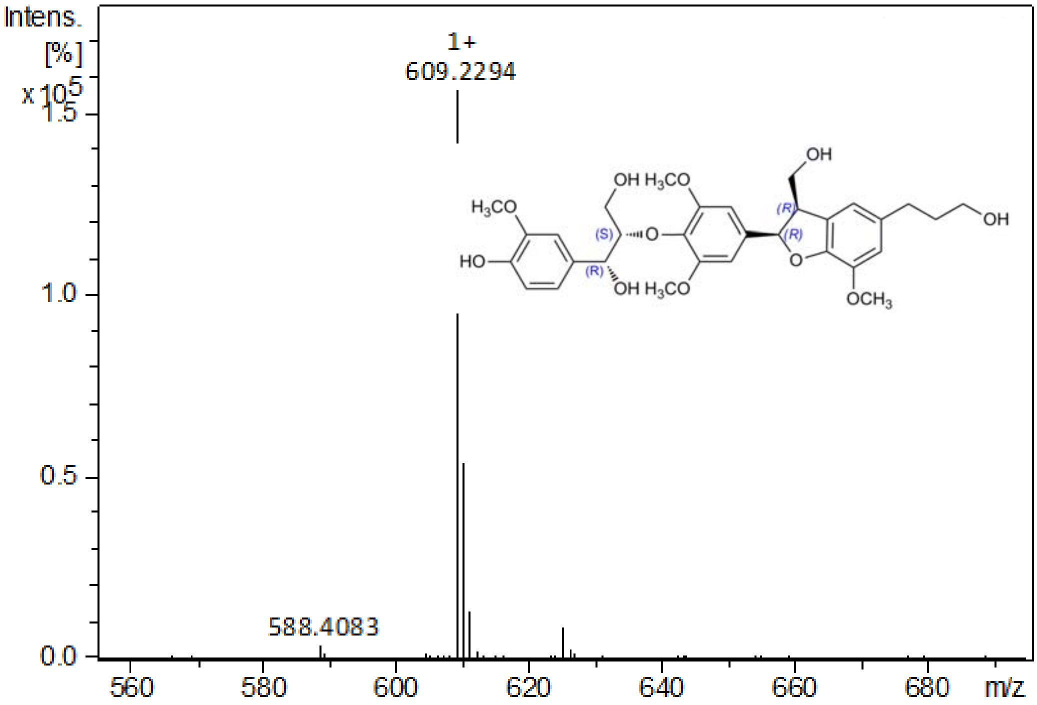

UV spectrum of compound 7

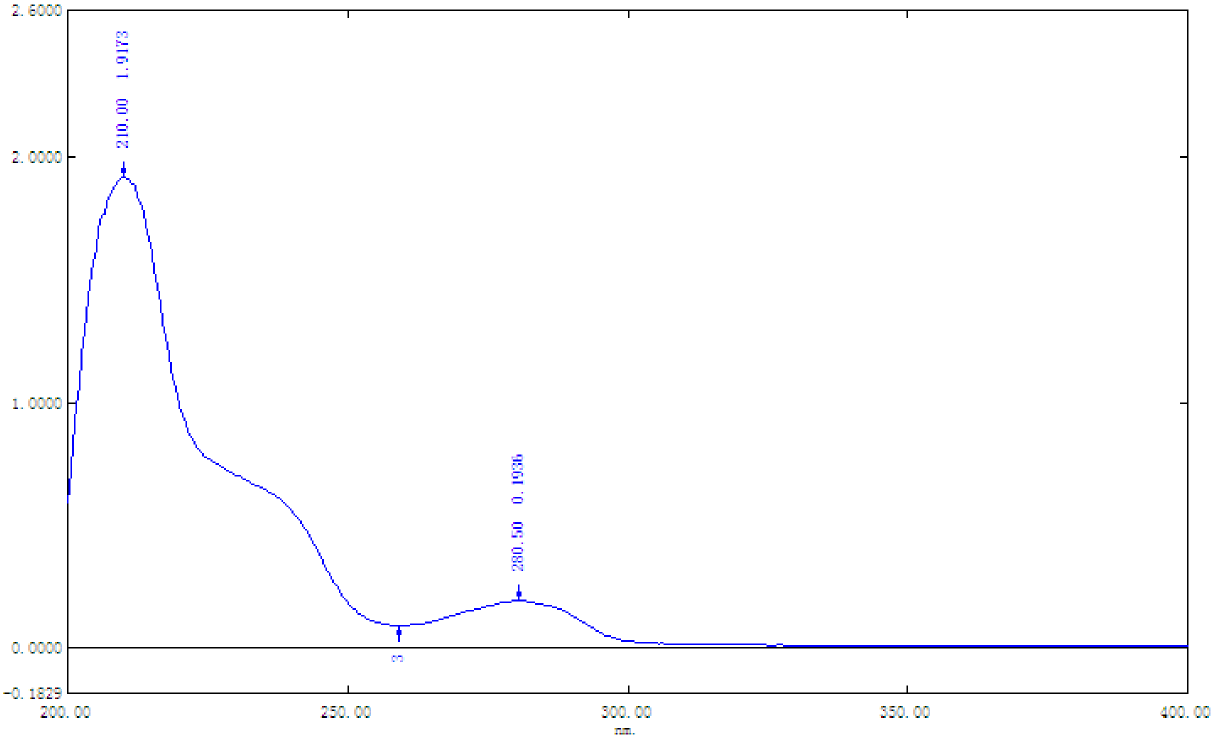

IR spectrum of compound 7

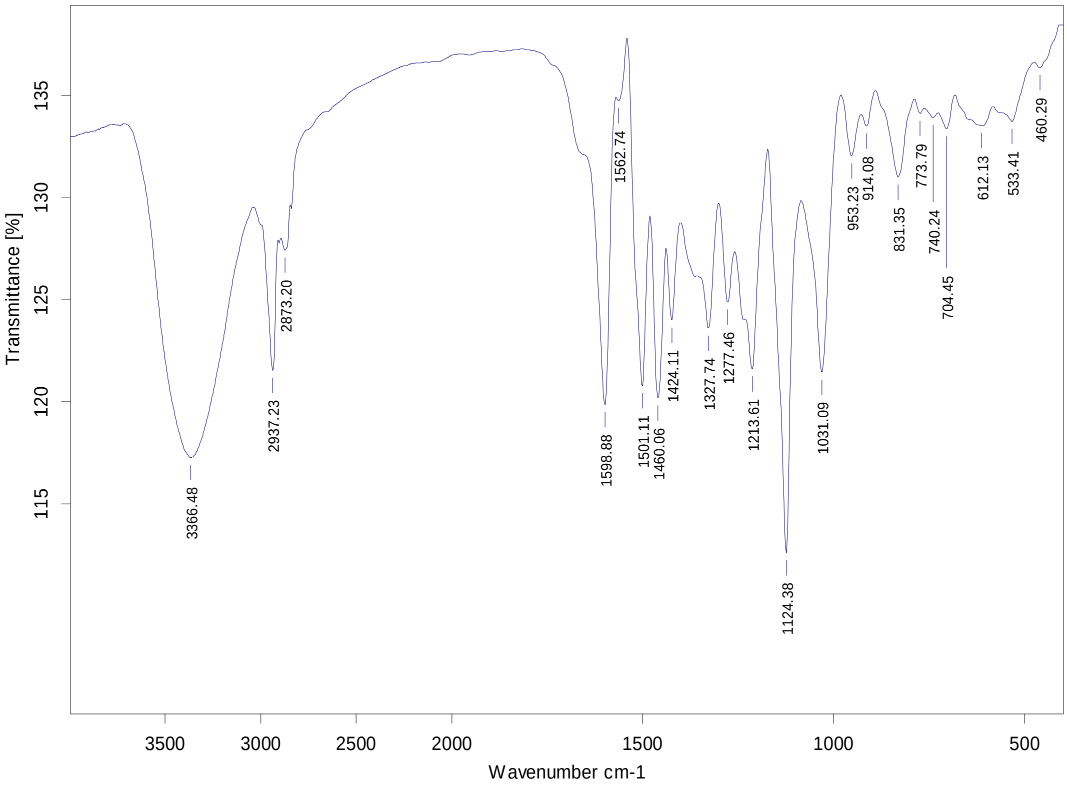

^{1}H NMR of compound 7

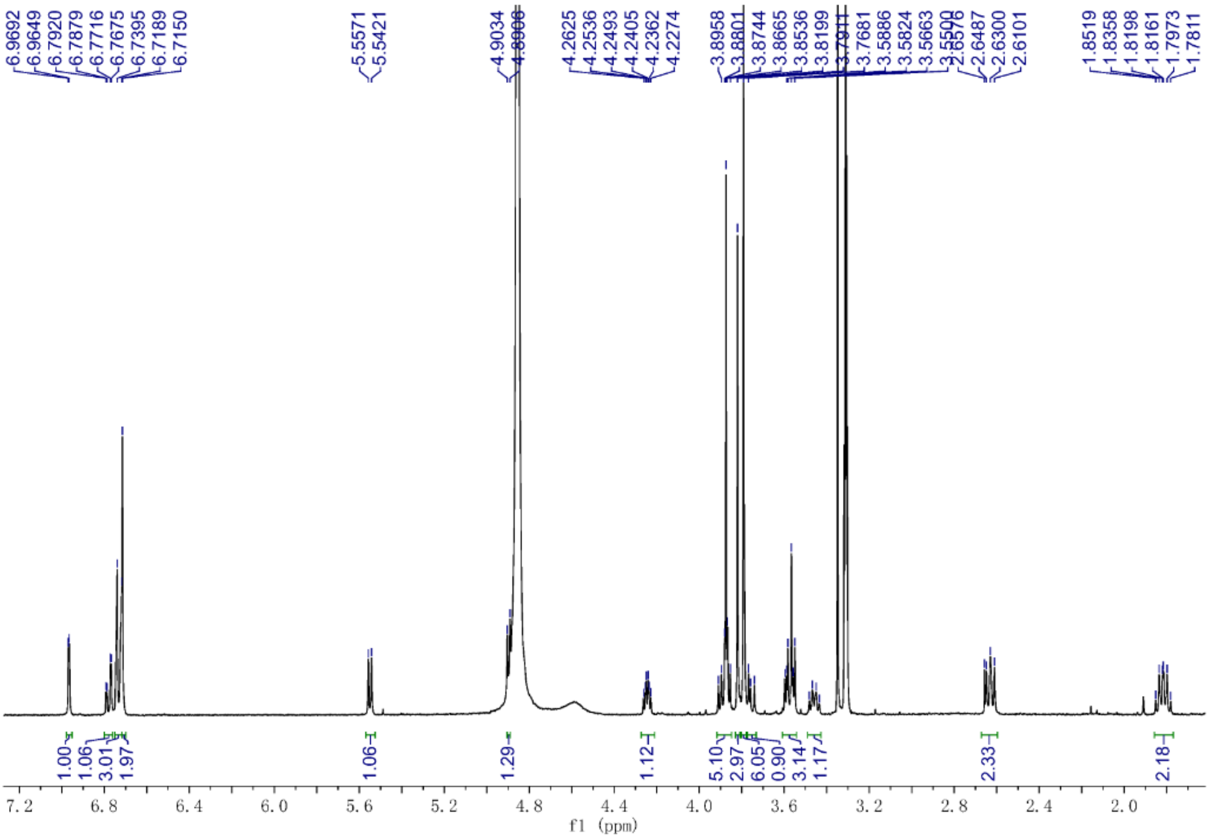

^{13}C NMR and DEPT of compound 7

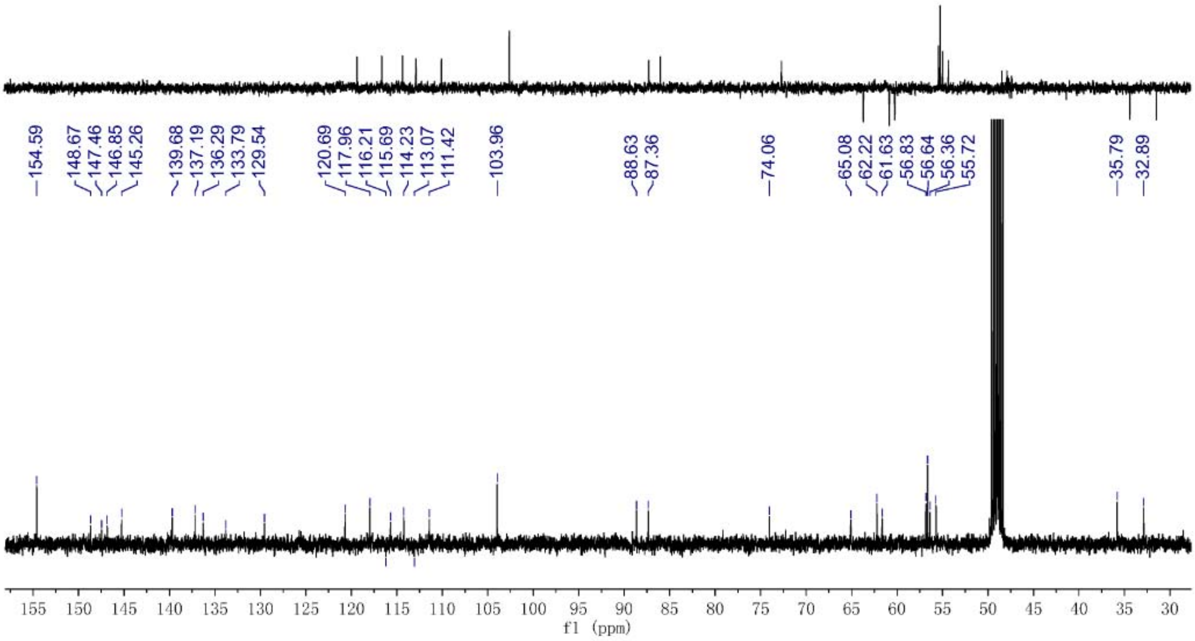

HSQC of compound 7

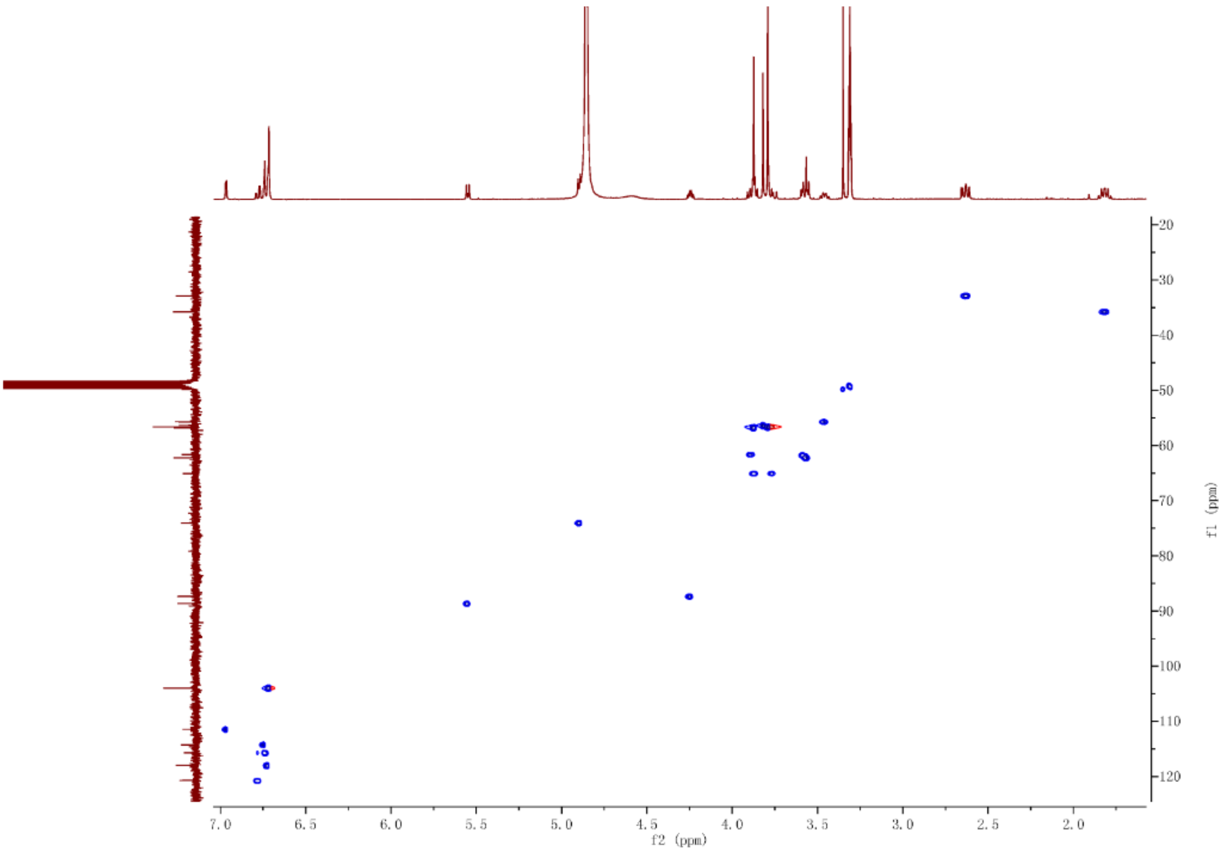

HMBC of compound 7

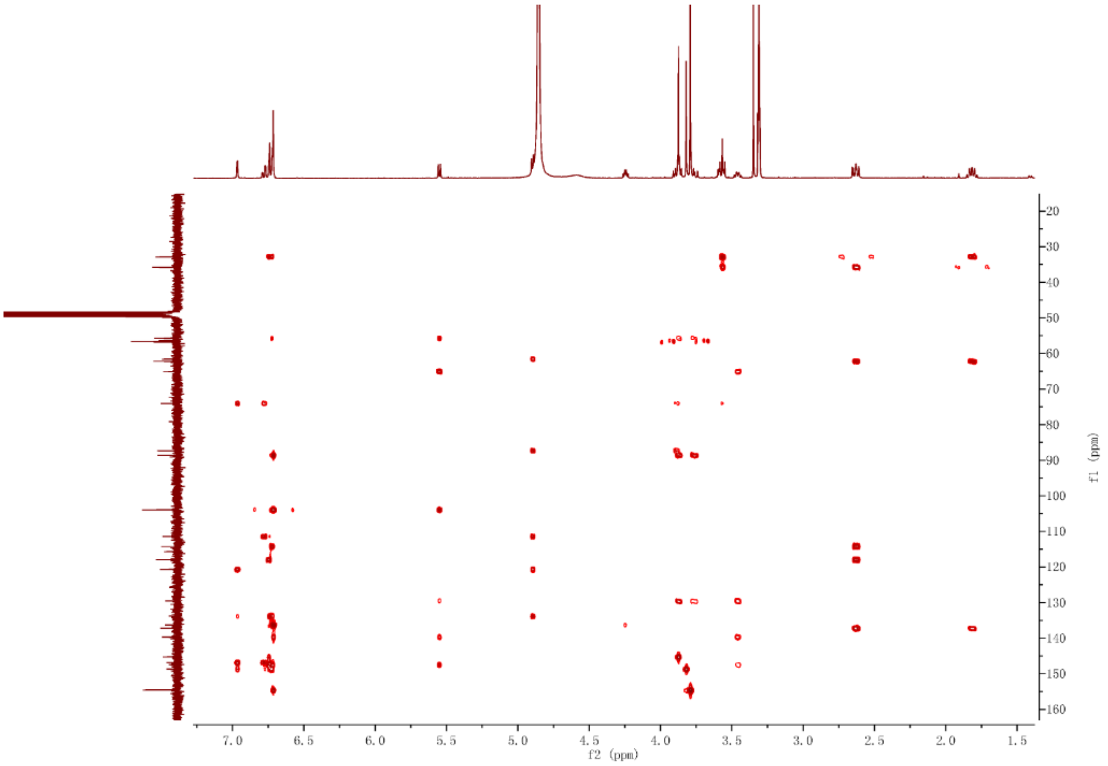

^{1}H–^{1}H COSY of compound 7

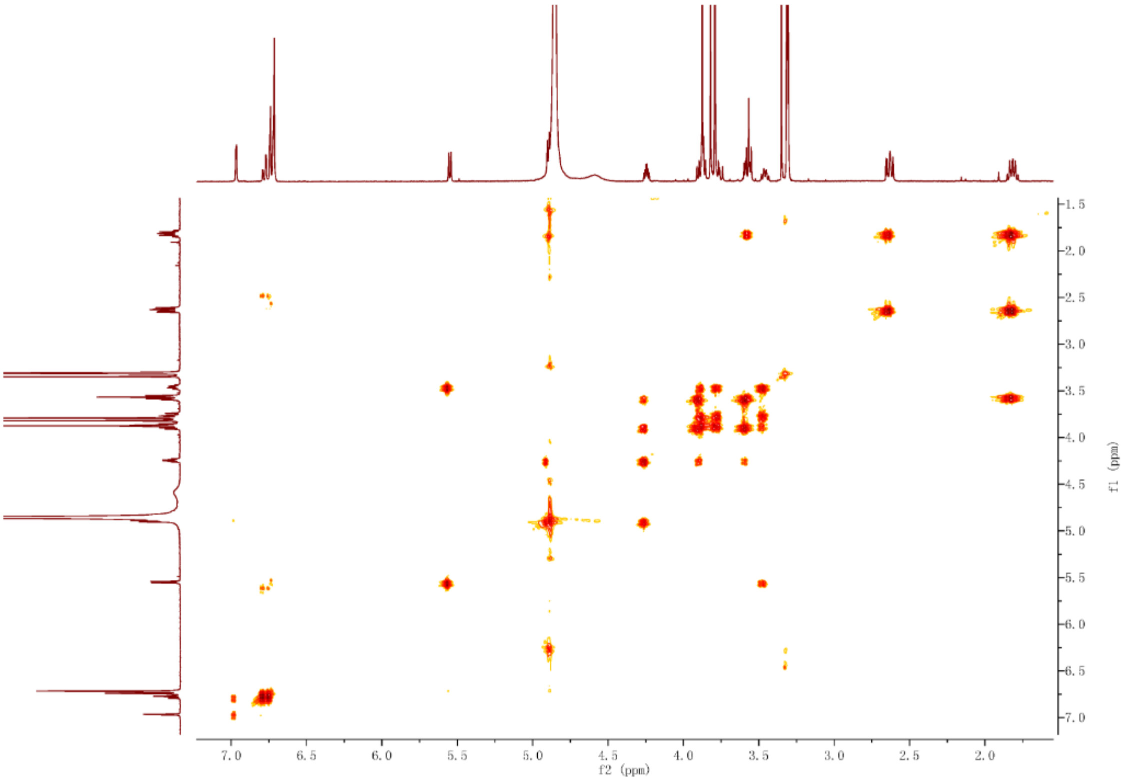

NOESY of compound 7

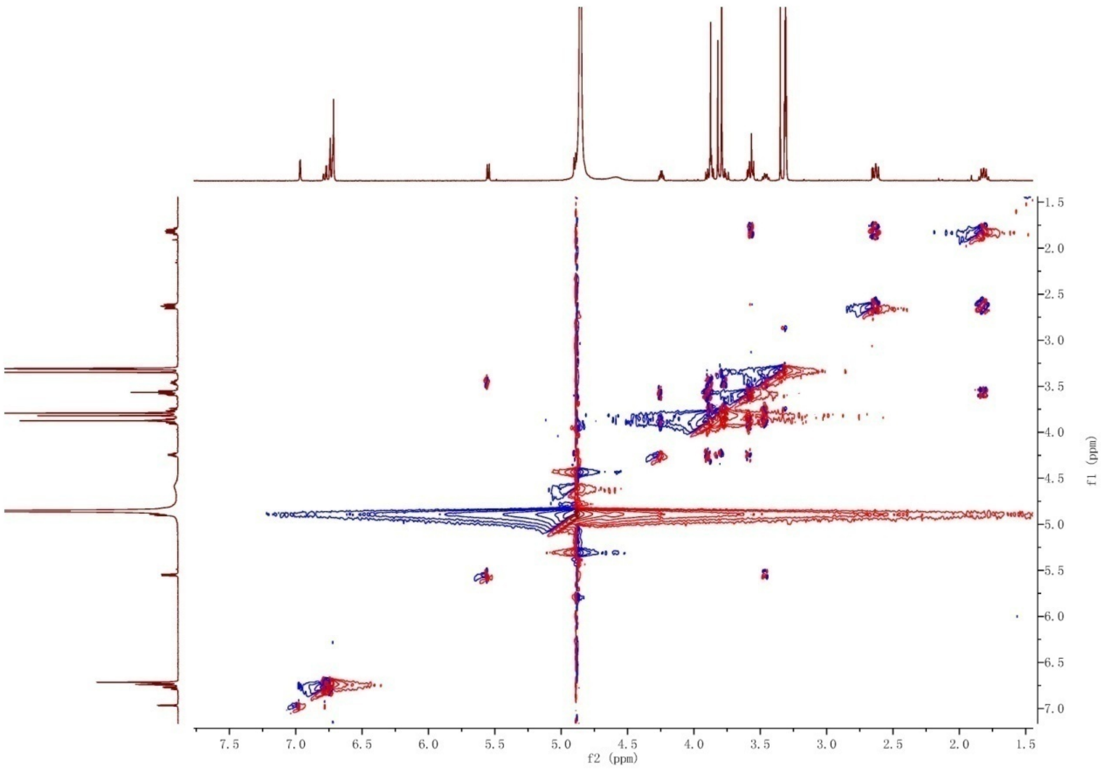

^{1}H NMR of compound 7 in Acetone-d_6(400 MHz)

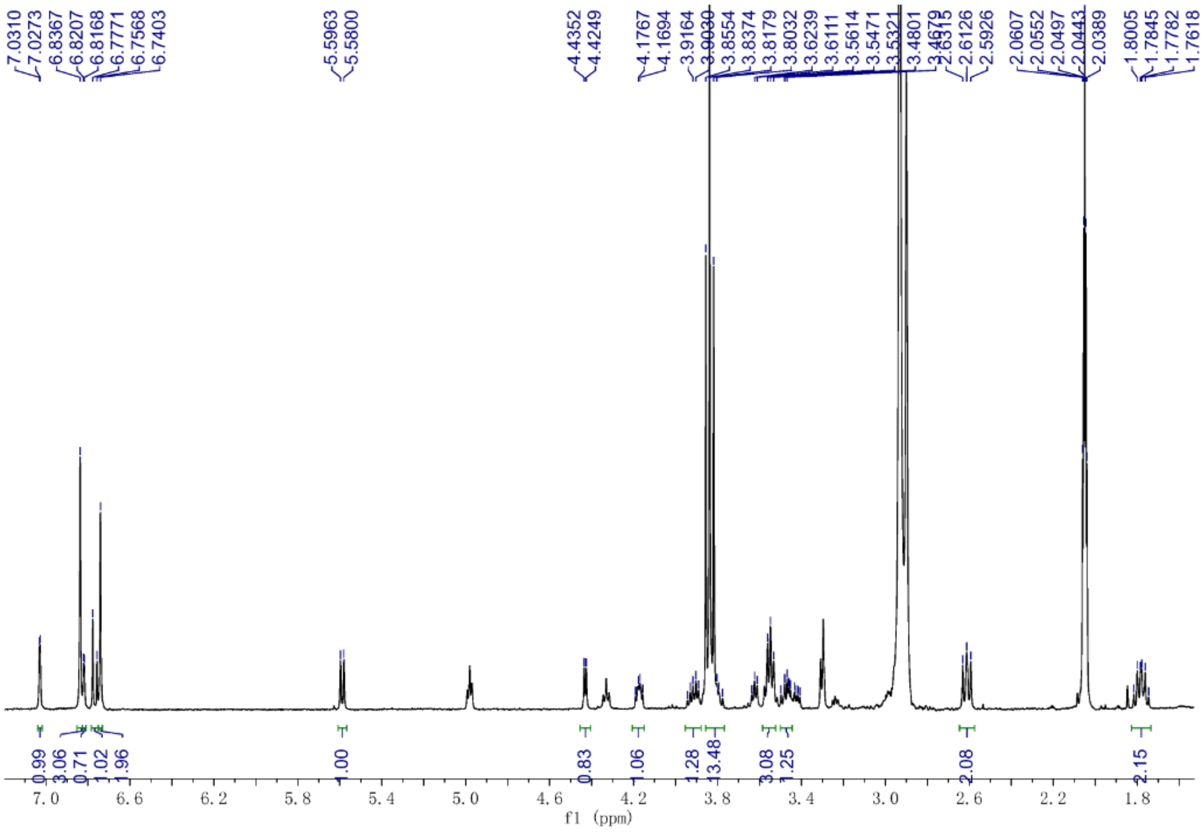

HRESIMS of compound 8

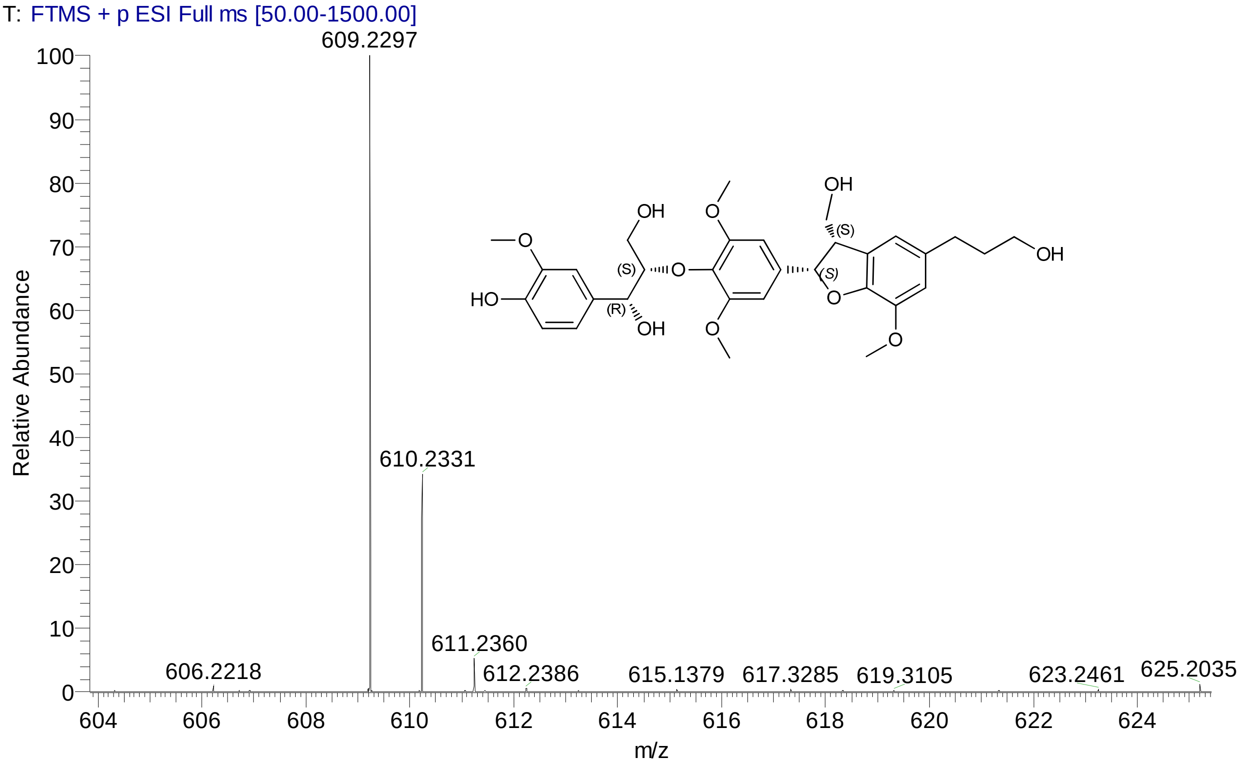

UV spectrum of compound 8

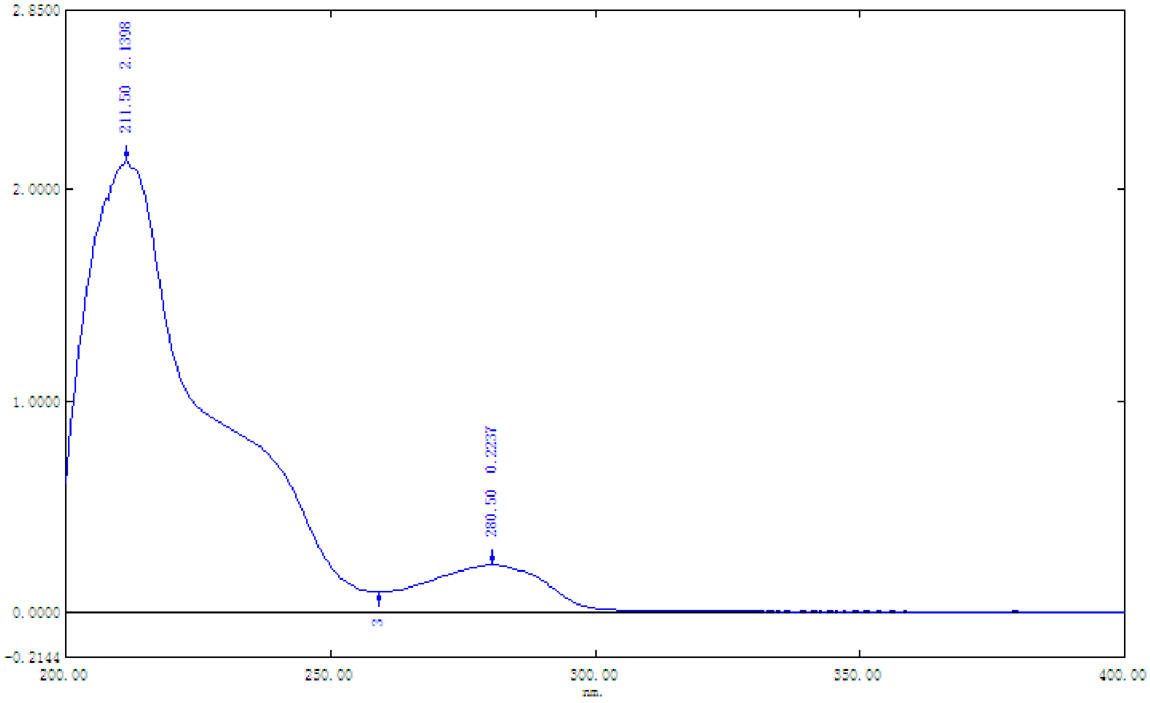

IR spectrum of compound 8

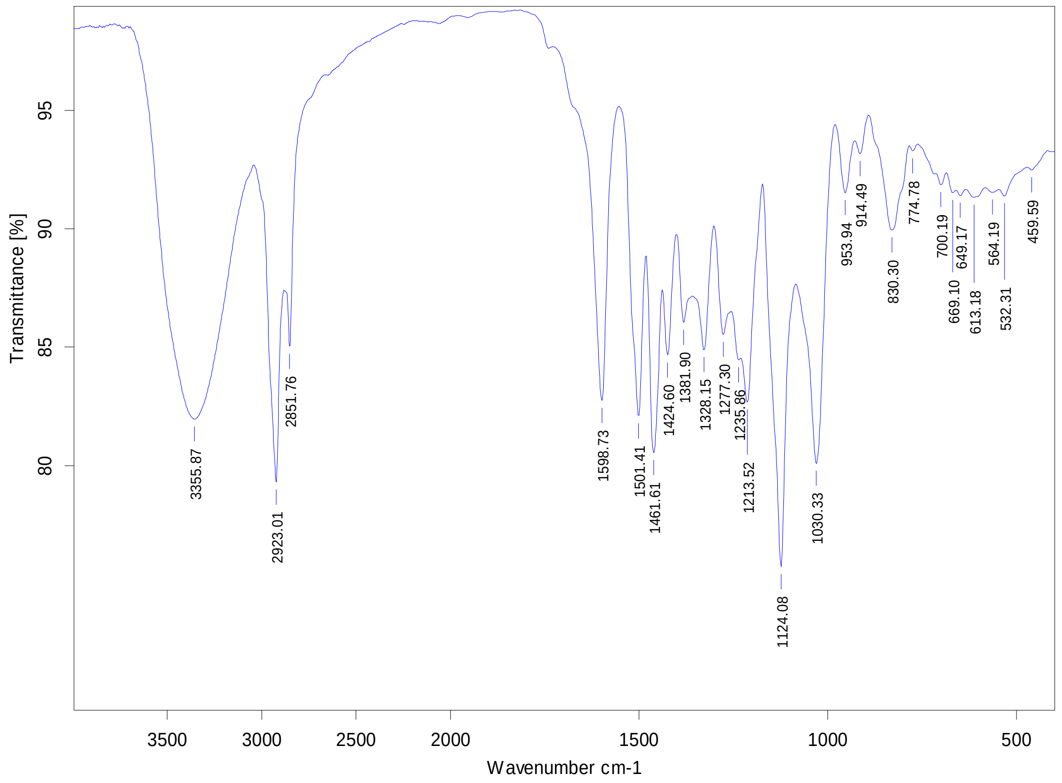

^{1}H NMR of compound 8

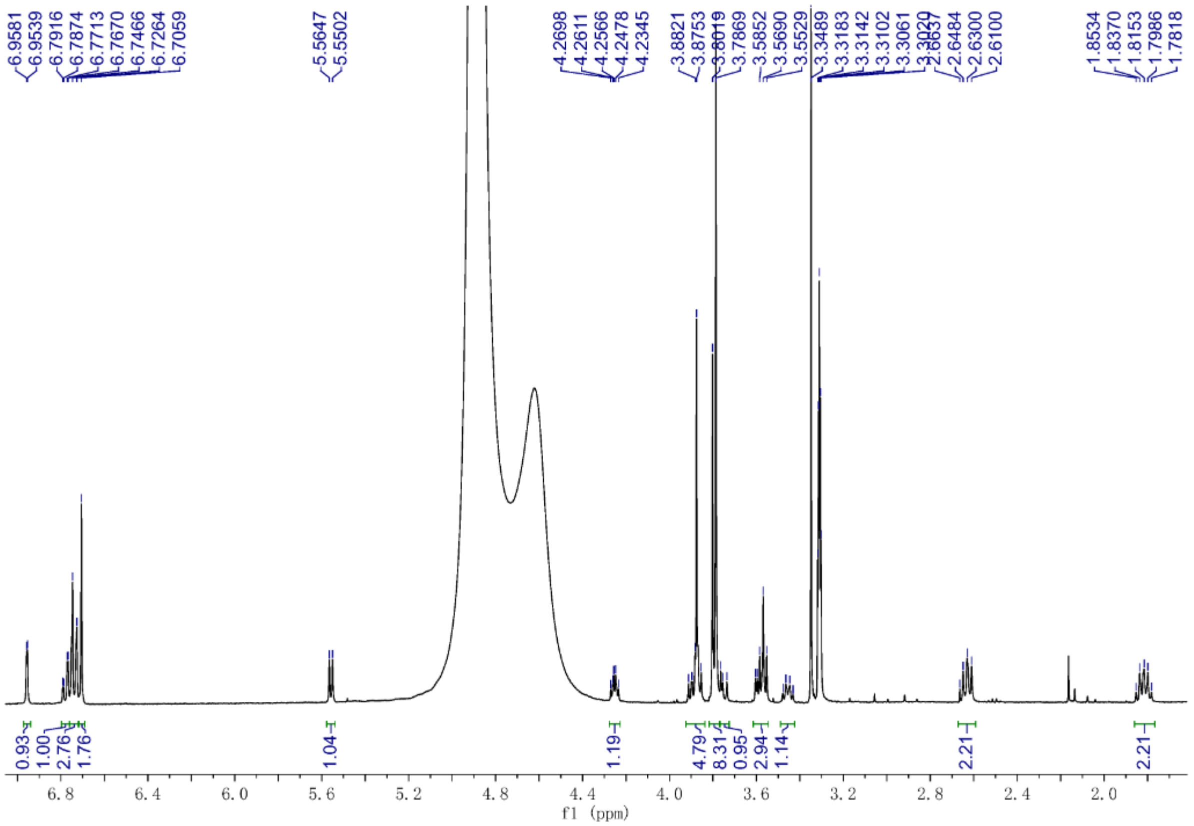

^{13}C NMR and DEPT of compound 8

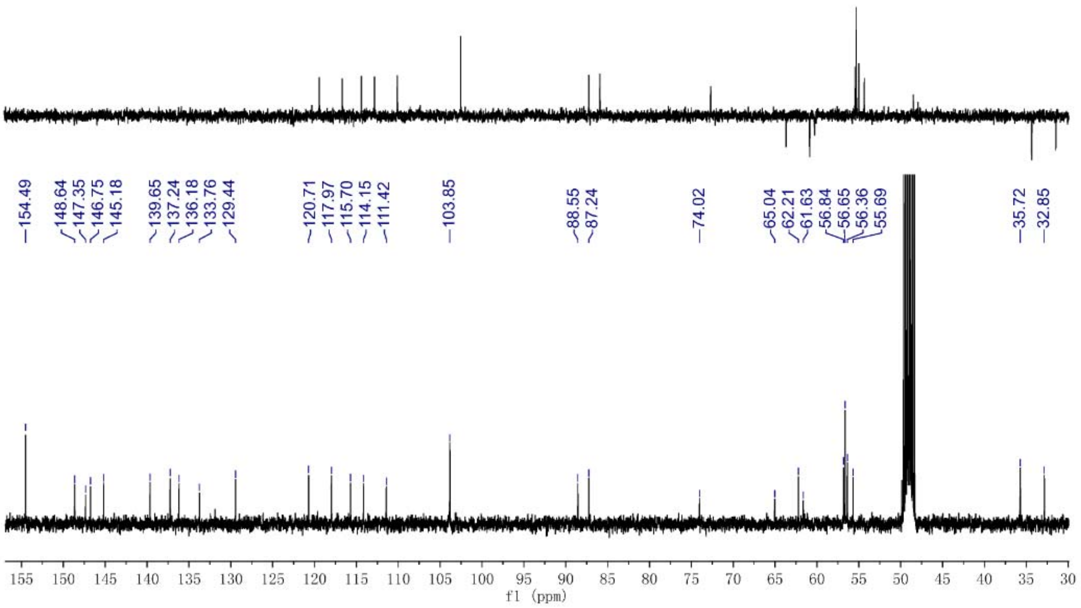

HSQC of compound 8

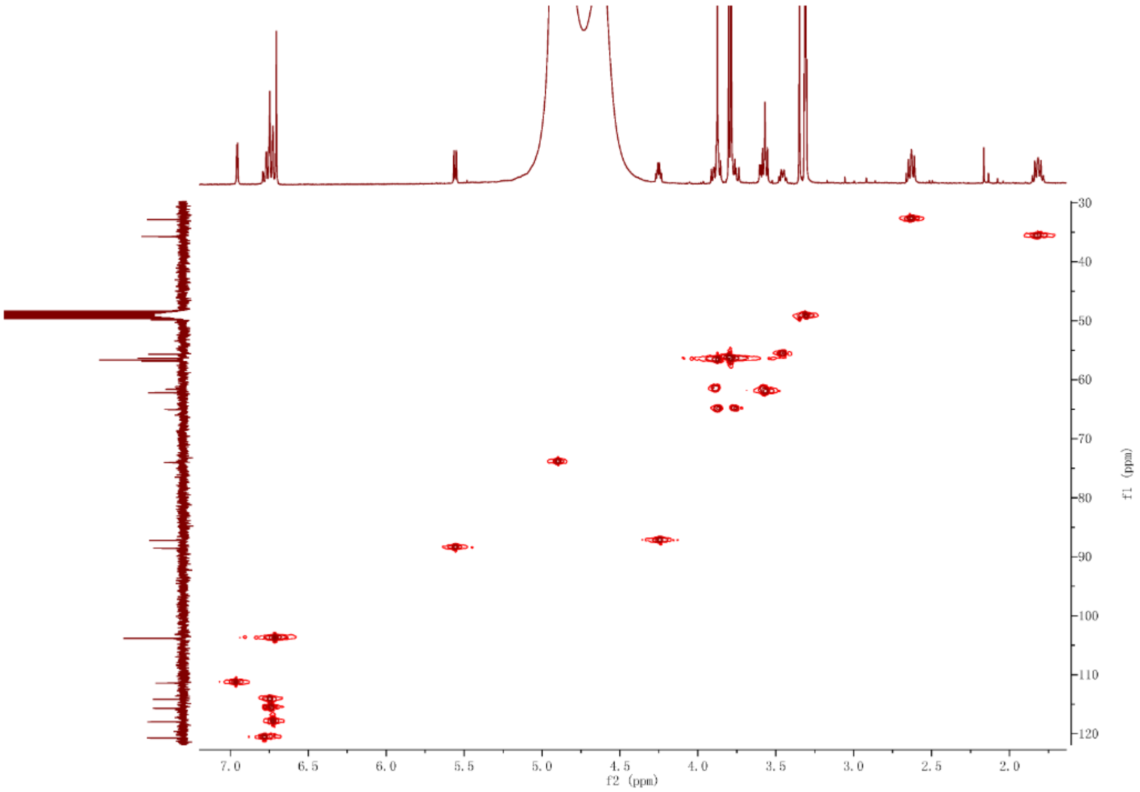

HMBC of compound 8

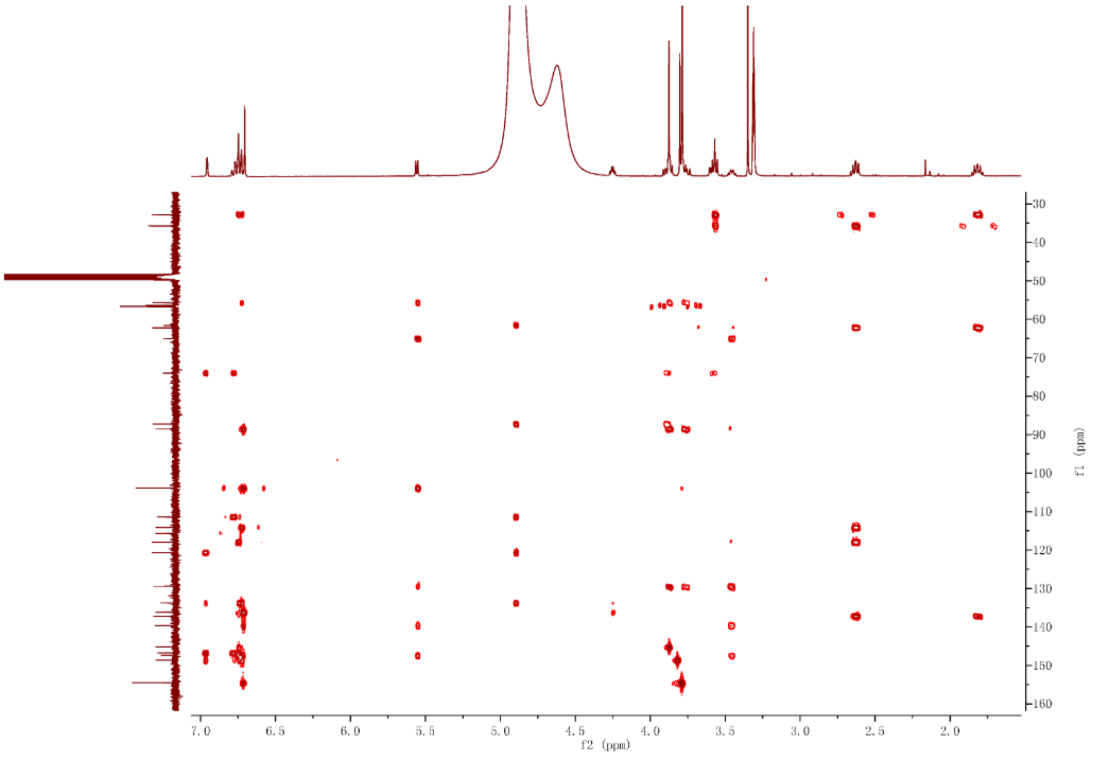

¹H–¹H COSY of compound 8

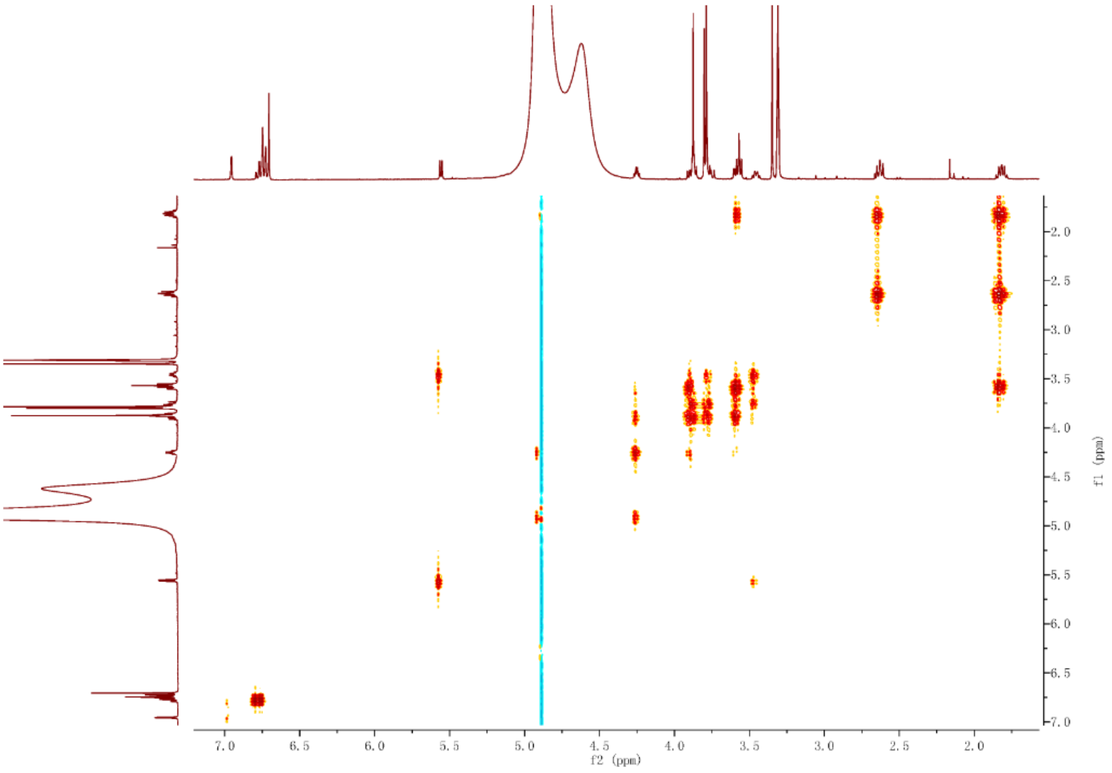

NOESY of compound 8

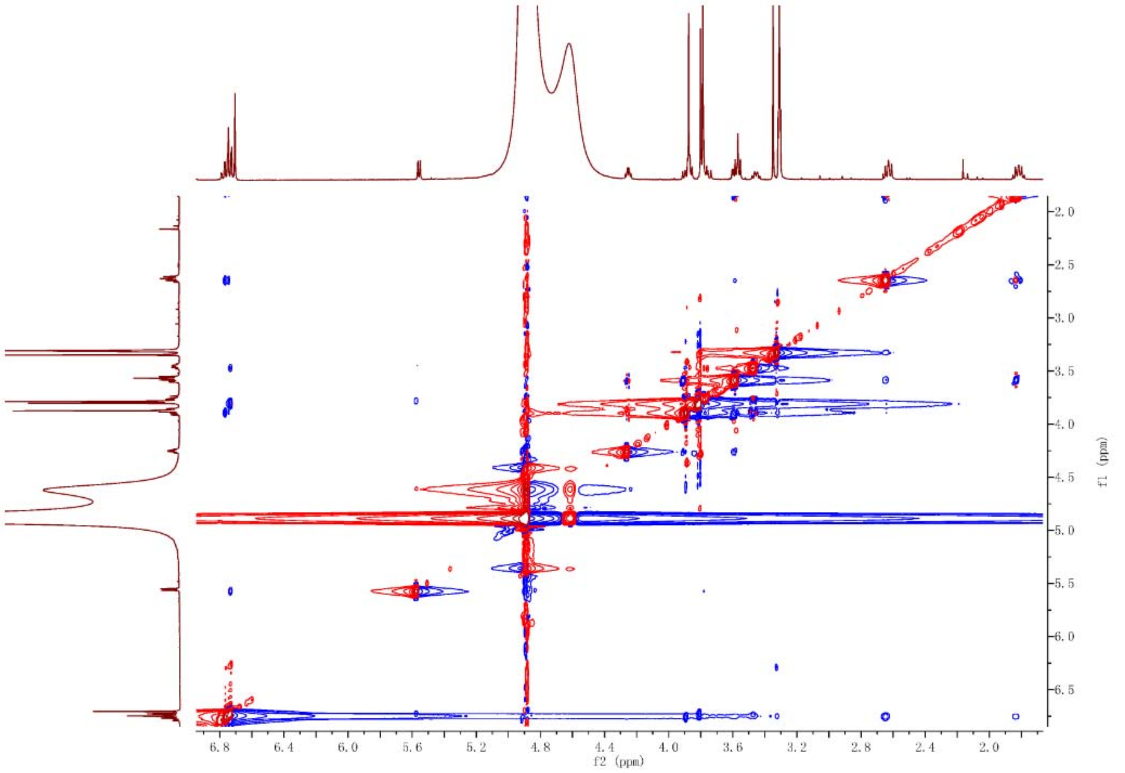

^{1}H NMR of compound 8 in Acetone-d_6(400 MHz)

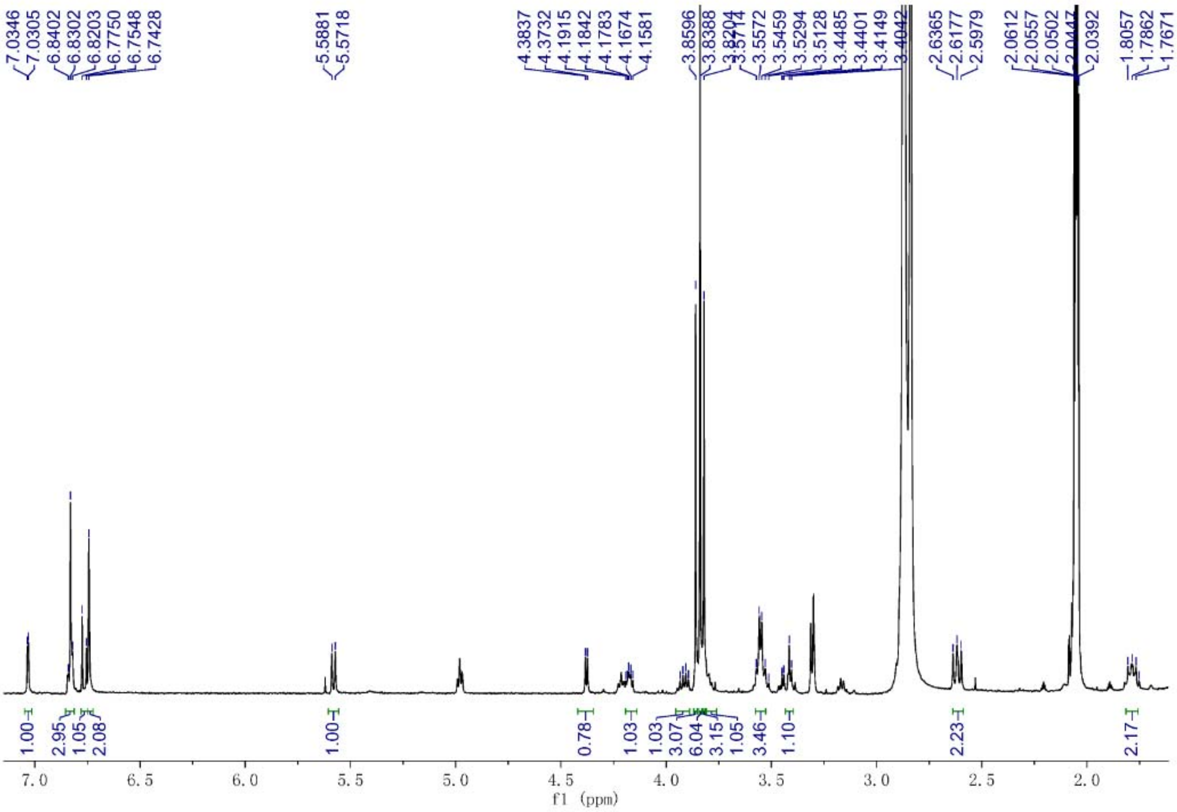

Chiral HPLC separation profiles of 1a/1b−8a/8b

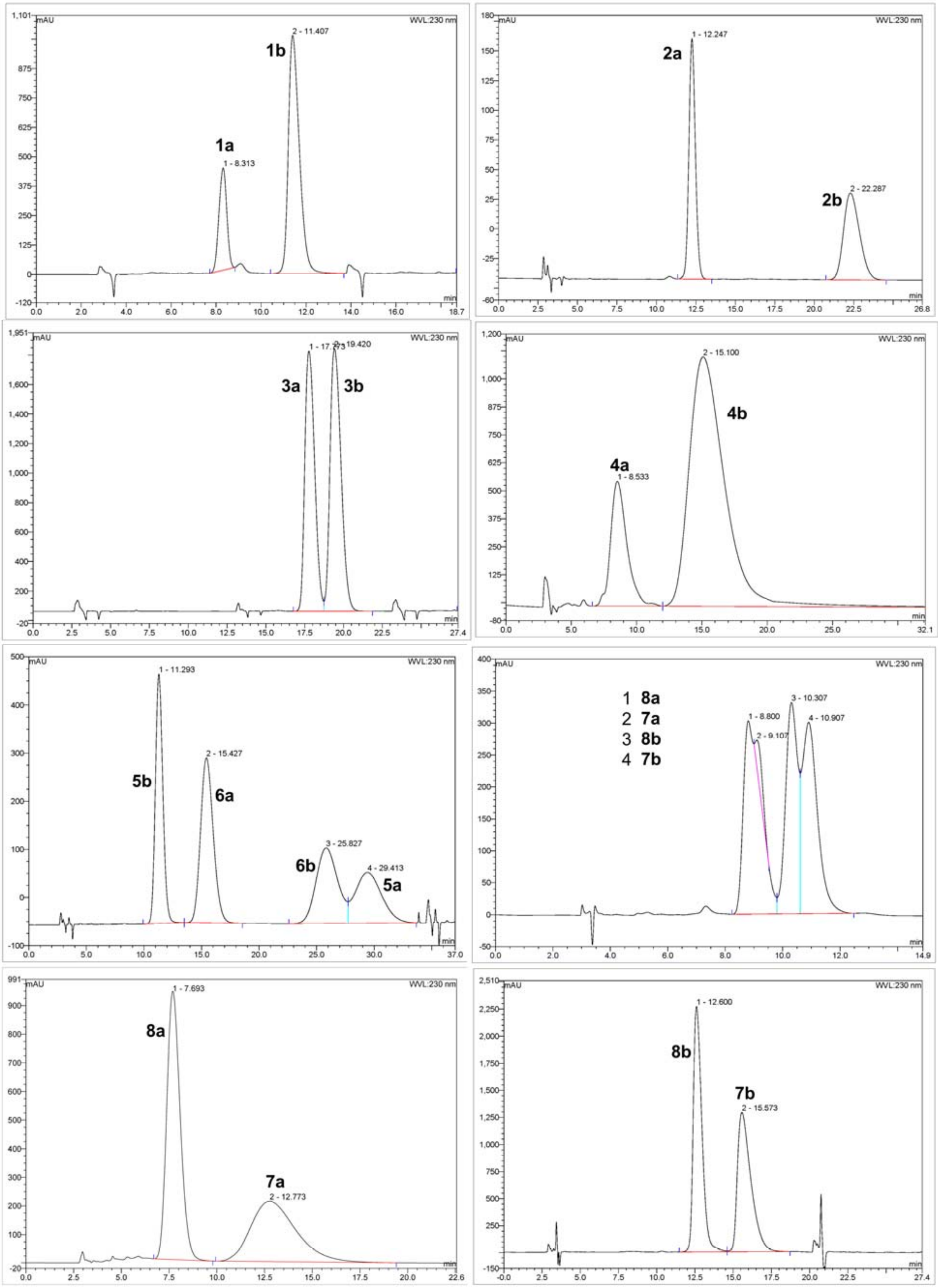

Supplement: Supplementary Information [file srep24809-s1.pdf]
